# *LEAFY* maintains apical stem cell activity during shoot development in the fern *Ceratopteris richardii* 

**DOI:** 10.7554/eLife.39625

**Published:** 2018-10-24

**Authors:** Andrew RG Plackett, Stephanie J Conway, Kristen D Hewett Hazelton, Ester H Rabbinowitsch, Jane A Langdale, Verónica S Di Stilio

**Affiliations:** 1 Department of Plant SciencesUniversity of OxfordOxfordUnited Kingdom; 2 Department of BiologyUniversity of WashingtonSeattleUnited States; University of LausanneSwitzerland; University of California-BerkeleyUnited States

**Keywords:** ferns, Ceratopteris richardii, LEAFY, shoot development, land plant evolution, apical cells, Other

## Abstract

During land plant evolution, determinate spore-bearing axes (retained in extant bryophytes such as mosses) were progressively transformed into indeterminate branching shoots with specialized reproductive axes that form flowers. The LEAFY transcription factor, which is required for the first zygotic cell division in mosses and primarily for floral meristem identity in flowering plants, may have facilitated developmental innovations during these transitions. Mapping the LEAFY evolutionary trajectory has been challenging, however, because there is no functional overlap between mosses and flowering plants, and no functional data from intervening lineages. Here, we report a transgenic analysis in the fern *Ceratopteris richardii* that reveals a role for LEAFY in maintaining cell divisions in the apical stem cells of both haploid and diploid phases of the lifecycle. These results support an evolutionary trajectory in which an ancestral LEAFY module that promotes cell proliferation was progressively co-opted, adapted and specialized as novel shoot developmental contexts emerged.

## eLife digest

The first plants colonized land around 500 million years ago. These plants had simple shoots with no branches, similar to the mosses that live today. Later on, some plants evolved more complex structures including branched shoots and flowers (collectively known as the “flowering plants”). Ferns are a group of plants that evolved midway between the mosses and flowering plants and have branched shoots but no flowers.

The gradual transition from simple to more complex plant structures required changes to the way in which cells divide and grow within plant shoots. Whereas animals produce new cells throughout their body, most plant cells divide in areas known as meristems. All plants grow from embryos, which contain meristems that will form the roots and shoots of the mature plant. A gene called *LEAFY* is required for cells in moss embryos to divide. However, in flowering plants *LEAFY* does not carry out this role, instead it is only required to make the meristems that produce flowers.

How did *LEAFY* transition from a general role in embryos to a more specialized role in making flowers? To address this question, Plackett, Conway et al. studied the two *LEAFY* genes in a fern called *Ceratopteris richardii*. The experiments showed that at least one of these *LEAFY* genes was active in the meristems of fern shoots throughout the lifespan of the plant. The shoots of ferns with less active *LEAFY* genes could not form the leaves seen in normal *C. richardii* plants. This suggests that as land plants evolved, the role of *LEAFY* changed from forming embryos to forming complex shoot structures.

Most of our major crops are flowering plants. By understanding how the role of *LEAFY* has changed over the evolution of land plants, it might be possible to manipulate *LEAFY* genes in crop plants to alter shoot structures to better suit specific environments.

## Introduction

Land plants are characterized by the alternation of haploid (gametophyte) and diploid (sporophyte) phases within their lifecycle, both of which are multicellular (Niklas and Kutschera, 2010; Bowman et al., 2016). In the earliest diverging bryophyte lineages (liverworts, mosses and hornworts) the free-living indeterminate gametophyte predominates the lifecycle, producing gametes that fuse to form the sporophyte. The sporophyte embryo develops on the surface of the gametophyte, ultimately forming a simple determinate spore-producing axis (Kato and Akiyama, 2005; Ligrone et al., 2012). By contrast, angiosperm (flowering plant) sporophytes range from small herbaceous to large arborescent forms, all developing from an indeterminate vegetative shoot apex that ultimately transitions to flowering, and gametophytes are few-celled determinate structures produced within flowers (Schmidt et al., 2015). A series of developmental innovations during the course of land plant evolution thus simplified gametophyte form whilst increasing sporophyte complexity, with a prolonged and plastic phase of vegetative development arising in the sporophyte of all vascular plants (lycophytes, ferns, gymnosperms and angiosperms).

Studies aimed at understanding how gene function evolved to facilitate developmental innovations during land plant evolution have thus far largely relied on comparative analyses between bryophytes and angiosperms, lineages that diverged over 450 million years ago. Such comparisons have revealed examples of both sub- and neo-functionalization following gene duplication, and of co-option of existing gene regulatory networks into new developmental contexts. For example, a single bHLH transcription factor in the moss *Physcomitrella patens* regulates stomatal differentiation, whereas gene duplications have resulted in three homologs with sub-divided stomatal patterning roles in the angiosperm *Arabidopsis thaliana* (hereafter ‘Arabidopsis’) (MacAlister and Bergmann, 2011); class III HD-ZIP transcription factors play a conserved role in the regulation of leaf polarity in *P. patens* and Arabidopsis but gene family members have acquired regulatory activity in meristems of angiosperms (Yip et al., 2016); and the gene regulatory network that produces rhizoids on the gametophytes of both the moss *P. patens* and the liverwort *Marchantia polymorpha* has been co-opted to regulate root hair formation in Arabidopsis sporophytes (Menand et al., 2007; Pires et al., 2013; Proust et al., 2016). In many cases, however, interpreting the evolutionary trajectory of gene function by comparing lineages as disparate as bryophytes and angiosperms has proved challenging, particularly when only a single representative gene remains in most extant taxa – as is the case for the *LEAFY* (*LFY*) gene family (Himi et al., 2001; Maizel et al., 2005; Sayou et al., 2014).

The LFY transcription factor, which is present across all extant land plant lineages and related streptophyte algae (Sayou et al., 2014), has distinct functional roles in bryophytes and angiosperms. In *P. patens*, LFY regulates cell divisions during sporophyte development (including the first division of the zygote) (Tanahashi et al., 2005), whereas in angiosperms the major role is to promote the transition from inflorescence to floral meristem identity (Carpenter and Coen, 1990; Schultz, 1991; Weigel et al., 1992; Blázquez et al., 1997; Souer et al., 1998; Molinero-Rosales et al., 1999). Given that LFY proteins from liverworts and all vascular plant lineages tested to date (ferns, gymnosperms and angiosperms) bind a conserved target DNA motif, whereas hornwort and moss homologs bind to different lineage-specific motifs (Sayou et al., 2014), the divergent roles in mosses and angiosperms may have arisen through the activation of distinct networks of downstream targets. This suggestion is supported by the observation that PpLFY cannot complement loss-of-function *lfy* mutants in Arabidopsis (Maizel et al., 2005). Similar complementation studies indicate progressive functional changes as vascular plant lineages diverged in that the *lfy* mutant is not complemented by lycophyte LFY proteins (Yang et al., 2017) but is partially and progressively complemented by fern and gymnosperm homologs (Maizel et al., 2005). Because LFY proteins from ferns, gymnosperms and angiosperms recognize the same DNA motif, this progression likely reflects co-option of an ancestral *LFY* gene regulatory network into different developmental contexts. As such, the role in floral meristem identity in angiosperms would have been co-opted from an unknown ancestral context in non-flowering vascular plants, a context that cannot be predicted from existing bryophyte data.

The role of *LFY* in non-flowering vascular plant lineages has thus far been hypothesized on the basis of expression patterns in the lycophyte *Isoetes sinensis* (Yang et al., 2017), several gymnosperm species (Mellerowicz et al., 1998; Mouradov et al., 1998; Shindo et al., 2001; Carlsbecker et al., 2004; Vázquez-Lobo et al., 2007; Carlsbecker et al., 2013) and the fern *Ceratopteris richardii* (hereafter ‘Ceratopteris’) (Himi et al., 2001), which has been used as a model of fern development for a number of years (Hickok et al., 1995). These studies reported broad expression in vegetative and reproductive sporophyte tissues of *I. sinensis* and gymnosperms, and in both gametophytes and sporophytes of Ceratopteris. Although gene expression can be indicative of potential roles in each case, the possible evolutionary trajectories and differing ancestral functions proposed for *LFY* within the vascular plants (Theissen and Melzer, 2007; Moyroud et al., 2010) cannot be resolved without functional validation. Here we present a functional analysis in Ceratopteris that reveals a stem cell maintenance role for at least one of the two *LFY* homologs in both gametophyte and sporophyte shoots and discuss how that role informs our mechanistic understanding of developmental innovations during land plant evolution.

## Results

### The *CrLFY1* and *CrLFY2* genes duplicated recently within the fern lineage

**Figure 1.**
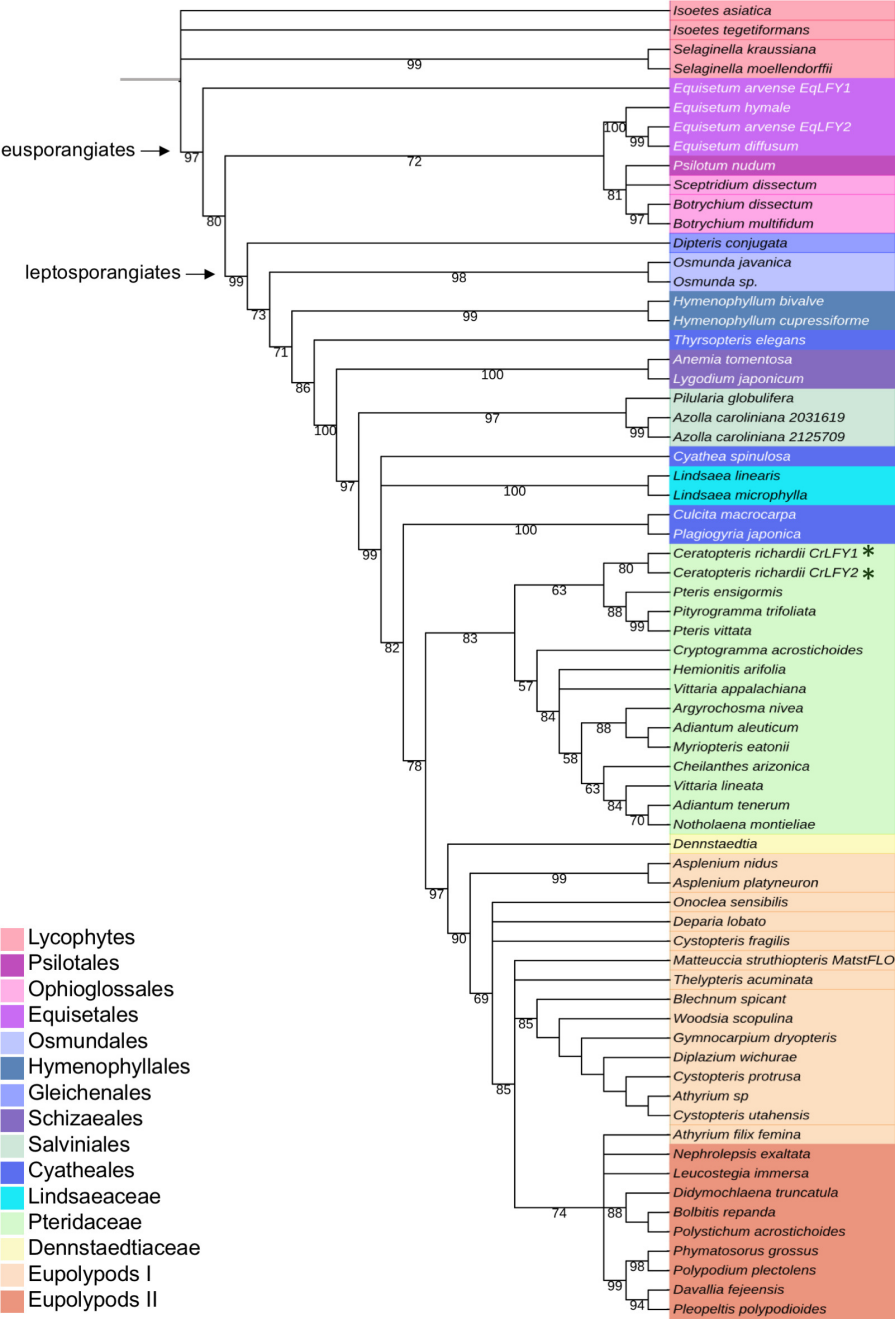
*CrLFY1* and *CrLFY2* arose from a recent gene duplication event. Inferred phylogenetic tree from maximum likelihood analysis of 64 LFY amino acid sequences (see Supplementary file 1 for accession numbers) sampled from within the fern lineage plus lycophyte sequences as an outgroup. Bootstrap values are given for each node. The tree shown is extracted from a phylogeny with representative sequences from all land plant lineages (Figure 1—figure supplement 1). The *Ceratopteris richardii* genome contains no more than two copies of LFY (Figure 1—figure supplement 2; indicated by *). Different taxonomic clades within the fern lineage are denoted by different colours, as shown. The divergence between eusporangiate and leptosporangiate ferns is indicated by arrows.

The *LFY* gene family is present as a single gene copy in most land plant genomes (Sayou et al., 2014). In this regard, the presence of two *LFY* genes in Ceratopteris (Himi et al., 2001) is atypical. To determine whether this gene duplication is more broadly represented within the ferns and related species (hereafter ‘ferns’), a previous amino acid alignment of LFY orthologs (Sayou et al., 2014) was pruned and supplemented with newly-available fern homologs (see Materials and methods) to create a dataset of 120 sequences,~50% of which were from the fern lineage (Supplementary file 1–3). The phylogenetic topology inferred within the vascular plants using the entire dataset (Figure 1—figure supplement 1) was consistent with previous analyses (Qiu et al., 2006; Wickett et al., 2014). Within the ferns (64 in total), phylogenetic relationships between *LFY* sequences indicated that the two gene copies identified in *Equisetum arvense*, *Azolla caroliniana* and Ceratopteris each resulted from recent independent duplication events (Figure 1). Gel blot analysis confirmed the presence of no more than two *LFY* genes in the Ceratopteris genome (Figure 1—figure supplement 2). Given that the topology of the tree excludes the possibility of a gene duplication prior to diversification of the ferns, *CrLFY1* and *CrLFY2* are equally orthologous to the single copy *LFY* representatives in other fern species.

**Figure 1—figure supplement 1.**
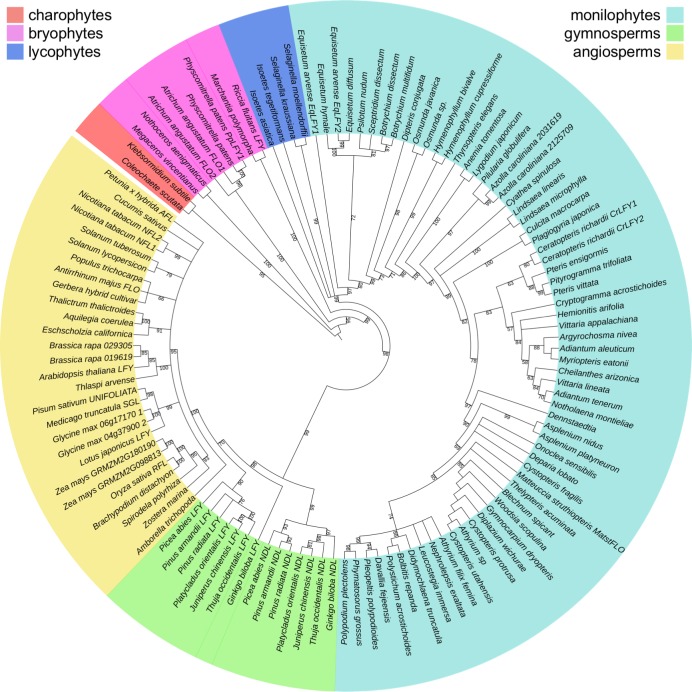
Phylogenetic relationships between *LEAFY* sequences reflect established relationships within vascular plant lineages. Inferred phylogenetic tree from maximum likelihood analysis of 120 LFY sequences sampled from across extant land plant lineages (liverworts, mosses, hornworts, lycophytes, monilophytes i.e. ferns and allies, gymnosperms, angiosperms) including algal (charophyte) sequences as an outgroup. Bootstrap values are given for each node. Sequences belonging to each lineage are denoted by different colours, as shown. The higher-order topology between vascular plant lineages (lycophytes, monilophytes, gymnosperms and angiosperms) is consistent with expected relationships; a gene duplication event resulting in *LFY* and *NEEDLY* clades in gymnosperms has been identified previously (Sayou et al., 2014); and relationships between bryophyte lineages are consistent with differences in the LFY DNA binding site preference, where hornworts and mosses each differ from the preferred site shared by liverworts and vascular plants (Sayou et al., 2014).

**Figure 1—figure supplement 2.**
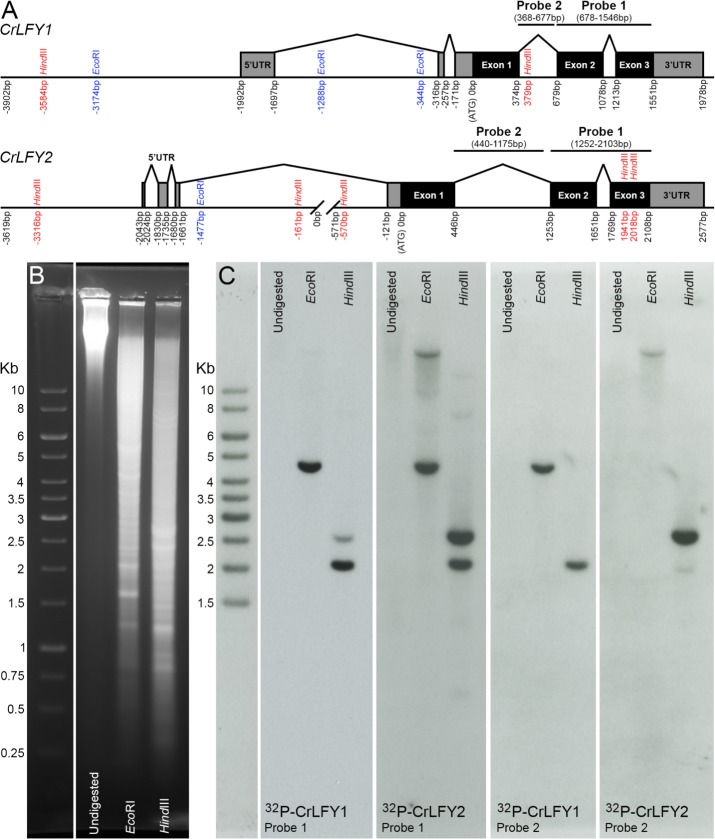
The *Ceratopteris* genome contains only two copies of *LFY*. (**A**) Deduced gene structure of *CrLFY1* and *CrLFY2* loci. All positions marked are given relative to the ATG start codon. Hybridization probes used in DNA gel blot analysis and relevant restriction sites (*Eco*RI, *Hind*III) are marked. *CrLFY1* probe 1 (868bp) and *CrLFY2* probe 1 (851bp) share 79% sequence similarity and hybridize to exons 2 + 3 of each gene (comprising the conserved LFY DNA binding domain). As such, both probes should hybridize to all members of the *LFY* gene family. *CrLFY1* probe 2 (309bp) and *CrLFY2* probe 2 (735bp) hybridize to intron 1 of each gene copy and share no significant sequence similarity. As such, each probe is expected to hybridize to the specific gene copy. (**B, C**) Gel blot analysis of wild-type genomic DNA, digested with *Eco*RI or *Hin*dIII, electrophoresed on an ethidium bromide stained gel (**B**), blotted to nylon membrane and hybridized against different probes (**C**) as described in (**A**). *Eco*RI digestion was predicted to generate single hybridizing fragments for both *CrLFY1* and *CrLFY2*, each spanning both probes with minimum expected fragment sizes of ~2.0 kb and ~3.1 kb, respectively. *Hind*III digestion was predicted to generate a single *CrLFY1* hybridizing fragment recognized by both probes with a minimum size of ~1.6 kb. *Hind*III digestion was predicted to generate a *CrLFY2* fragment of ~2.5 kb hybridizing to probes 1 and 2, a separate fragment with a minimum size of 559 bp overlapped by 85 bp of probe 1 (and so potentially undetectable) plus an undetectable fragment of 11 bp. The hybridization patterns observed (**C**) are consistent with these predictions, with the exon probes cross-hybridizing to predicted fragments of both gene copies (but not to any additional gene fragments) and the intron probes primarily hybridizing to the respective specific gene copy.

### *CrLFY1* and *CrLFY2* transcripts accumulate differentially during the Ceratopteris lifecycle

**Figure 2.**
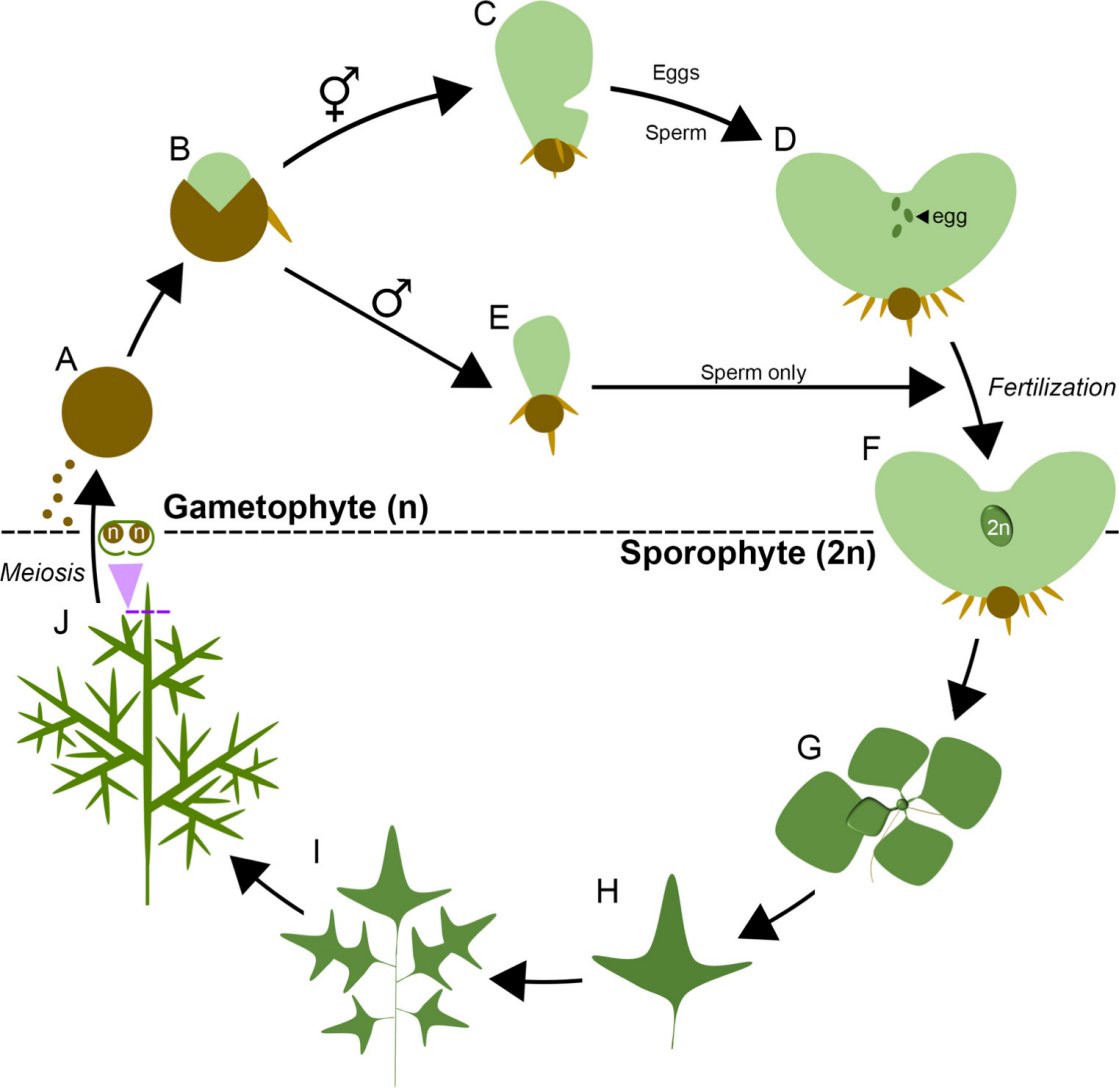
The lifecycle of *Ceratopteris richardii*. Ceratopteris propagates in the haploid gametophyte phase of its lifecycle (**n**) through single-celled spores (**A**) On spore germination (**B**) a two-dimensional photosynthetic thallus develops into one of two sexes, a default hermaphrodite (**C**) which produces eggs and sperm (**D**) or a hormone-induced male that produces sperm only (**E**). Eggs are retained on the hermaphrodite thallus, and fertilization results in the development of a diploid (2n) embryo on the gametophyte (**F**), initiating the sporophyte phase of the lifecycle. The sporophyte establishes a vegetative shoot that initiates leaflike lateral organs (fronds) and roots from its apex (**G**). The first fronds produced are simple but later fronds become increasingly lobed and dissected (**H, I**). The sporophyte undergoes a reproductive phase-change and subsequent fronds generate haploid spores by meiosis on their undersides (**J**), enclosed in a morphologically-distinct curled lamina. Mature spores are dispersed to restart the lifecycle.

The presence of two *LFY* genes in the Ceratopteris genome raises the possibility that gene activity was neo- or sub-functionalized following duplication. To test this hypothesis, transcript accumulation patterns of *CrLFY1* and *CrLFY2* were investigated throughout the Ceratopteris lifecycle (shown as a schematic in Figure 2 for reference).

**Figure 3.**
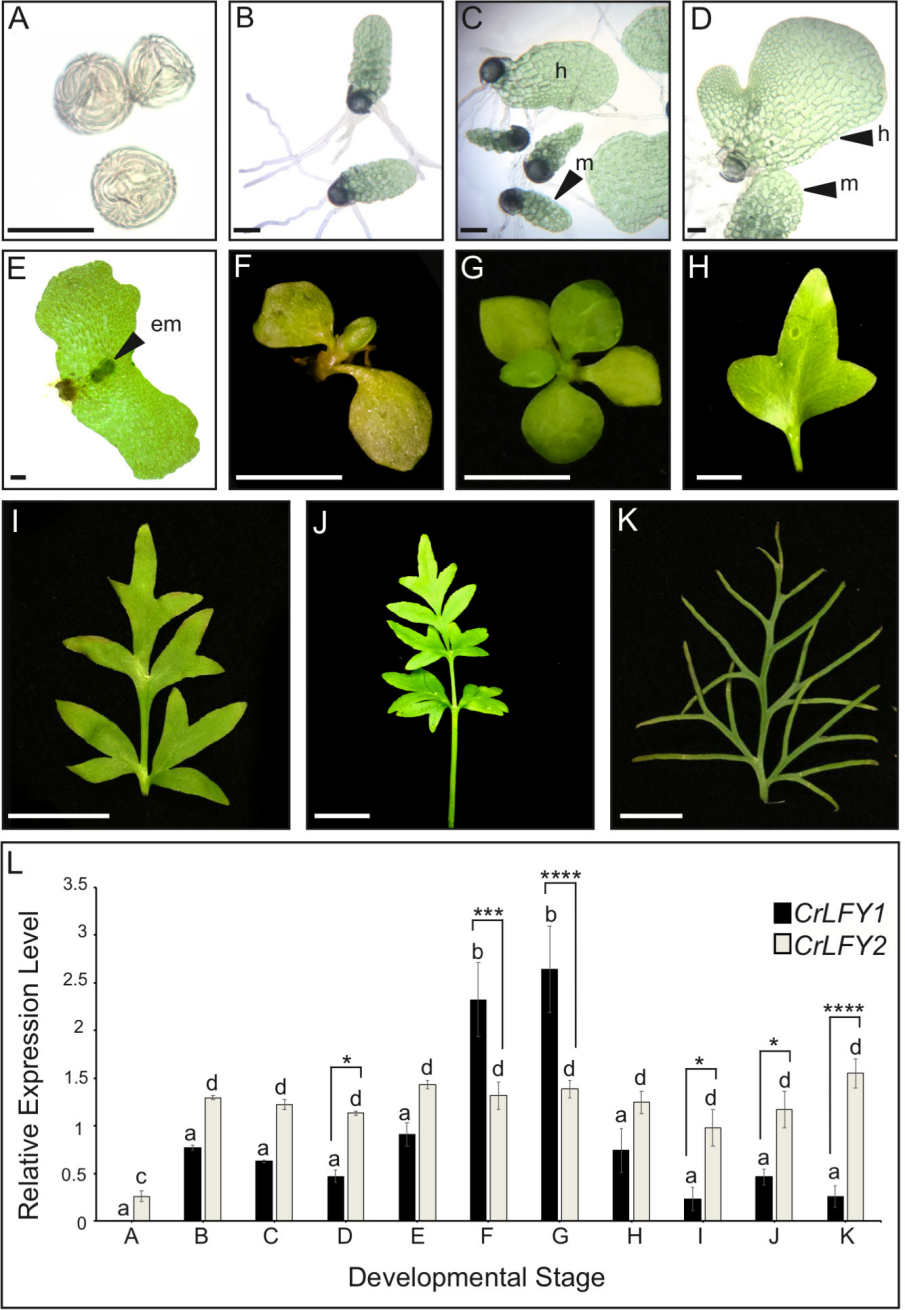
*CrLFY1* and *CrLFY2* are differentially expressed during the Ceratopteris lifecycle. (**A-K**) Representative images of the developmental stages sampled for expression analysis in (**L**). Imbibed spores (**A**); populations of developing gametophytes harvested at 5 (**B, C**) and 8 (**D**) days after spore-sowing (DPS), comprising only males (**B**) or a mixture of hermaphrodites (**h**) and males (**m**) (**C, D**); fertilized gametophyte subtending a developing sporophyte embryo (em) (**E**); whole sporophyte shoots comprising the shoot apex with 3 (**F**) or five expanded entire fronds attached (**G**); individual vegetative fronds demonstrating a heteroblastic progression in which frond complexity increases through successive iterations of lateral outgrowths (pinnae) (**H–J**); complex fertile frond with sporangia on the underside of individual pinnae (**K**). Scale bars = 100 um (**A–E**), 5 mm (**F–H**), 20 mm (**I–K**). (**L**) Relative expression levels of *CrLFY1* and *CrLFY2* (normalized against the housekeeping genes *CrACTIN1* and *CrTBP*) at different stages of development. *n* = 3; Error bars = standard error of the mean (SEM). Pairwise statistical comparisons (ANOVA followed by Tukey’s multiple comparisons test– Supplementary file 4) found no significant difference in *CrLFY2* transcript levels between any gametophyte or sporophyte tissues sampled after spore germination (p>0.05) and no significant difference between *CrLFY1* and *CrLFY2* transcript levels during early gametophyte development (p>0.05) (**B, C**). Differences between *CrLFY1* and *CrLFY2* transcript levels were significant in gametophytes at 8 DPS (p<0.05) (**D**). *CrLFY1* transcript levels were significantly higher in whole young sporophytes (**F**) and vegetative shoots (**G**) compared to isolated fronds (**H–K**) (p<0.05). *CrLFY1* transcript levels in whole sporophytes and shoots were greater than *CrLFY2*, whereas in isolated fronds *CrLFY1* transcript levels were consistently lower than *CrLFY2* (p<0.05). Asterisks denote significant difference (*, p<0.05; **, p<0.01, ***, p<0.001; ****, p<0.0001) between *CrLFY1* and *CrLFY2* transcript levels (Sidak’s multiple comparisons test) within a developmental stage. Letters denote significant difference (p<0.05) between developmental stages for *CrLFY1* or *CrLFY2* (Tukey’s test). Groups marked with the same letter are not significantly different from each other (p>0.05). Statistical comparisons between developmental stages were considered separately for *CrLFY1* and *CrLFY2*. The use of different letters between *CrLFY1* and *CrLFY2* does not indicate a significant difference. 10.7554/eLife.39625.008Figure 3—source data 1.CrLFY qRT-PCR ontogenic expression data

The developmental stages sampled spanned from imbibed spores prior to germination of the haploid gametophyte (Figure 3A), to differentiated male and hermaphrodite gametophytes (Figure 3B–D), through fertilization and formation of the diploid sporophyte embryo (Figure 3E), to development of the increasingly complex sporophyte body plan (Figure 3F–K). Quantitative real-time PCR (qRT-PCR) analysis detected transcripts of both *CrLFY1* and *CrLFY2* at all stages after spore germination, but only *CrLFY2* transcripts were detected in spores prior to germination (Figure 3L). A two-way ANOVA yielded a highly significant interaction (F(10,22) = 14.21; p<0.0001) between gene copy and developmental stage that had not been reported in earlier studies (Himi et al., 2001), and is indicative of differential gene expression between *CrLFY1* and *CrLFY2* that is dependent on developmental stage. Of particular note were significant differences between *CrLFY1* and *CrLFY2* transcript levels during sporophyte development (Supplementary file 4). Whereas *CrLFY2* transcript levels were similar across sporophyte samples, *CrLFY1* transcript levels were much higher in samples that contained the shoot apex (Figure 3F,G) than in those that contained just fronds (Figure 3H–K). These data suggest that *CrLFY1* and *CrLFY2* genes may play divergent roles during sporophyte development, with *CrLFY1* acting primarily in the shoot apex and *CrLFY2* acting more generally.

### Spatial expression patterns of *CrLFY1* are consistent with a retained ancestral role to facilitate cell divisions during embryogenesis

**Figure 4.**
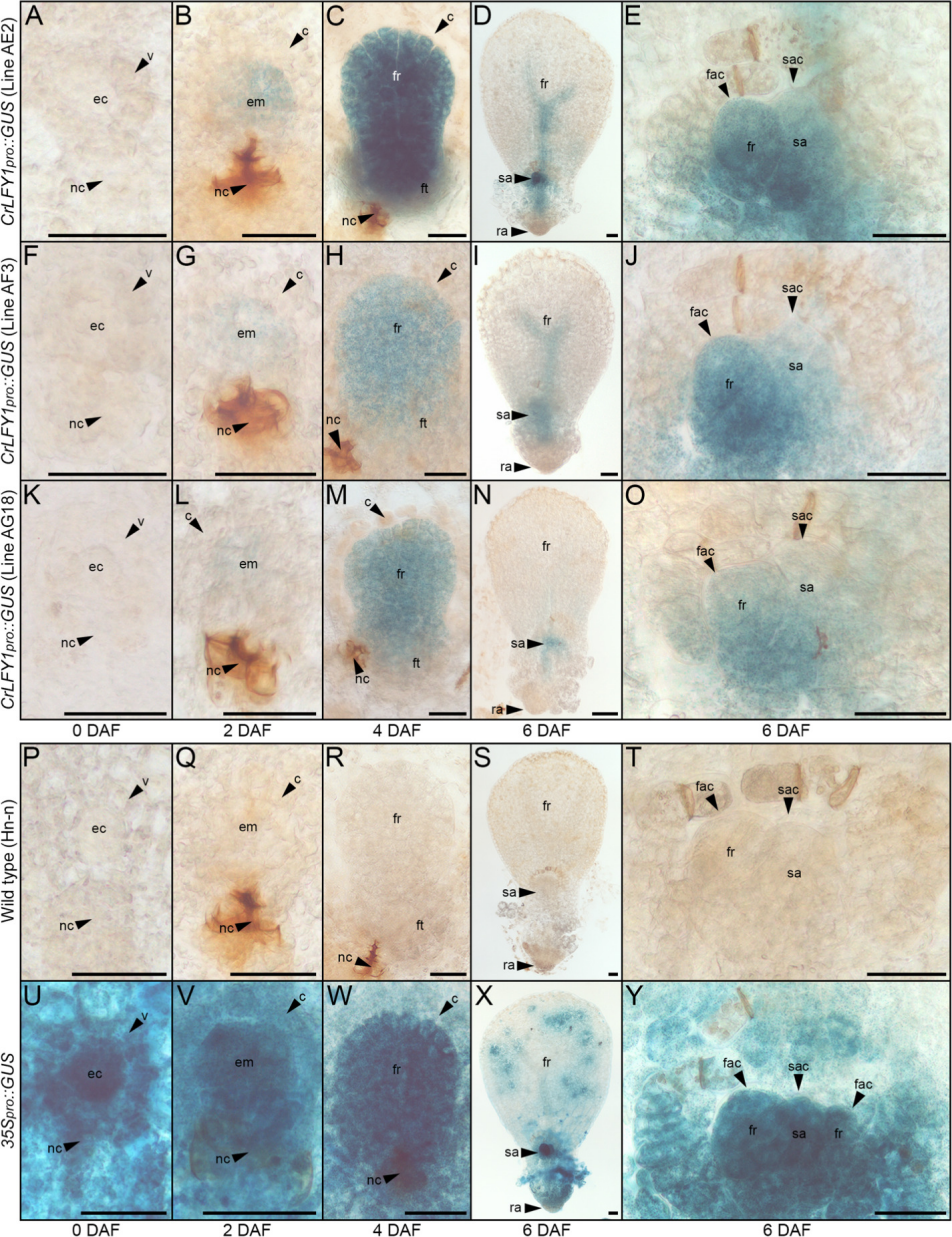
The *CrLFY1* promoter drives reporter gene expression in proliferating tissues of the developing Ceratopteris embryo. (**A–Y**) GUS activity detected as blue staining in developing embryos of three independent *CrLFY1_pro_::GUS* transgenic reporter lines (**A–O**), a representative negative wild-type control line (**P–T**) and a representative positive *35S_pro_::GUS* control line (**U–Y**). Tissues are shown prior to fertilization (**A, F, K, P, U**), or 2 (**B, G, L, Q, V**), 4 (**C, H, M, R, W**), and 6 (**D, I, N, S, X**) days after fertilization (DAF). In *CrLFY1_pro_::GUS* lines, GUS activity first became visible within the first few divisions of embryo development (but not in surrounding gametophyte tissues) at 2 DAF (**B, G, L**) and was expressed in cells of the embryo frond as it proliferated (**C, H, M**). GUS activity was visible in the shoot apex and in frond vascular tissue at 6 DAF (**D, I, N**), with staining in the shoot apical cell (sac), subtending shoot apex tissues and newly-initiated fronds, including the frond apical cell (fac) (**E, J, O**). No GUS activity was detected in wild-type samples (**P–T**), whereas the majority of cells in the constitutively expressing *35S_pro_::GUS* samples stained blue (**U–Y**). Embryos develop on the surface of the gametophyte thallus when an egg cell (ec) within the archegonium (which comprises a venter (**v**) and neck cells (nc) to allow sperm entry) are fertilized. After fertilization, the venter forms a jacket of haploid cells known as the calyptra (**c**) that surrounds the diploid embryo (em). Cell fates in the embryo (embryo frond (fr), embryo foot (ft), root apex (ra) and shoot apex (sa)) are established at the eight-celled stage (Johnson and Renzaglia, 2008), which is around 2 DAF under our growth conditions. Embryogenesis is complete at 6 DAF, after which fronds arise from the shoot apex. Scale bars = 50 μm.

Functional characterization in *P. patens* previously demonstrated that PpLFY promotes cell divisions during early sporophyte development (Tanahashi et al., 2005). To determine whether the spatial domains of *CrLFY1* expression are consistent with a similar role in Ceratopteris embryo (early sporophyte) development, transgenic lines were generated that expressed the reporter gene B-glucuronidase (GUS) driven by a 3.9 kb fragment of the *CrLFY1* promoter (*CrLFY1_pro_::GUS*). This promoter fragment comprised genomic sequence encoding the entire published 5’UTR (Himi et al., 2001) plus a further 1910 bp upstream of the predicted transcription start site (Figure 1—figure supplement 2A). In the absence of a genome sequence, repeated attempts to isolate an analogous fragment of *CrLFY2* sequence were unsuccessful (see Materials and methods for details). Construct maps plus DNA blot and PCR validation of transgenic lines are shown in Figure 4—figure supplements 1–4. GUS activity was monitored in individuals from three independent transgenic lines, sampling both before and up to six days after fertilization (Figure 4A–O), using wild-type individuals as negative controls (Figure 4P–T) and individuals from a transgenic line expressing GUS driven by the constitutive 35S promoter (*35S_pro_*) as positive controls (Figure 4U–Y). Notably, no GUS activity was detected in unfertilized archegonia of *CrLFY1_pro_::GUS* gametophytes (Figure 4A,F,K) but by two days after fertilization (DAF) GUS activity was detected in most cells of the early sporophyte embryo (Figure 4B,G,L). At 4 DAF, activity was similarly detected in all visible embryo cells, including the embryonic frond, but not in the surrounding gametophytic tissue (the calyptra) (Figure 4C,H,M). This embryo-wide pattern of GUS activity became restricted in the final stages of development such that by the end of embryogenesis (6 DAF) GUS activity was predominantly localized in the newly-initiated shoot apex (Figure 4D,E,I,J,N,O). Collectively, the GUS activity profiles indicate that *CrLFY1* expression is induced following formation of the zygote, sustained in cells of the embryo that are actively dividing, and then restricted to the shoot apex at embryo maturity. This profile is consistent with the suggestion that *CrLFY1* has retained the LFY role first identified in *P. patens* (Tanahashi et al., 2005), namely to promote the development of a multicellular sporophyte, in part by facilitating the first cell division of the zygote.

**Figure 4—figure supplement 1.**
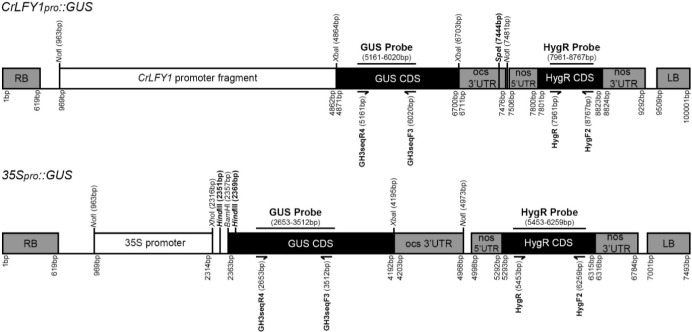
Schematic of *CrLFY1pro::GUS* and *35S_pro_::GUS* constructs. Hybridization probes for the GUS and hygromycin resistance (HygR) genes, plus the position of the *Spe*I and *Hind*III restriction sites used for DNA gel blot analysis (Figure 4—figure supplement 2) are indicated. Restriction sites used in plasmid construction (see Materials and methods) are also indicated. All nucleotide positions are given relative to the start of the T-DNA right border (RB). Primer sequences are listed in the Key Resources Table.

**Figure 4—figure supplement 2.**
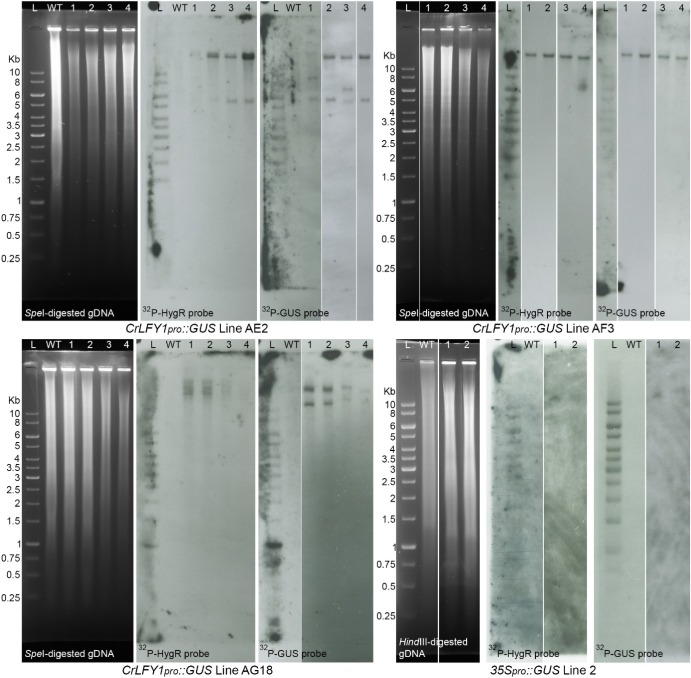
DNA gel blot analysis of *CrLFY1_pro_::GUS* and *35S_pro_::GUS* transgenic lines. *CrLFY1_pro_::GUS* lines AE2, AF3, and AG18 were regenerated from three separate bombardments and so are necessarily independent of one another. Genomic DNA was extracted from four T_1_ sporophytes (arising from the free fertilization of T_1_ gametophytes) within each line, digested with *SpeI* and separated on an electrophoresis gel. Genomic DNA from two T_1_ sporophytes of *35S_pro_::GUS* line two was digested with *Hind*III. Gel blot analysis of both constructs was performed with the same two probes, hybridizing to the GUS CDS and hygromycin resistance (HygR) CDS, respectively (Figure 4—figure supplement 1). For each full-length *CrLFY1_pro_::GUS* T-DNA insertion present in the genome a single insert is predicted to result in unlinked hybridization fragments with minimum sizes of ~2.5 kb (HygR) and ~6 kb (GUS). For *35S_pro_::GUS*, a single fragment with a minimum size of ~5.1 kb is predicted per insertion event for both probes (see Figure 4—figure supplement 1). Gel blot results indicate the presence of one full-length T-DNA insertion in *35Spro::GUS* line 2. Based on the hybridization fragments obtained, *CrLFY1_pro_::GUS* line AE2 contains two potentially full-length insertions of the *CrLFY1pro::GUS* cassette and 1–2 insertions of partial fragments, AF3 contains a single potential full length insertion and AG18 contains two potential full length insertions.

**Figure 4—figure supplement 3.**
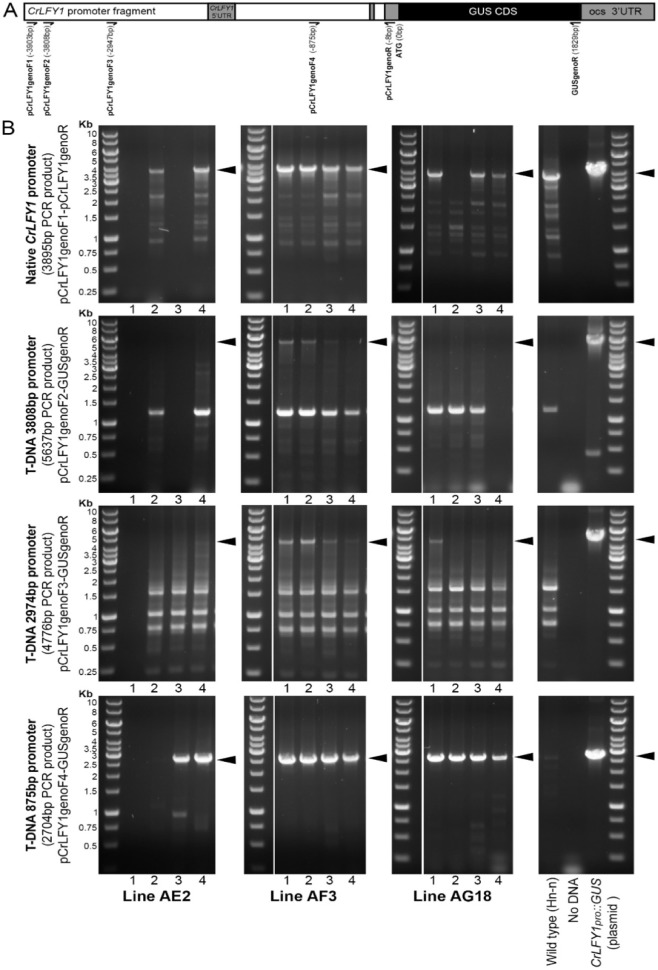
PCR analysis of *CrLFY1_pro_::GUS* T_1_ lines identified full-length or near full-length *CrLFY1* promoter sequences in T-DNA insertions. (**A**) Schematic of the *CrLFY1_pro_::GUS* construct marking binding sites of PCR primers used in (**B**). All positions are given relative to the GUS ATG. Primer sequences are listed in the Key Resources Table. (**B**) PCR was performed on genomic DNA from the same four individual T_1_ sporophytes within each line investigated by gel blot analysis (see Figure 4—figure supplement 2). PCR reactions were performed to amplify the native 3.9 kb *CrLFY1* promoter as a positive control (row 1) and to amplify T-DNA specific products (rows 2–4) containing the GUS CDS and different lengths of contiguous *CrLFY1* promoter sequence (see **A**). Black arrowheads mark the expected size of the target PCR product in each reaction. PCR analysis identified at least one full-length GUS CDS with ~3.8 kb of *CrLFY1* promoter in line AF3 (row 2). Faint PCR products indicate that line AG18 and AE2 probably contain a full-length GUS CDS plus ~3 kb of *CrLFY1* promoter sequence (row 3). All lines carry a GUS CDS plus a minimum *CrLFY1* promoter length of 875 bp (row 4).

**Figure 4—figure supplement 4.**
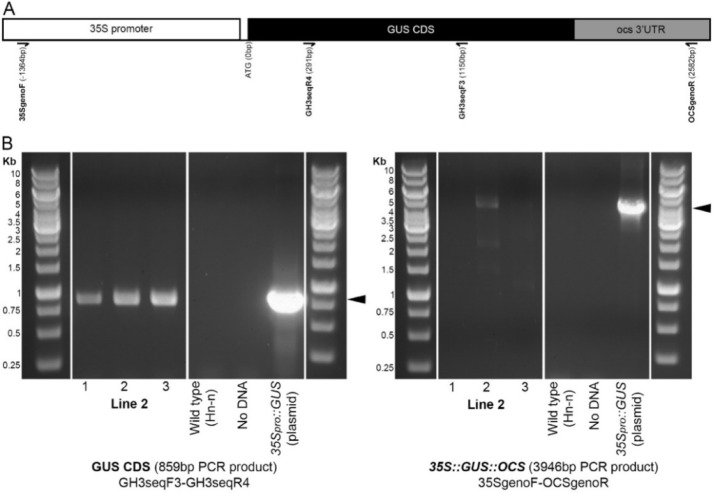
PCR analysis of *35S_pro_::GUS* positive control line identified a full-length *35S_pro_::GUS* insertion. (**A**) Schematic of the *35S_pro_::GUS* construct marking binding sites of genotyping primers used in (**B**). All positions shown are relative to the GUS ATG. Primer sequences are listed in the Key Resoucres Table. (**B**) PCR was performed on genomic DNA extracted from three T_1_ individuals, including the two investigated by gel blot analysis (Figure 4—figure supplement 2). T-DNA specific reactions were performed to amplify the GUS CDS (left) previously identified by gel blot analysis (Figure 4—figure supplement 2) using the same primers as the gel-blot probe, and a PCR product spanning almost the full length of the *35S::GUS::OCS* construct (right). Black arrowheads mark the expected size of the target PCR product in each reaction. PCR results indicate the presence of a full length *35S_pro_::GUS* T-DNA in this line.

### *CrLFY1* is expressed in dividing tissues throughout shoot development

**Figure 5.**
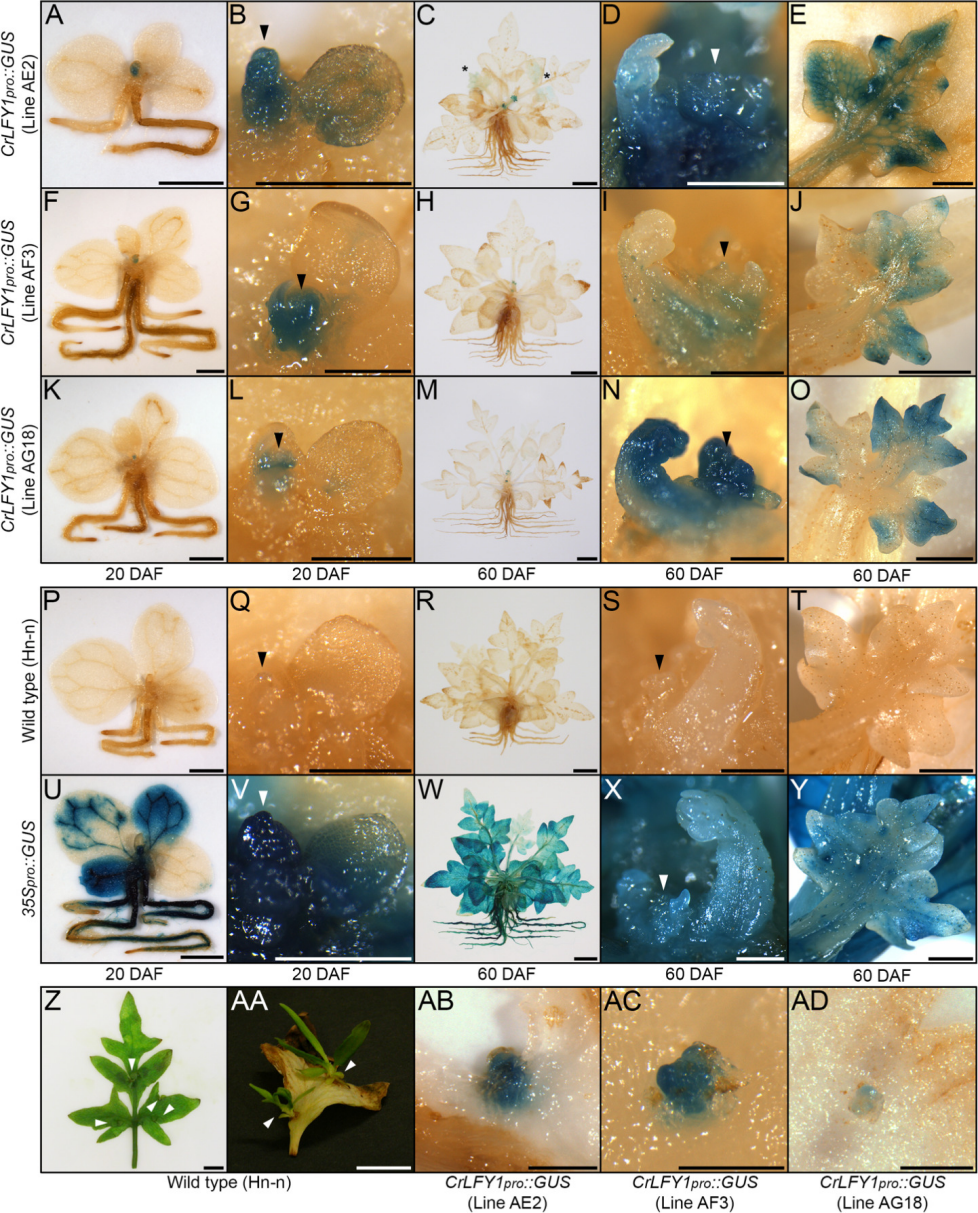
The *CrLFY1* promoter drives reporter gene expression in proliferating shoot tissues of the Ceratopteris sporophyte. (**A–Y**) GUS activity detected as blue staining in post-embryonic sporophytes from three independent *CrLFY1_pro_::GUS* transgenic reporter lines (**A–O**), negative wild-type controls (**P–T**) and positive *35S_pro_::GUS* controls (**U–Y**). Sporophytes were examined at 20 DAF (**A, B, F, G, K, L, P, Q, U, V**) and 60 DAF (**C–E, H–J, M–O, R–T, W–Y**). GUS staining patterns are shown for whole sporophytes (**A, C, F, H, K, M, P, R, U, W**), shoot apices (arrowheads) (**B, D, G, I, L, N, Q, S, V, X**) and developing fronds (**E, J, O, T, Y**). In *CrLFY1_pro_::GUS* sporophytes at 20 DAF (producing simple, spade-like fronds) GUS activity was restricted to the shoot apex (**A, F, K**) and newly-initiated frond primordia, with very low activity in expanded fronds (**B, G, L**). In *CrLFY1_pro_::GUS* sporophytes at 60 DAF (producing complex, highly dissected fronds) GUS activity was similarly seen in the apex (**C, H, M**), but persisted for longer during frond development. Activity was initially detected throughout the frond primordium (**D, I, N**), before becoming restricted to actively proliferating areas of the lamina (**E, J, O**). Scale bars = 2 mm (**A, F, K, P, U**), 500 μm (**B, D, G, I, L, N, Q, S, V, X**) 10 mm (**C, H, M, R, W**), 1 mm (**E, J, O, T, Y**). *=GUS staining in maturing frond. GUS staining patterns were the same in leaves formed after the reproductive transition (Figure 5—figure supplement 1). (**Z-AD**) Fronds can initiate *de novo* shoots (white arrowheads) from marginal tissue between existing frond pinnae (**Z, AA**). GUS activity was detected in emerging de novo shoot apices on *CrLFY1_pro_::GUS* fronds (**AB–AD**). Scale bars = 10 mm (**Z, AA**), 500 μm (**AB–AD**).

Both mosses and ferns form embryos, but moss sporophyte development is determinate post-embryogenesis (Kato and Akiyama, 2005; Kofuji and Hasebe, 2014) whereas fern sporophytes are elaborated post-embryonically from indeterminate shoot apices (Bierhorst, 1977; White and Turner, 1995). The Ceratopteris shoot apex comprises a single apical cell that generates daughter cells through asymmetric divisions, and individual lateral organs (fronds and root) arise from their own apical cells specified within the grouped descendants of these daughter cells (Hou and Hill, 2002; Hou and Hill, 2004). *CrLFY1_pro_::GUS* expression in the shoot apex at the end of embryogenesis (Figure 4E,J,O) and elevated transcript levels in shoot apex-containing sporophyte tissues (Figure 3L) suggested an additional role for *CrLFY1* relative to that seen in mosses, namely to promote proliferation in the indeterminate shoot apex. To monitor *CrLFY1* expression patterns in post-embryonic sporophytes, GUS activity was assessed in *CrLFY1_pro_::GUS* lines at two stages of vegetative development (Figure 5A–O) and after the transition to reproductive frond formation (Figure 5—figure supplement 1A–L). Wild-type individuals were used as negative controls (Figure 5P–T; Figure 5—figure supplement 1M–P) and *35S_pro_::GUS* individuals as positive controls (Figure 5U–Y; Figure 5—figure supplement 1Q–T). In young sporophytes (20 DAF), GUS activity was primarily localized in shoot apical tissues and newly-emerging frond primordia (Figure 5A,F,K), with very little activity detected in the expanded simple fronds produced at this age (Figure 5B,G,L). In older vegetative sporophytes (60 DAF), which develop complex dissected fronds (Figure 5C,H,M), GUS activity was similarly localized in the shoot apex and young frond primordia in two out of the three fully characterized lines (Figure 5D,I,N) and in a total of 8 out of 11 lines screened (from seven independent rounds of plant transformation). GUS activity was also detected in developing fronds in regions where the lamina was dividing to generate pinnae and pinnules (Figure 5E,J,O). In some individuals GUS activity could be detected in frond tissues almost until maturity (Figure 5C). Notably, patterns of *CrLFY1_pro_::GUS* expression were the same in the apex and complex fronds of shoots before (60 DAF) (Figure 5C–E,H–J,M–O) and after (~115 DAF) the reproductive transition (Figure 5—figure supplement 1A–L). Consistent with a general role for *CrLFY1* in promoting cell proliferation in the shoot, GUS activity was also detected in shoot apices that initiate *de novo* at the lamina margin between pinnae (Figure 5Z–AD). Together these data support the hypothesis that *LFY* function was recruited to regulate cell division processes in the shoot when sporophytes evolved from determinate to indeterminate structures.

**Figure 5—figure supplement 1.**
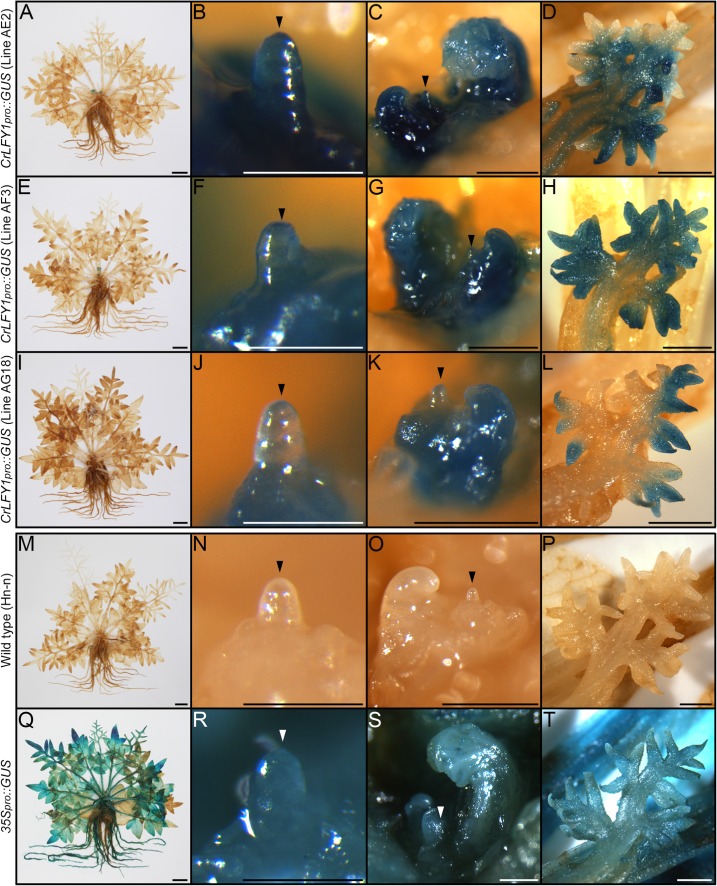
*CrLFY1_pro_::GUS* expression patterns are similar in Ceratopteris shoots before and after reproductive phase change. GUS activity detected as blue staining in sporophytes producing fronds with spore-bearing morphology (narrowing and elongation of pinnae) from three independent *CrLFY1_pro_::GUS* transgenic reporter lines (**A–L**); 110–124 DAF), negative wild-type controls (**M–P**); 113 DAF) and positive *35S_pro_::GUS* controls (**Q–T**); 110 DAF). Staining patterns were consistent between the three independent *CrLFY1_pro_::GUS* transgenic lines (**A, E, I**), and were similar to those seen at 60 DAF (Figure 4C, (H,M). GUS activity was observed throughout the shoot apex (**B, F, J**) and in recently-emerged frond primordia (**C, G, K**). Activity persisted later in frond development, becoming restricted to developing pinnae (**D, H, L**). GUS staining was lost from fronds prior to maturity (**A, E, I**). No endogenous GUS activity was detected in wild-type controls (**M–P**) whereas activity was detected throughout all non-senescent tissues in the *35S_pro_::GUS* line (**Q–T**).

### *CrLFY1* regulates activity of the sporophyte shoot apex

To test the functional significance of *CrLFY* expression patterns, transgenic RNAi lines were generated in which one of four RNAi constructs targeted to *CrLFY1, CrLFY2* or both were expressed from the maize ubiquitin promoter (*ZmUbi_pro_*). Construct maps plus DNA blot and PCR validation of transgenic lines are shown in Figure 6—figure supplements 1–5. Genotypic screening identified 10 lines which contained the complete transgene cassette and three lines that contained a fragment of the transgene which included the antibiotic resistance marker but not the RNAi hairpin (Table 1).

**Table 1.** Summary of *CrLFY* RNAi transgenic lines and their phenotypic characterization. Transgenic lines exhibited gametophytic developmental arrest and/or sporophyte shoot termination at varying stages of development. ‘+’ indicates that a particular line was phenotypically normal at the developmental stage indicated, ‘−’ indicates that development had arrested at or prior to this stage. In lines marked ‘+/-’ the stage at which developmental defects occurred varied between individuals within the line, and at least some arrested individuals were identified at the stage indicated. The five *ZmUbi_pro_::CrLFY1/2-i1* lines shown were generated from three rounds of transformation, the pairs of lines B16 and B19 and D2 and D4 potentially arising from the same transformation event. The no hairpin control lines NHC-2 (F3) and NHC-3 (F4) may similarly have arisen from a single transformation event. In all other cases, each transgenic line arose from a separate round of transformation and so must represent independent T-DNA insertions.

RNAi transgene	Line	Transfor-mation replicate	Gametophyte phase				Sporophyte phase				
			Spore germin-ation	AC-based growth	Notch meristem-based growth	% arrested	Embryo	Shoot apex initiated	Simple frond	Complex frond	% arrested
*ZmUBI_pro_::CrLFY1/2-i1*	B16	1	+	-	-	99.86	+	+	-	-	<5%
*ZmUBI_pro_::CrLFY1/2-i1*	B19	1	+	-	-	50.00	+	+	+	-	<5%
*ZmUBI_pro_::CrLFY1/2-i1*	D13	2	+	-	-	99.80	+	+	-	-	<5%
*ZmUBI_pro_::CrLFY1/2-i1*	D2	3	+	+	+	0.00	+	+	-	-	<5%
*ZmUBI_pro_::CrLFY1/2-i1*	D4	3	+	+	+	0.00	+	+	+	+	<5%
*ZmUBI_pro_::CrLFY1/2-i2*	F9	4	+	-	-	0.00	+	+	-	-	<5%
*ZmUBI_pro_::CrLFY1/2-i2*	F14	5	-	-	-	100.00	-	-	-	-	0
*ZmUBI_pro_::CrLFY1-i3*	E8	6	+	+/-	+/-	100.00	-	-	-	-	0
*ZmUBI_pro_::CrLFY1-i3*	G13	7	+	+	+	0.00	+	+	-	-	<5%
*ZmUBI_pro_::CrLFY2-i4*	C3	8	+	+	+	0.00	+	+	-	-	<5%
NHC-1 (control)	D20	9	+	+	+	0.00	+	+	+	+	0
NHC-2 (control)	F3	10	+	+	+	0.00	+	+	+	+	0
NHC-3 (control)	F4	10	+	+	+	0.00	+	+	+	+	0

**Figure 6.**
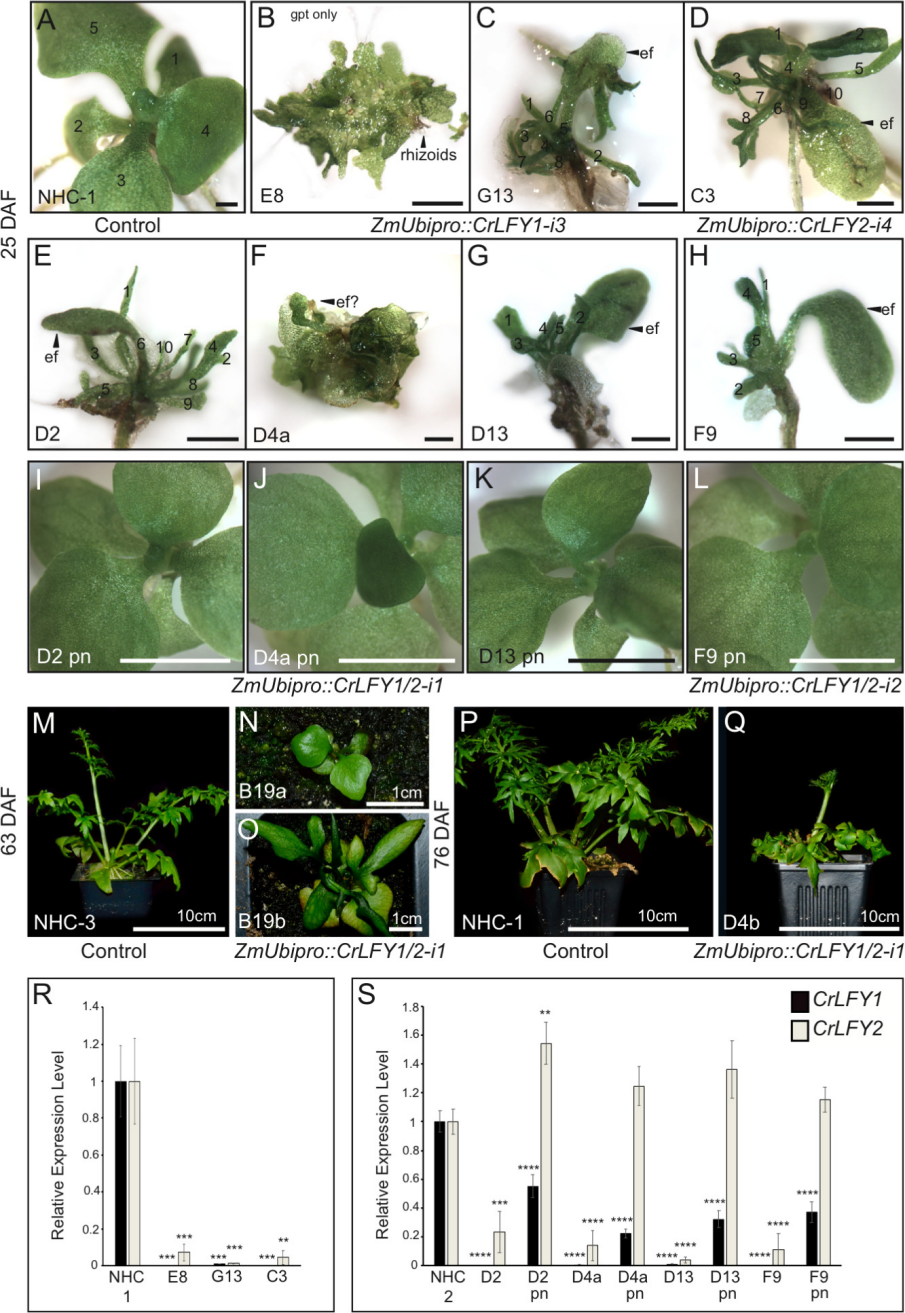
Suppression of *CrLFY* expression causes early termination of the Ceratopteris sporophyte shoot apex. (**A-L**) Sporophyte phenotype 25 days after fertilization (DAF) in no hairpin control, NHC-1 (**A**) and transgenic lines carrying RNAi constructs against *CrLFY1* (*ZmUbi_pro_::CrLFY1-i3*) (**B, C**), *CrLFY2* (*ZmUbi_pro_::CrLFY1-i4*) (**D**) and both *CrLFY1* and *CrLFY2* (*ZmUbi_pro_::CrLFY1/2-i1* and *ZmUbi_pro_::CrLFY1/2-i2*) (**E–L**). In some lines, both aborted and phenotypically normal sporophytes were identified (compare **E** and **I**; **F** and **J**; **G** and **K**; **H** and **L**). The presence of the RNAi transgene in phenotypically normal sporophytes was validated by genotyping (Figure 6—figure supplement 5). Scale bars = 1 mm (**A–H**), 5 mm (**I–L**). (**M–Q**) Sporophyte phenotype of two no hairpin control (NHC-3 and NHC-1) (**M, P**) and two *ZmUbi_pro_::CrLFY1/2-i1* (**N, O**) lines at 63 (**M–O**) and 76 (**P,Q**) DAF. (**R, S**) qRT-PCR analysis of *CrLFY1* and *CrLFY2* transcript levels (normalized against the averaged expression of reference genes *CrACTIN1* and *CrTBP*) in the sporophytes of the RNAi lines shown in (**A–L**). Transcript levels are depicted relative to no hairpin controls (NHC-1or −3), *n* = 3, error bars = standard error of the mean (SEM). *CrLFY1* and *CrLFY2* expression levels were significantly reduced compared to controls (p<0.01 or less) in all transgenic lines where sporophyte shoots undergo early termination (**A–H**), but in phenotypically normal (pn) sporophytes segregating in the same lines (**I–L**), only *CrLFY1* transcript levels were reduced (p<0.0001). *CrLFY2* transcript levels in pn sporophytes were not significantly lower than in controls. Asterisks denote level of significant difference from controls (**p<0.01, ***p<0.001; ****p<0.0001). 10.7554/eLife.39625.023Figure 6—source data 1.CrLFY RNAi lines qRT-PCR expression data

In the no hairpin control (NHC) plants, post-embryonic shoot development initiated with the production of simple, spade-like fronds from the shoot apex (Figure 6A) as in wild type. In eight transgenic lines, sub-populations of sporophytes developed in which this early stage of sporophyte development was perturbed, one line (E8) failing to initiate recognizable embryos (Figure 6B) and the remainder exhibiting premature shoot apex termination, typically after producing several distorted fronds (Figure 6C–H). Sub-populations of phenotypically normal transgenic sporophytes were also identified in some of these lines (Figure 6I–L). The two remaining lines exhibited less severe shoot phenotypes, one undergoing shoot termination after the production of simple (B19a) or lobed (B19b) fronds at the stage when control sporophytes produced complex dissected fronds (Figure 6M–O), and the other (D4b) completing sporophyte development but reduced in size to approximately 50% of controls (Figure 6P,Q). Despite the predicted sequence specificity of *CrLFY1-i3* and *CrLFY2-i4* (Supplementary file 5), qRT-PCR analysis found that all four RNAi constructs led to suppressed transcript levels of both *CrLFY* genes (Figure 6R,S). The severity of the shoot phenotype was correlated with the level of endogenous *CrLFY* transcripts detected across all lines (Figure 6R,S), with relative levels of both *CrLFY1* and *CrLFY2* significantly reduced compared to controls in all early-terminating sporophytes (E8, G13, C3, D2, D4a, D13, F9) (p<0.01 or less). In phenotypically normal transgenic siblings *CrLFY2* transcript levels were not significantly lower than controls (indeed in line D2, levels were higher p<0.01) whereas *CrLFY1* levels were significantly reduced (p<0.0001), as in arrested siblings. Together, these data indicate that *CrLFY2* can compensate for some loss of *CrLFY1*, but at least 22% of *CrLFY1* activity is required for normal development (line D4a pn, Figure 6J,S). It can thus be concluded that *CrLFY1* and *CrLFY2* act partially redundantly to maintain indeterminacy of the shoot apex in Ceratopteris, a role not found in the early divergent bryophyte *P. patens*, nor known to be retained in the majority of later diverging flowering plants.

**Figure 6—figure supplement 1.**
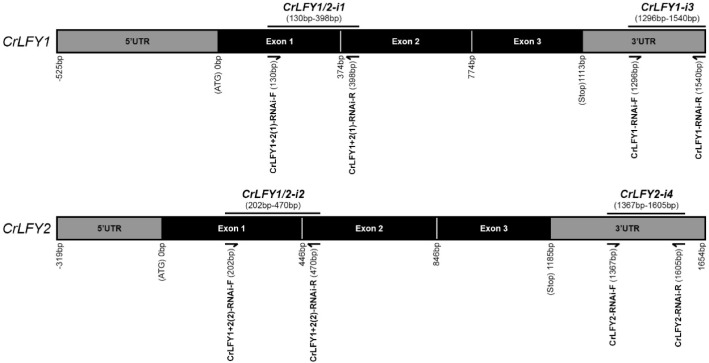
Positions of *CrLFY* RNAi target sequences. Schematic of *CrLFY1* and *CrLFY2* transcripts, showing recognition sequences used in RNAi constructs (black bars). 5’ and 3’ untranslated regions (UTRs) are marked by grey boxes, coding sequence (CDS) by black boxes, with exons as indicated. Positions are given relative to the translational start codon of each transcript. Four RNAi constructs were generated Two of these (*ZmUbi_pro_::CrLFY1/2-i1* and *ZmUbi_pro_::CrLFY1/2-i2*) targeted both *CrLFY1* and *CrLFY2* using conserved coding sequence amplified from *CrLFY1* (*ZmUbi_pro_::CrLFY1/2-i1*) or *CrLFY2* (*ZmUbi_pro_::CrLFY1/2-i2*). The two remaining constructs (*ZmUbi_pro_::CrLFY1-i3* and *ZmUbi_pro_::CrLFY2-i4*) incorporate target sequence amplified from the 3’UTR region of *CrLFY1* and *CrLFY2*, respectively. The position of primers used in target sequence amplification are shown. Primer sequences are supplied in the Key Resources table.

**Figure 6—figure supplement 2.**
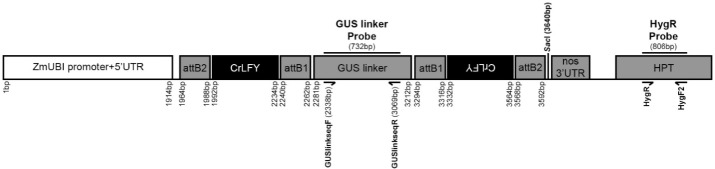
Generalized schematic of *CrLFY* RNAi constructs. Each RNAi construct carries inverted repeats of *CrLFY-*derived sequence (see Figure 6—figure supplement 1 and Supplementary file 5) to generate a hairpin bridged by a linker sequence derived from the GUS CDS (Miki and Shimamoto, 2004). The positions given are relative to the start of the maize ubiquitin promoter (*ZmUbi_pro_*) that is driving RNAi expression, and are shown for *ZmUbi_pro_::CrLFY1-i3*, with the length of the RNAi target sequence varying between the four different constructs (see Figure 6—figure supplement 1). The sites of hybridization against probes for the GUS linker and hygromycin resistance marker (HygR) are shown. The position of a *Sac*I restriction site (used in gel blot analysis, see Figure 6—figure supplement 3) is indicated. No *Sac*I sites are present in any *CrLFY* target sequence used. The primers used in probe amplification are given in the Key Resources Table.

**Figure 6—figure supplement 3.**
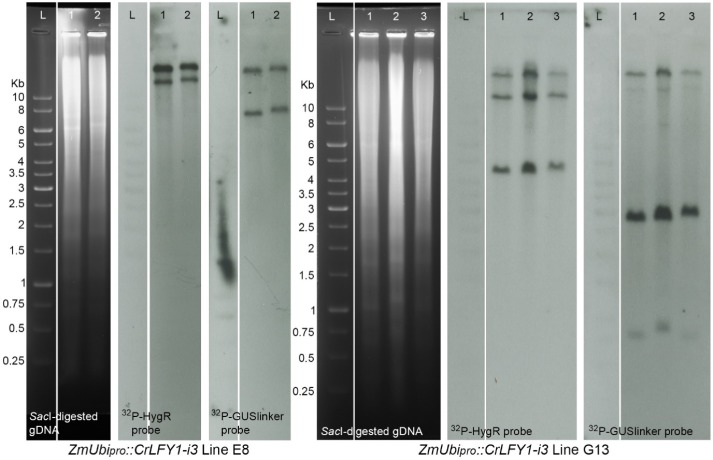
Gel blot analysis of *ZmUbi_pro_::CrLFY1-i3* T_1_ transgenic lines. *ZmUbi_pro_::CrLFY1-i3* lines E8 and G13 were regenerated from two separate bombardments and so are necessarily independent of one another. Genomic DNA was extracted from four T_1_ sporophytes (arising from the free fertilization of T_1_ gametophytes) within each line, digested with *Spe*I and separated on an electrophoresis gel. Gel blot analysis of both constructs was performed with probes hybridizing either to the GUS linker of the RNAi hairpin or to the hygromycin resistance (HygR) CDS (Figure 6—figure supplement 2). For each full-length *ZmUbi_pro_::CrLFY1-i3* T-DNA insertion present in the genome a single insert is predicted to result in unlinked hybridization fragments with minimum sizes of ~1 kb (HygR) or ~3.6 kb (GUS linker). Based on the hybridization fragments obtained, line E8 carries two full-length insertions of the *CrLFY1* RNAi cassette and line G13 carries two full-length insertions plus two additional partial insertions.

**Figure 6—figure supplement 4.**
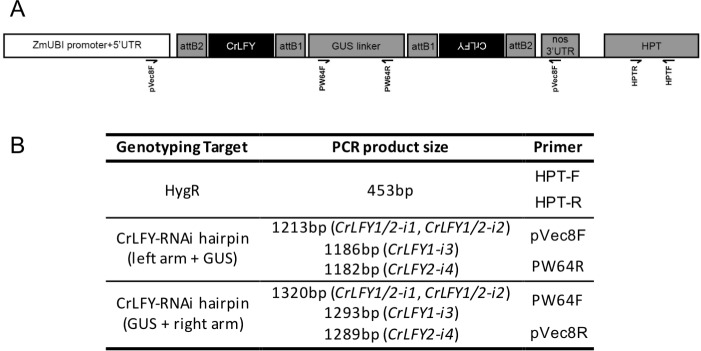
Binding site of *CrLFY* RNAi genotyping PCR primers. (**A**) Generalized schematic of *CrLFY* RNAi T-DNA with the relative position of primers used in genotyping PCR (see Figure 6—figure supplement 5, Figure 7—figure supplement 1) marked. The primer binding sites are common to all four RNAi constructs, with the size of the hairpin-containing PCR products varying due to the insertion of different *CrLFY* target sequences. (**B**) Primer combinations for each genotyping reaction and expected PCR product sizes for each *CrLFY* RNAi construct. Primer sequences are listed in the Key Resources Table.

**Figure 6—figure supplement 5.**
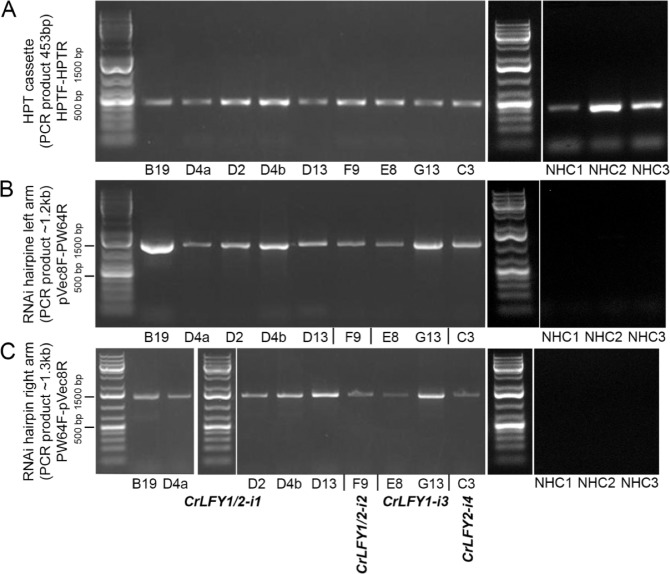
Genotyping PCR confirms the presence of *CrLFY* RNAi T-DNA in transgenic lines and the absence of the RNAi hairpin in no hairpin control lines. PCR was performed on genomic DNA extracted from T_1_ sporophytes pre-selected for antibiotic resistance. The presence of the HPT cassette (**A**) was confirmed in all lines by PCR. The presence or absence of both arms of the RNAi hairpin (**B, C**) were confirmed in each line by PCR, and where sequenced the amplified products were as expected.

### *CrLFY* promotes apical cell divisions in the gametophyte

**Figure 7.**
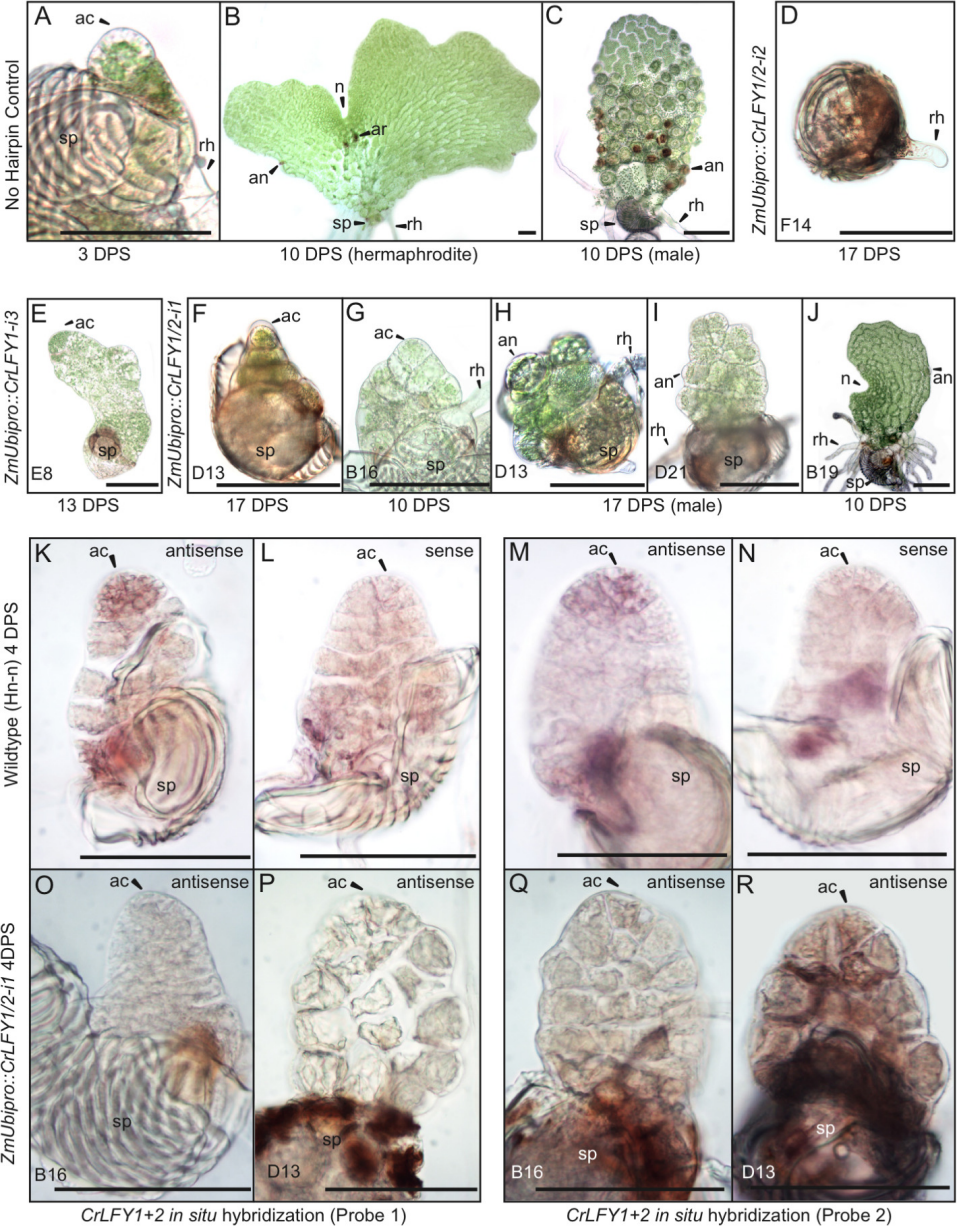
Suppression of *CrLFY* expression causes early termination of the Ceratopteris gametophyte apical cell. (**A–C**) In no hairpin control lines, the gametophyte established a triangular apical cell (ac) shortly after spore (sp) germination (**A**). Divisions of the apical cell established a photosynthetic thallus in both hermaphrodite and male gametophytes. At 10 days post spore sowing (DPS) both gametophyte sexes were approaching maturity, with the hermaphrodite (**B**) having formed a chordate shape from divisions at a lateral notch meristem (**n**) and having produced egg-containing archegonia (ar), sperm-containing antheridia (an), and rhizoids (rh). The male (**C**) had a more uniform shape with antheridia across the surface. These phenotypes were identical to wild-type. (**D–J**) When screened at 10–17 DPS, gametophytes from multiple RNAi lines (as indicated) exhibited developmental arrest, mostly associated with a failure of apical cell activity. Arrest occurred at various stages of development from failure to specify an apical cell, resulting in only a rhizoid being produced and no thallus (**D**) through subsequent thallus proliferation (**E–I**). Gametophyte development in one line progressed to initiation of the notch meristem but overall thallus size was severely reduced compared to wild-type (**J**). (**K–R**) In situ hybridization with antisense probes detected *CrLFY* transcripts in the apical cell and immediate daughter cells of wild-type gametophytes at 4 DPS (**K, M**). No corresponding signal was detected in controls hybridized with sense probes (**L, N**). In the arrested gametophytes of two *ZmUbi_pro_::CrLFY1/2-i1* lines *CrLFY* transcripts could not be detected (**O–R**), and transgene presence was confirmed (Figure 7—figure supplement 1). Scale bars = 100 μm.

In six of the RNAi lines that exhibited sporophyte developmental defects, it was notable that 50–99% of gametophytes arrested development prior to the sporophyte phase of the lifecycle (Table 1). This observation suggested that LFY plays a role in Ceratopteris gametophyte development, a function not previously demonstrated in either bryophytes or angiosperms. During wild-type development, the Ceratopteris gametophyte germinates from a single-celled haploid spore, establishing a single apical cell (AC) within the first few cell divisions (Figure 7A). Divisions of the AC go on to form a two-dimensional photosynthetic thallus in both the hermaphrodite, where a notch meristem takes on growth (Figure 7B), and male sexes (Figure 7C) (Banks, 1999). In contrast, the gametophytes from six RNAi lines (carrying either *ZmUbi_pro_::CrLFY1-i3*, *ZmUbi_pro_::CrLFY1/2-i1* or *ZmUbi_pro_::CrLFY1/2-i2*) exhibited developmental arrest (Figure 7D–J), which in five lines clearly related to a failure of AC activity. The point at which AC arrest occurred varied, in the most severe line occurring prior to or during AC specification (Figure 7D) and in others during AC-driven thallus proliferation (Figure 7E–I). Failure of AC activity was observed in both hermaphrodites (Figure 7E) and males (Figure 7H,I). The phenotypically least-severe line exhibited hermaphrodite developmental arrest only after AC activity had been replaced by the notch meristem (Figure 7J). A role for *CrLFY* in maintenance of gametophyte AC activity was supported by the detection of *CrLFY* transcripts in the AC and immediate daughter cells of wild-type gametophytes by in situ hybridization (Figure 7K–N). By contrast *CrLFY* transcripts were not detected in arrested *ZmUbi_pro_::CrLFY1/2-i1* lines (Figure 7O–R) in which the presence of the transgene was confirmed by genotyping of individual arrested gametophytes (Figure 7—figure supplement 1). *CrLFY1* and *CrLFY2* transcripts could not be clearly distinguished in situ due to sequence similarity (see Supplementary file 6), and hence the observed phenotypes could not be ascribed to a specific gene copy. However, these data support a role for at least one *CrLFY* homolog in AC maintenance during gametophyte development, and thus invoke a role for LFY in the regulation of apical activity in both the sporophyte and gametophyte phases of vascular plant development.

**Figure 7—figure supplement 1.**
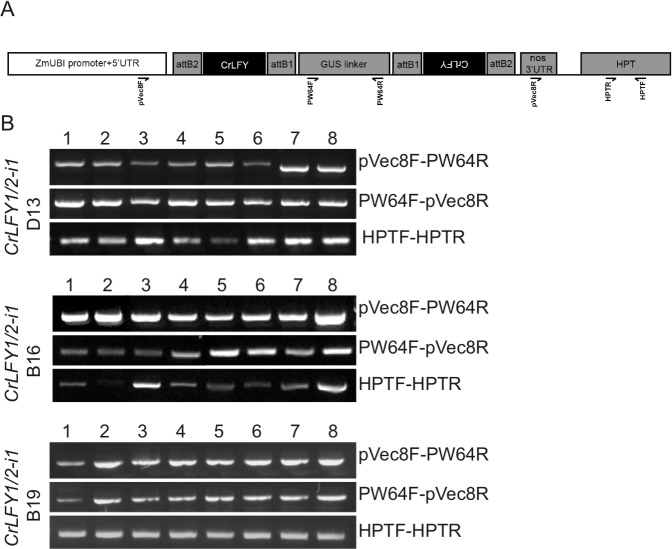
Gametophytes exhibiting developmental arrest were transgenic. **A**. Generalized schematic of *CrLFY* RNAi T-DNA with the relative position of primers used in genotyping PCR marked. Primer sequences and expected PCR product sizes for each *CrLFY* RNAi construct are given in Figure 6—figure supplement 4. Genotyping PCR was conducted on DNA extracted from single gametophytes exhibiting developmental arrest at 10 DPS in three *ZmUbi_pro_::CrLFY1/2-i1* lines. DNA from all arrested individuals amplified positive bands for the two hairpin arms (pVec8F-PW64R and PW64F-pVec8R) and for the hygromycin resistance marker (HPTF-HPTR).

## Discussion

The results reported here reveal a role for LFY in the maintenance of apical cell activity throughout gametophyte and sporophyte shoot development in Ceratopteris. During sporophyte development, qRT-PCR and transgenic reporter lines demonstrated that *CrLFY1* is preferentially expressed in the shoot apex (whether formed during embryogenesis or de novo on fronds, and both before and after the reproductive transition); in emerging lateral organ (frond) primordia; and in pinnae and pinnules as they form on dissected fronds (Figures 3–5). Notably, active cell division is the main feature in all of these contexts. *CrLFY2* transcript levels were more uniform throughout sporophyte shoot development, in both dividing tissues and expanded fronds (Figure 3), and expression has previously been reported in roots (Himi et al., 2001). Simultaneous suppression of *CrLFY1* and *CrLFY2* activity by RNAi resulted in developmental arrest of both gametophyte and sporophyte shoot apices, with any fronds produced before termination of the sporophyte apex exhibiting abnormal morphologies (Figures 6 and 7). The severity of phenotypic perturbations in sporophytes of transgenic lines correlated with combined *CrLFY1* and *CrLFY2* transcript levels, with wild-type levels of *CrLFY2* able to fully compensate for up to a 70% reduction in *CrLFY1* levels (Figure 6). The duplicate *CrLFY* genes therefore act at least partially redundantly during shoot development in Ceratopteris.

A function for LFY in gametophyte development has not previously been reported in any land plant species. In the moss *P. patens, PpLFY1* and *PpLFY2* are expressed in both the main and lateral apices of gametophytic leafy shoots but double loss-offunction mutants develop normally, indicating that LFY is not necessary for maintenance of apical cell activity in the gametophyte (Tanahashi et al., 2005). By contrast, loss of *CrLFY* expression from the gametophyte shoot apex results in loss of apical cell activity during thallus formation in Ceratopteris (Figure 7). The different DNA binding site preferences (and hence downstream target sequences) of PpLFY and CrLFY (Sayou et al., 2014) may be sufficient to explain the functional distinction in moss and fern gametophytes, but the conserved expression pattern is intriguing given that there should be no pressure to retain that pattern in *P. patens* in the absence of functional necessity. The thalloid gametophytes of the two other extant bryophyte lineages (liverworts and hornworts) resemble the fern gametophyte more closely than mosses (Ligrone et al., 2012), but LFY function in these contexts is not yet known. Overall the data are consistent with the hypothesis that in the last common ancestor of ferns and angiosperms, LFY functioned to promote cell proliferation in the thalloid gametophyte, a role that has been lost in angiosperms where gametophytes have no apical cell and are instead just few-celled determinate structures.

**Figure 8.**
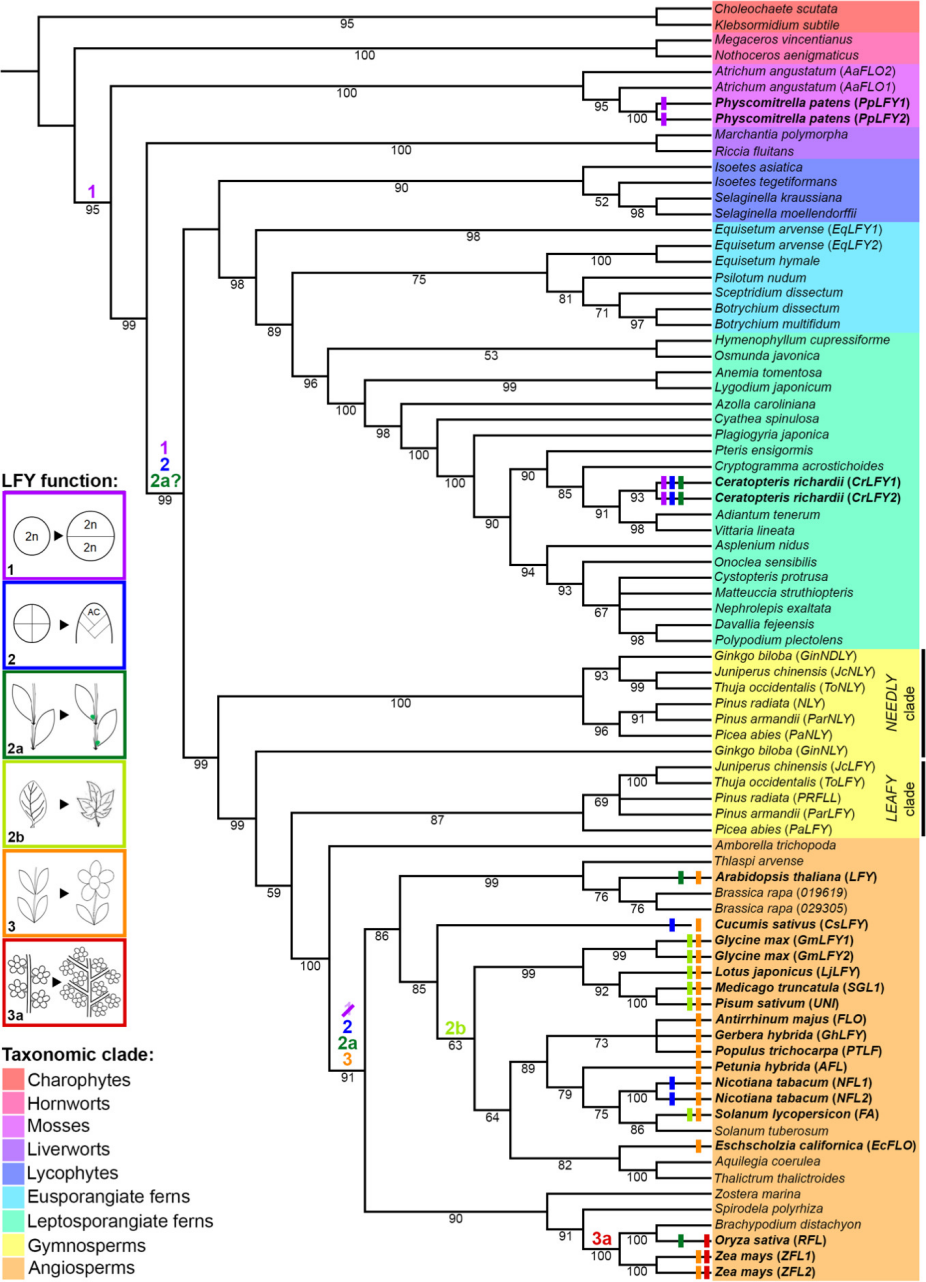
Evolutionary trajectory of LFY function. The phylogeny was reconstructed from selected LFY protein sequences representing all extant embryophyte lineages (as highlighted) and the algal sister-group. Coloured bars at the terminal branches represent different developmental functions of LFY determined from functional analysis in those species (see Supplementary file 8 for references). Coloured numbers indicate the putative points of origin of different functions inferred from available data points across the tree. 1, cell division within the sporophyte zygote; 2, maintenance of indeterminate cell fate in vegetative shoots through proliferation of one or more apical cells (AC); 2a, maintenance of indeterminate cell fate in vegetative lateral/axillary apices; 2b, maintenance of indeterminate cell fate in the margins of developing lateral organs (compound leaves); 3, specification of floral meristem identity (determinate shoot development producing modified lateral organs) and shoot transition to the reproductive phase; 3a, maintenance of indeterminate cell fate in inflorescence lateral/branch meristems (in place of floral meristem fate).

The range of reported roles for LFY in sporophyte development can be rationalized by hypothesizing three sequential changes in gene function during land plant evolution (Figure 8). First, the ancestral LFY function to promote early cell divisions in the embryo was retained in vascular plants after they diverged from the bryophytes, leading to conserved roles in *P. patens* (Tanahashi et al., 2005) and Ceratopteris (Figure 4). Second, within the vascular plants (preceding divergence of the ferns) this proliferative role expanded to maintain post-embryonic apical cell activity, and hence to enable indeterminate shoot growth. This is evidenced by *CrLFY* activity at the tips of shoots, fronds and pinnae (Figures 4–6), all of which develop from one or more apical cells (Hill, 2001; Hou and Hill, 2004). Whether fern fronds are homologous to shoots or to leaves in angiosperms is an area of debate (Tomescu, 2009; Vasco et al., 2013; Harrison and Morris, 2018), but there are angiosperm examples of LFY function in the vegetative SAM (Ahearn et al., 2001; Zhao et al., 2018), axillary meristems (Kanrar et al., 2008; Rao et al., 2008; Chahtane et al., 2013) and in actively dividing regions of compound leaves (Hofer et al., 1997; Molinero-Rosales et al., 1999; Champagne et al., 2007; Wang et al., 2008; Monniaux et al., 2017) indicating that a proliferative role in vegetative tissues has been retained in at least some angiosperm species. Consistent with the suggestion that the angiosperm floral meristem represents a modified vegetative meristem (Theiben et al., 2016), the third stage of LFY evolution could have been co-option and adaptation of this proliferation-promoting network into floral meristems, with subsequent restriction to just the flowering role in many species. This is consistent with multiple observations of *LFY* expression in both vegetative and reproductive shoots (developing cones) in gymnosperms (Mellerowicz et al., 1998; Mouradov et al., 1998; Shindo et al., 2001; Carlsbecker et al., 2004; Vázquez-Lobo et al., 2007; Carlsbecker et al., 2013; Moyroud et al., 2017) and suggests that pre-existing *LFY*-dependent vegetative gene networks might have been co-opted during the origin of specialized sporophyte reproductive axes in ancestral seed plants, prior to the divergence of angiosperms.

The proposed evolutionary trajectory for LFY function bears some resemblance to that seen for KNOX protein function. Class I *KNOX* genes are key regulators of indeterminacy in the vegetative shoot apical meristem of angiosperms (Gaillochet et al., 2015), and are required for compound leaf formation in both tomato and *Cardamine hirsuta* (Bar and Ori, 2015). In ferns, *KNOX* gene expression is observed both in the shoot apex and developing fronds (Sano et al., 2005; Ambrose and Vasco, 2016), and in *P. patens* the genes regulate cell division patterns in the determinate sporophyte (Sakakibara et al., 2008). It can thus be speculated that *LFY* and *KNOX* had overlapping functions in the sporophyte of the last common ancestor of land plants, but by the divergence of ancestral angiosperms from gymnosperms, *KNOX* genes had come to dominate in vegetative meristems whereas *LFY* became increasingly specialized for floral meristem function. Unlike *LFY*, however, there is not yet any evidence for *KNOX* function in the gametophyte of any land plant lineage, and thus if a pathway for regulating stem cell activity was co-opted from the gametophyte into the sporophyte, the LFY pathway is the more likely one.

## Materials and methods

**Key resources table:** 

Reagent type (species) or resource	Designation	Source or reference	Identifiers	Additional information
Gene (*Ceratopteris richardii*)	*CrLEAFY1* (*CrLFY1*)	Himi et al. (2001), PMID:11675598; This paper	NCBI:AB049974.2; NCBI:MH841970	cDNA only; ORF plus contiguous promoter
Gene (*C. richardii*)	*CrLEAFY2* (*CrLFY2*)	Himi et al. (2001), PMID:11675598; This paper	NCBI:AB049975.2; NCBI:MH841971	cDNA only; ORF plus contiguous promoter
Strain, strain background (*C. richardii*)	Wild type (Hn-n)	Warne and Hickok, 1987, PMID:16665325		
Genetic reagent (*C. richardii*)	*CrLFY1/2-i1*	This paper		*C. richardii* transgenic line; RNAi knockdown of *CrLFY1* and *CrLFY2* expression.
Genetic reagent (*C. richardii*)	*CrLFY1/2-i2*	This paper		*C. richardii* transgenic line; RNAi knockdown of *CrLFY1* and *CrLFY2* expression.
Genetic reagent (*C. richardii*)	*CrLFY1-i3*	This paper		*C. richardii* transgenic line; RNAi knockdown of *CrLFY1* expression.
Genetic reagent (*C. richardii*)	*CrLFY2-i4*	This paper		*C. richardii* transgenic line; RNAi knockdown of *CrLFY2* expression.
Genetic reagent (*C. richardii*)	*CrLFY1_pro_::GUS*	This paper		C. richardii transgenic line; *CrLFY1_pro_::GUS* reporter.
Genetic reagent (*C. richardii*)	*35S_pro_::GUS*	This paper		C. richardii transgenic line; *35S_pro_::GUS* reporter.
Recombinant DNA reagent	*CrLFY1_pro_*	This paper	NCBI:MH841970	CrLFY1 5' genomic fragment; Figure 1—figure supplement 2
Recombinant DNA reagent	*CrLFY2_pro_* fragment 1	This paper	NCBI:MH841971	CrLFY2 5' genomic fragment; Figure 1—figure supplement 2
Recombinant DNA reagent	*CrLFY2_pro_* fragment 2	This paper		CrLFY2 5' genomic fragment; Figure 1—figure supplement 2; Supplementary file 7
Recombinant DNA reagent	GUS	Ulmasov, 1997, PMID:9401121		Β-Glucuronidase (GUS) coding sequence
Recombinant DNA reagent	pANDA (RNAi vector)	Miki and Shimamoto (2004), PMID:15111724		
Recombinant DNA reagent	pCR4-TOPO (Cloning vector)	Invitrogen	Thermo Scientific: K457502	
Recombinant DNA reagent	pDONR207 (Gateway vector)	Invitrogen		
Recombinant DNA reagent	pBOMBER (Binary vector)	Plackett et al. (2015), PMID:26146510	NCBI:MH841969	Modified pART27 (PMID:1463857); Hygromycin resistance antibiotic selection marker
Recombinant DNA reagent	pART7 (Cloning vector)	Gleave 1992, PMID:1463857		
Recombinant DNA reagent	*ZmUbi_pro_::CrLFY1/2-i1-pANDA*	This paper		RNAi construct targeting *CrLFY1* and *CrLFY2*; Figure 6—figure supplement 1; Figure 6—figure supplement 2
Recombinant DNA reagent	*ZmUbi_pro_::CrLFY1/2-i2-pANDA*	This paper		RNAi construct targeting *CrLFY1* and *CrLFY2*; Figure 6—figure supplement 1; Figure 6—figure supplement 2
Recombinant DNA reagent	*ZmUbi_pro_::CrLFY1-i3-pANDA*	This paper		RNAi construct targeting *CrLFY1*; Figure 6—figure supplement 1; Figure 6—figure supplement 2
Recombinant DNA reagent	*ZmUbi_pro_::CrLFY2-i4-pANDA*	This paper		RNAi construct targeting CrLFY2; Figure 6—figure supplement 1; Figure 6—figure supplement 2
Recombinant DNA reagent	*CrLFY1_pro_::GUS-pBOMBER*	This paper		GUS reporter construct, *CrLFY1*; Figure 4—figure supplement 1
Recombinant DNA reagent	*35Spro::GUS-pBOMBER*	This paper		GUS reporter construct, *35S* control; Figure 4—figure supplement 1
Recombinant DNA reagent	*CrLFY1 in situ* probe (antisense)	This paper		In situ hybridisation probe; Supplementary file 6
Recombinant DNA reagent	*CrLFY1 in situ* probe (sense)	This paper		In situ hybridisation probe; Supplementary file 6
Recombinant DNA reagent	*CrLFY2 in situ* probe (antisense)	This paper		In situ hybridisation probe; Supplementary file 6
Recombinant DNA reagent	*CrLFY2 in situ* probe (sense)	This paper		In situ hybridisation probe; Supplementary file 6
Recombinant DNA reagent	^32^P-CrLFY1 probe 1	This paper		DNA gel blot probe for CrLFY1; Figure 1—figure supplement 2
Recombinant DNA reagent	^32^P-CrLFY1 probe 2	This paper		DNA gel blot probe for CrLFY1; Figure 1—figure supplement 2
Recombinant DNA reagent	^32^P-CrLFY2 probe 1	This paper		DNA gel blot probe for CrLFY2; Figure 1—figure supplement 2
Recombinant DNA reagent	^32^P-CrLFY2 probe 1	This paper		DNA gel blot probe for CrLFY2; Figure 1—figure supplement 2
Recombinant DNA reagent	^32^P-HygR probe	Plackett et al. (2014), PMID:24623851		DNA gel blot probe, T-DNA specific; Figure 4—figure supplement 1
Recombinant DNA reagent	^32^P-GUS probe	Plackett et al. (2014), PMID:24623851		DNA gel blot probe, T-DNA specific; Figure 4—figure supplement 1
Recombinant DNA reagent	^32^P-GUSlinker probe	This paper		DNA gel blot probe, T-DNA specific; Figure 6—figure supplement 2
Sequence-based reagent	CrLFY1ampF	This paper		ORF amplification, CrLFY1: 5'-ATGGATGTCTCT TTATTGCCAC-3'
Sequence-based reagent	CrLFY1ampR	This paper		ORF amplification, CrLFY1: 5'-TCAATCATAGATGC AGCTATCACTG-3'
Sequence-based reagent	CrLFY1ampF	This paper		ORF amplification, CrLFY2: 5'-ATGTTCCGATGG GAACAAAG-3'
Sequence-based reagent	CrLFY1ampR	This paper		ORF amplification, CrLFY2: 5'-TTATTCATAGCT GCAGCTGTC-3'
Sequence-based reagent	CrLFY1invF	This paper		Inverse PCR, CrLFY1: 5'-CTATGGAGTAC GAAGCACCAC-3'
Sequence-based reagent	CrLFY1invF2	This paper		Inverse PCR, CrLFY1: 5'-CGATCATTTCTT GTACTGCTCTC-3'
Sequence-based reagent	CrLFY1invF3	This paper		Inverse PCR, CrLFY1 : 5'-CAGTGCATGACCTTCGATATTG-3'
Sequence-based reagent	CrLFY1invR	This paper		Inverse PCR, CrLFY1: 5'-CAGTTGTTTCGGATCTGCAG-3'
Sequence-based reagent	CrLFY1invR2	This paper		Inverse PCR, CrLFY1: 5'-CTCCGCTTTTCATTTGAGAACG-3'
Sequence-based reagent	CrLFY1invR3	This paper		Inverse PCR, CrLFY1: 5'-CAAGAACCGCTGGAGTAAAC-3'
Sequence-based reagent	CrLFY2invF	This paper		Inverse PCR, CrLFY2: 5'-CTATGGTGTACGGAGCACTAC-3'
Sequence-based reagent	CrLFY2invF2	This paper		Inverse PCR, CrLFY2: 5'-CGTATCCAAAACAGC TTAAACTCC-3'
Sequence-based reagent	CrLFY2invF3	This paper		Inverse PCR, CrLFY2: 5'-CACTAAAGGTGCTGCTATCAAC-3'
Sequence-based reagent	CrLFY2invF4	This paper		Inverse PCR, CrLFY2: 5'-CATTGTGCTGACCTTGTGAAG-3'
Sequence-based reagent	CrLFY2invF5	This paper		Inverse PCR, CrLFY2: 5'-CGCAAAGGTTGGAA AAGAGAAC-3'
Sequence-based reagent	CrLFY2invF6	This paper		Inverse PCR, CrLFY2: 5'-CGACAACGGATCATAACCATC-3'
Sequence-based reagent	CrLFY2 invF7	This paper		Inverse PCR, CrLFY2: 5'-CAATAGTAGATT CTCCCTCCTTTAC-3'
Sequence-based reagent	CrLFY2invF8	This paper		Inverse PCR, CrLFY2: 5'-GCTCTTTAATTT GAATCACGTGTG-3'
Sequence-based reagent	CrLFY2invF9	This paper		Inverse PCR, CrLFY2: 5'-GAACAATGTGCA TGCGACTC-3'
Sequence-based reagent	CrLFY2invF10	This paper		Inverse PCR, CrLFY2: 5'-CATGTTCCGAT GGGAACAAAG-3'
Sequence-based reagent	CrLFY2invF11	This paper		Inverse PCR, CrLFY2: 5'-CATAGGGAACT CTGTAATGATGC-3'
Sequence-based reagent	CrLFY2invF12	This paper		Inverse PCR, CrLFY2: 5'-GTTTCCAG ATACTGCTGCTC-3'
Sequence-based reagent	CrLFY2invF13	This paper		Inverse PCR, CrLFY2: 5'-CATAGATGA TGCCAGTATACTCC-3'
Sequence-based reagent	CrLFY2invF14	This paper		Inverse PCR, CrLFY2: 5'-GCTCACTAT CCACAATTCATACAC-3'
Sequence-based reagent	CrLFY2invF15	This paper		Inverse PCR, CrLFY2: 5'-GTTCGTATCT GATACTTGTTTCGTG-3'
Sequence-based reagent	CrLFY2invF16	This paper		Inverse PCR, CrLFY2: 5'-CTTACTCCA CGAATGCATGC-3'
Sequence-based reagent	CrLFY2invR	This paper		Inverse PCR, CrLFY2: 5'-CAGTTGTCAC AGAGGTAGCAG-3'
Sequence-based reagent	CrLFY2invR2	This paper		Inverse PCR, CrLFY2: 5'-CCTTACGATG TATTACCCTTTGTTC-3'
Sequence-based reagent	CrLFY2invR3	This paper		Inverse PCR, CrLFY2: 5'-CAGTGACTA GGATGTCTGATACAG-3'
Sequence-based reagent	CrLFY2invR4	This paper		Inverse PCR, CrLFY2: 5'-GAAGGAGCT GAAAATGCAACTC-3'
Sequence-based reagent	CrLFY2invR5	This paper		Inverse PCR, CrLFY2: 5'-CCTGCCTCC TATGAAAACAC-3'
Sequence-based reagent	CrLFY2invR6	This paper		Inverse PCR, CrLFY2: 5'-CCTGTAAAGG AGGGAGAATCTAC-3'
Sequence-based reagent	CrLFY2invR7	This paper		Inverse PCR, CrLFY2: 5'-GCACTCCAAC GATGATGATAC-3'
Sequence-based reagent	CrLFY2invR8	This paper		Inverse PCR, CrLFY2: 5'-GCTGTACTA AGGCATCAATTCAG-3'
Sequence-based reagent	CrLFY2invR9	This paper		Inverse PCR, CrLFY2: 5'-CATCTATGATA GCACAACATCACTC-3'
Sequence-based reagent	CrLFY2invR10	This paper		Inverse PCR, CrLFY2: 5'-CACAACATC ACTCAGGACTC-3'
Sequence-based reagent	CrLFY2invR11	This paper		Inverse PCR, CrLFY2: 5'-CTGCCTCCTA TGAAAACACAAG-3'
Sequence-based reagent	CrLFY2invR12	This paper		Inverse PCR, CrLFY2: 5'-CTAGTCTTTG ATGAGGTTTCATGTC-3'
Sequence-based reagent	CrLFY2invR13	This paper		Inverse PCR, CrLFY2: 5'-CATGCAAGA AGCATGCAATTC-3'
Sequence-based reagent	CrLFY2invR14	This paper		Inverse PCR, CrLFY2: 5'-GTGTCTCCA GTAAGTATGAAACAAG-3'
Sequence-based reagent	CrLFY2invR15	This paper		Inverse PCR, CrLFY2: 5'-CATGAGGCC GTCAGACTTAC-3'
Sequence-based reagent	CrLFY2invR16	This paper		Inverse PCR, CrLFY2: 5'-CGTAACAGA CGAGCTCGATATAATAG-3'
Sequence-based reagent	CrLFY2invR17	This paper		Inverse PCR, CrLFY2: 5'-CTCTTTGCTCA TATAGCTTCAAGC-3'
Sequence-based reagent	CrLFY1 + 2 (1)-RNAi-F	This paper		T-DNA cloning, CrLFY1/2-i1: 5'-ATGGGT TTCACTGTGAATAC-3'
Sequence-based reagent	CrLFY1 + 2 (1)-RNAi-R	This paper		T-DNA cloning, CrLFY1/2-i1: 5'-TCTCCTC TTTGTTCCCTTGTG-3'
Sequence-based reagent	CrLFY1 + 2 (2)-RNAi-F	This paper		T-DNA cloning, CrLFY1/2-i2: 5'-ATGGG TTTCACTGTTAGTAC-3'
Sequence-based reagent	CrLFY1 + 2 (2)-RNAi-R	This paper		T-DNA cloning, CrLFY1/2-i2: 5'-TCTCCT CTTTGTTCCCTGGTG-3'
Sequence-based reagent	CrLFY1-RNAi-F	This paper		T-DNA cloning, CrLFY1-i3: 5'-CCTTTTCT TGCTAATGATGGC-3'
Sequence-based reagent	CrLFY1-RNAi-R	This paper		T-DNA cloning, CrLFY1-i3: 5'-CAAACAAA CTTGAAAATGATAC-3'
Sequence-based reagent	CrLFY2-RNAi-F	This paper		T-DNA cloning, CrLFY2-i4: 5'-GCCATTG CTAGCAAGGTTAT-3'
Sequence-based reagent	CrLFY2-RNAi-R	This paper		T-DNA cloning, CrLFY2-i4: 5'-CACTGCT TTGAAACTAAAAC-3'
Sequence-based reagent	pCrLFY1amp-NotF	This paper		T-DNA cloning, CrLFY1_pro_: 5'-CAGCGGCCGCTTAGATGG CTTGAGATGCTAC-3'
Sequence-based reagent	pCrLFY1amp-XbaR	This paper		T-DNA cloning, CrLFY1_pro_: 5'-CATCTAGAG GAGGCACTTCTTTACGTG-3'
Sequence-based reagent	GUSamp-XbaF	This paper		T-DNA cloning, GUS CDS: 5'-CATCTAGAC AATGGTAAGCTTAGCGGG-3'
Sequence-based reagent	GUSamp-XbaR	This paper		T-DNA cloning, GUS CDS: 5'-CCATCTAGA TTCATTGTTTGCCTCCCTG-3'
Sequence-based reagent	qCrLFY1_F2	This paper		qRT-PCR, CrLFY1: 5'-GTCCGCT ATTCGTGCAGAGA-3'
Sequence-based reagent	qCrLFY1_R2	This paper		qRT-PCR, CrLFY1 : 5'-AATTCAAGGGGG CATTGGGT-3'
Sequence-based reagent	qCrLFY2_F3	This paper		qRT-PCR, CrLFY2: 5'-GCAGTGACAATGAAGGACGC-3'
Sequence-based reagent	qCrLFY2_R3	This paper		qRT-PCR, CrLFY2: 5'-AGAATCGTGCACACTGCTCA-3'
Sequence-based reagent	qCrTBPb_F	Ganger et al. (2015), DOI:10.1139/cjb-2014–0202		qRT-PCR, CrTBP: 5'-ATGAGCCAGAGCTTTTCCCC-3'
Sequence-based reagent	qCrTBPb_R	Ganger et al. (2015), DOI:10.1139/cjb-2014–0202		qRT-PCR, CrTBP: 5'-TTCGTCTCTGACCTTTGCCC-3'
Sequence-based reagent	qCrACT1_F	Ganger et al. (2015), DOI:10.1139/cjb-2014–0202		qRT-PCR, CrActin1: 5'-GAGAGAGGCTA CTCTTTCACAACC-3'
Sequence-based reagent	qCrACT1_R	Ganger et al. (2015), DOI:10.1139/cjb-2014–0202		qRT-PCR, CrActin1: 5'-AGGAAGTTCGTA ACTCTTCTCCAA-3'
Sequence-based reagent	CrLFY1_ISH_F	This paper		In situ probes, CrLFY1: 5'-GAGGCATACA CACACGCAGT-3'
Sequence-based reagent	CrLFY1_ISH_R	This paper		In situ probes, CrLFY1: 5'-TCAATCATAGAT GCAGCTATCACTG-3
Sequence-based reagent	CrLFY2_ISH_F	This paper		In situ probes, CrLFY2: 5'-GGCTGGTTGTTA CGGATAGC-3'
Sequence-based reagent	CrLFY2_ISH_R	This paper		In situ probes, CrLFY2: 5'-TTATTCATAG CTGCAGCTGTCACTG-3'
Sequence-based reagent	CrLFY1_Probe1F	This paper		Copy number analysis, CrLFY1 probe 1: 5'-CAGG CACAAGGGAACAAAG-3'
Sequence-based reagent	CrLFY1_Probe1R	This paper		Copy number analysis, CrLFY1 probe 1: 5'-CA TAGATGCAGCTATCACTGTC-3'
Sequence-based reagent	CrLFY1_Probe2F	This paper		Copy number analysis, CrLFY1 probe 2: 5'-CACTTGAAGGTAAGCT TTATTGTAAGG-3'
Sequence-based reagent	CrLFY1_Probe2R	This paper		Copy number analysis, CrLFY1 probe 2: 5'-CAATA TTTCCGACTATACATTGAGGC-3'
Sequence-based reagent	CrLFY2_Probe1F	This paper		Copy number analysis, CrLFY2 probe 1: 5'-CAGGCA CCAGGGAACAAAG-3'
Sequence-based reagent	CrLFY2_Probe1R	This paper		Copy number analysis, CrLFY2 probe 1: 5'-CATAGC TGCAGCTGGTCACTGTC-3'
Sequence-based reagent	CrLFY2_Probe2F	This paper		Copy number analysis, CrLFY2 probe 2: 5'-CTGTAG AAGGTAAGATTCTGCTC-3'
Sequence-based reagent	CrLFY2_Probe2R	This paper		Copy number analysis, CrLFY2 probe 2: 5'-GCTT ATGGTACAGAATAAGTAGAGG-3'
Sequence-based reagent	HygF2	Plackett et al. (2014), PMID:24623851		T-DNA gel blot probe, Hyg^R^: 5'-CTTCTACA CAGCCATCGGTC-3'
Sequence-based reagent	HygR	Plackett et al. (2014), PMID:24623851		T-DNA gel blot probe, Hyg^R^: 5'-CCGATGGT TTCTACAAAGATCG-3'
Sequence-based reagent	GH3seqF3	Plackett et al. (2014), PMID:24623851		T-DNA gel blot probe, GUS: 5'-CTTCGCT GTACAGTTCTTTCG-3'
Sequence-based reagent	GH3seqR4	Plackett et al. (2014), PMID:24623851		T-DNA gel blot probe, GUS: 5'-CACTCATT ACGGCAAAGTGTG-3'
Sequence-based reagent	GUSlinkerseqF	This paper		T-DNA gel blot probe, RNAi: 5'-CTGATT AACCACAAACCGTTCTAC-3'
Sequence-based reagent	GUSlinkerseqR	This paper		T-DNA gel blot probe, RNAi: 5'-CTGATA CTCTTCACTCCACATG-3'
Sequence-based reagent	HPT-F	Miki and Shimamoto (2004), PMID:15111724		RNAi genotyping, Hyg^R^: 5'-GAGCCTGACCTA TTGCATCTCC-3'
Sequence-based reagent	HPT-R	Miki and Shimamoto (2004), PMID:15111724		RNAi genotyping, Hyg^R^: 5'-GGCCTCCAG AAGAAGATGTTGG-3'
Sequence-based reagent	pVec8F	Miki and Shimamoto (2004), PMID:15111724		RNAi genotyping, RNAi hairpin: 5'-TTTAGC CCTGCCTTCATACG-3'
Sequence-based reagent	pVec8R	Miki and Shimamoto (2004), PMID:15111724		RNAi genotyping, RNAi hairpin: 5'-ATTGC CAAATGTTTGAACGA-3'
Sequence-based reagent	PW64F	This paper		RNAi genotyping, RNAi hairpin: 5'-CATGAA GATGCGGACTTACG-3'
Sequence-based reagent	PW64R	This paper		RNAi genotyping, RNAi hairpin: 5'-ATCCAC GCCGTATTCGG-3'
Sequence-based reagent	pCrLFY1genoF1	This paper		*CrLFY1_pro_::GUS* genotyping: 5'-CTTAGA TGGCTTGAGATGCTAC-3'
Sequence-based reagent	pCrLFY1genoF2	This paper		*CrLFY1_pro_::GUS* genotyping: 5'-CTCTCT TCTTGCTTGTGTTGTG-3'
Sequence-based reagent	pCrLFY1genoF3	This paper		*CrLFY1_pro_::GUS* genotyping: 5'-CAACTGGCAACAGGTGATG-3'
Sequence-based reagent	pCrLFY1genoF4	This paper		*CrLFY1_pro_::GUS* genotyping: 5'-CAGTCTTAGTTCAACTGCATTCG-3'
Sequence-based reagent	pCrLFY1genoR	This paper		*CrLFY1_pro_::GUS* genotyping: 5'-AGGAGGCACTTCTTTACGTG-3'
Sequence-based reagent	GUSgenoR	This paper		*CrLFY1_pro_::GUS + 35S_pro_::GUS* genotyping: 5'-CATTGTTTG CCTCCCTGC-3'
Sequence-based reagent	35SgenoF	This paper		*35S_pro_::GUS* genotyping: 5'-CTGAGCTTAACAGCACAGTTG-3'
Sequence-based reagent	OCS3’genoR	This paper		*35S_pro_::GUS* genotyping: 5'-CATCACTAGTAAGCTAGCTTGC-3'
Commercial assay or kit	Phusion high-fidelity polymerase	Thermo Scientific	Thermo Scientific: F530S	
Commercial assay or kit	Gateway LR clonase II enzyme mix	Invitrogen	Thermo Scientific: 11791100	
Commercial assay or kit	QIAGEN Plasmid Maxi Kit	QIAGEN	QIAGEN:12163	
Commercial assay or kit	Whatman Nytran nylon blotting membrane	GE Healthcare	GE Healthcare: 10416294	
Commercial assay or kit	Random Primers DNA Labelling kit	Invitrogen	Thermo Scientific: 18187013	
Commercial assay or kit	Carestream Kodak autoradiography GBX developer and fixer	Sigma-Aldrich	Sigma-Aldrich: Z354147	
Commercial assay or kit	Carestream Kodak Biomax XAR film	Sigma-Aldrich	Sigma-Aldrich:F5763	
Commercial assay or kit	iTaq universal SYBR Green mastermix	Bio-Rad	Bio-Rad:1725120	
Commercial assay or kit	DIG-labelling mix	Roche Applied Sciences	Roche:11277073910	
Commercial assay or kit	T3 RNA polymerase	Roche Applied Sciences	Roche:11031163001	
Commercial assay or kit	T7 RNA polymerase	Roche Applied Sciences	Roche:10881767001	
Commercial assay or kit	Anti-DIG antibody	Roche Applied Sciences	Roche:11093274910; RRID:AB_2313639	
Commercial assay or kit	NBT/BCIP stock solution	Roche Applied Sciences	Roche:11681451001	
Chemical compound, drug	Potassium ferricyanide (K₃Fe(CN)₆)	Sigma-Aldrich	Sigma:P8131	
Chemical compound, drug	X-GlcA (CHA salt)	Melford Scientific	Melford:MB1021	
Chemical compound, drug	CTP, [ɑ-^32^P]	Perkin Elmer	Perkin Elmer: BLU008H250UC	
Software, algorithm	IQ-TREE	Nguyen et al. (2015), PMID:25371430		http://www.iqtree.org/
Software, algorithm	iTOL	Letunic and Bork (2016), PMID:27095192		https://itol.embl.de/
Software, algorithm	ClustalW	Li et al. (2015), PMID:25845596	RRID:SCR_002909	https://www.ebi.ac.uk/Tools/msa/clustalw2/
Software, algorithm	TBLASTX	Altschul et al. (1990), PMID:2231712	RRID:SCR_011823	https://blast.ncbi.nlm.nih.gov/Blast.cgi
Software, algorithm	GeneWise	Birney et al. (2004), PMID:15123596	RRID:SCR_015054	https://www.ebi.ac.uk/Tools/psa/genewise
Software, algorithm	GraphPad Prism	GraphPad Software Inc.	RRID:SCR_002798	https://www.graphpad.com/scientific-software/prism/
Software, algorithm	Adobe Photoshop CS4	Adobe	RRID:SCR_014199	
Other	Biolistic PDS-1000/He Particle Delivery System	Bio-Rad	Bio-Rad:1652257	
Other	CFX Connect Real-Time PCR Detection System	Bio-Rad	Bio-Rad:1855201	
Other	Zeiss Axioplan microscope	Zeiss		
Other	Nikon Microphot-FX microscope	Nikon		
Other	MicroPublisher 3.3 RTV camera	Qimaging		

### Plant materials and growth conditions

All experimental work was conducted using *Ceratopteris richardii* strain Hn-n (Warne and Hickok, 1987). Plant growth conditions for Ceratopteris transformation and DNA gel blot analysis of transgenic lines were as previously described (Plackett et al., 2015).

### Phylogenetic analysis

A dataset of 99 aligned LFY protein sequences from a broad range of streptophytes was first retrieved from Sayou et al. (2014). The dataset was pruned and then supplemented with further sequences (Supplementary file 1) to enable trees to be inferred that would (i) provide a more balanced distribution across the major plant groups and (ii) infer fern relationships. Only a subset of available angiosperm sequences was retained (keeping both monocot and dicot representatives) but protein sequences from other angiosperm species where function has been defined through loss-of-function analyses were added from NCBI – *Antirrhinum majus* FLO AAA62574.1 (Coen et al., 1990), *Pisum sativum* UNI AAC49782.1 (Hofer et al., 1997), *Cucumis sativus* CsLFY XP_004138016.1 (Zhao et al., 2018), *Medicago truncatula* SGL1 AY928184 (Wang et al., 2008), *Petunia hybrida* ALF AAC49912.1 (Souer et al., 1998), *Nicotiana tabacum* NFL1 AAC48985.1 and NFL2 AAC48986.1 (Kelly, 1995), *Eschscholzia californica* EcFLO AAO49794.1 (Busch and Gleissberg, 2003), *Gerbera hybrida* cv. ‘Terraregina’ GhLFY ANS10152.1 (Zhao et al., 2016), *Lotus japonicus* LjLFY AAX13294.1 (Dong et al., 2005) and *Populus trichocarpa* PTLF AAB51533.1 (Rottmann et al., 2000). To provide better resolution within and between angiosperm clades, sequences from *Spirodela polyrhiza* (32G0007500), *Zostera marina* (27g00160.1), *Aquilegia coerulea* (5G327800.1) and *Solanum tuberosum* (PGSC0003DMT400036749) were added from Phytozome v12.1 (https://phytozome.jgi.doe.gov/pz/portal.html). Genome sequence from the early-diverging Eudicot *Thalictrum thalictroides* was searched by TBLASTX (Altschul et al., 1990) (https://blast.ncbi.nlm.nih.gov/Blast.cgi?PROGRAM=tblastx&PAGE_TYPE=BlastSearch&BLAST_SPEC=&LINK_LOC=blasttab) with nucleotide sequence from the Arabidopsis *LFY* gene. A gene model was derived from sequence in two contigs (108877 and 116935) using Genewise (Birney et al., 2004) (https://www.ebi.ac.uk/Tools/psa/genewise/). Gymnosperm sequences were retained from *Ginkgo biloba* and from a subset of conifers included in Sayou et al. (2014), whilst sequences from conifers where *in situ* hybridization patterns have been reported were added from NCBI – *Pinus radiata* PRFLL AAB51587.1 and NLY AAB68601.1 (Mellerowicz et al., 1998; Mouradov et al., 1998) and *Picea abies* PaLFY AAV49504.1 and PaNLY AAV49503.1 (Carlsbecker et al., 2004). Fern sequences were retained except *Angiopteris spp* sequences which consistently disrupted the topology of the tree by grouping with gymnosperms. To better resolve relationships within the ferns, additional sequences were identified in both NCBI and 1KP (Matasci et al., 2014) databases. The protein sequence from *Matteuccia struthiopteris* AAF77608.1 MatstFLO (Himi et al., 2001) was retrieved from NCBI. Further sequences from horsetails (2), plus eusporangiate (1) and leptosporangiate (53) ferns were retrieved from the 1KP database (https://db.cngb.org/blast/) using BLASTP and the MatstFLO sequence as a query. Lycophyte and bryophyte sequences were all retained, but the liverwort *Marchantia polymorpha* predicted ORF sequence was updated from Phytozome v12.1 (Mpo0113s0034.1.p), the hornwort *Nothoceros* genome scaffold was replaced with a translated full length cDNA sequence (AHJ90704.1) from NCBI and two additional lycophyte sequences were added from the 1KP dataset (*Isoetes tegetiformans* scaffold 2013584 and *Selaginella kraussiana* scaffold 2008343). All of the charophyte scaffold sequences were substituted with *Coleochaete scutata* (AHJ90705.1) and *Klebsormidium subtile* (AHJ90707.1) translated full-length cDNAs from NCBI.

The new/replacement sequences were trimmed and amino acids aligned to the existing alignment from Sayou et al. (2014) using CLUSTALW (Li et al., 2015) (Supplementary file 2 and 3). The best-fitting model parameters (JTT + I + G4) were estimated and consensus phylogenetic trees were run using Maximum Likelihood from 1000 bootstrap replicates, using IQTREE (Nguyen et al., 2015). Two trees were inferred. The first contained only a subset of fern and allied sequences to achieve a more balanced distribution across the major plant groups (81 sequences in total) (Figure 8), whereas the second used the entire dataset (120 sequences ~ 50% of which are fern and allied sequences – Figure 1—figure supplement 1). The data were imported into ITOL (Letunic and Bork, 2016) to generate the pictorial representations. All branches with less than 50% bootstrap support were collapsed. Relationships within the ferns (Figure 1) were represented by pruning the lycophyte and fern sequences (68 in total) from the tree containing all available fern sequences (**Figure 1—figure supplement 1).**

### *CrLFY* locus characterization and DNA gel blot analysis

Because no reference genome has yet been established for Ceratopteris (or any fern), *CrLFY* copy number was quantified by DNA gel blot analysis. Ceratopteris genomic DNA was hybridized using both the highly conserved LFY DNA-binding domain diagnostic of the *LFY* gene family (Maizel et al., 2005) and also gene copy-specific sequences (Figure 1—figure supplement 2). CrLFY1 and CrLFY2 share 85% amino acid similarity, compared to 65% and 44% similarity of each to AtLFY. DNA gel blotting and hybridization was performed as described previously (Plackett et al., 2014). The results supported the presence of only two copies of *LFY* within the Ceratopteris genome. All primers used in probe preparation are supplied in the Key Resources Table.

Genomic sequences for *CrLFY1* and *CrLFY2* open reading frames (ORFs) were amplified by PCR from wild-type genomic DNA using primers designed against published transcript sequences (Himi et al., 2001). ORFs of 1551 bp and 2108 bp were obtained, respectively (Figure 1—figure supplement 2). Exon structure was determined by comparison between genomic and transcript sequences. The native promoter region of *CrLFY1* was amplified from genomic template by sequential rounds of inverse PCR with initial primer pairs designed against published *CrLFY1* 5’UTR sequence and additional primers subsequently designed against additional contiguous sequence that was retrieved. A 3.9 kb contiguous promoter fragment was isolated for *CrLFY1* containing the entire published 5’UTR and 1.9 kb of additional upstream sequence (Figure 1—figure supplement 2). Repeated attempts were made to obtain a *CrLFY2* promoter fragment but this proved impossible in the absence of a reference genome. Some sequence contiguous with the *CrLFY2* ORF was obtained by inverse PCR using primers designed against the previously published 5’UTR sequence of the *CrLFY2* transcript (Himi et al., 2001). This sequence was extended to 1016 bp in length using additional primers against the isolated genomic sequence but this fragment did not contain the entire published 5’UTR. Numerous rounds of inverse PCR generated a second 3619 bp genomic fragment containing sequence identical to the remaining 5’UTR (see Figure 1—figure supplement 2, Supplementary file 7) but the presumed connecting sequence between these two fragments could not be amplified despite many attempts. It was eventually concluded that either the intervening promoter fragment was too long to amplify or that it was too GC rich for amplification. All primers used in ORF amplification and inverse PCR are supplied in the Key Resources table. The contiguous sequences obtained for the *CrLFY1* and *CrLFY2* genomic loci have been submitted to Genbank (accessions MH841970 and MH841971, respectively).

### qRT-PCR analysis of gene expression

RNA was extracted from Ceratopteris tissues using the Spectrum Total Plant RNA kit (Sigma-Aldrich, St. Louis, MO) and 480 ng were used as template in iScript cDNA synthesis (Bio-Rad). *CrLFY1* and *CrLFY2* locus-specific qRT-PCR primers were designed spanning intron 1. Amplification specificity of primers was validated via PCR followed by sequencing. qRT-PCR of three biological replicates and three technical replicates each was performed in a Bio-Rad CFX Connect with iTaq Universal SYBR Green Supermix (Bio-Rad, Hercules, CA). Primer amplification efficiency was checked with a cDNA serial dilution. Efficiency was determined using the slope of the linear regression line as calculated by Bio-Rad CFX Connect software. Primer specificity was tested via melting curve analysis, resulting in a single peak per primer set. *CrLFY* expression was calculated using the 2^- ΔΔCt^ method (Livak and Schmittgen, 2001) and normalized against the geometric mean of the expression of two endogenous reference genes (Hellemans et al., 2007), *CrACTIN1* and *CrTATA-BINDING PROTEIN (TBP)* (Ganger et al., 2015). The standard deviation of the Ct values of each reference gene was calculated to ensure minimal variation (<3%) in gene expression. Error bars represent ± the standard error of the mean of the 2 ^ΔΔ Ct^ values. All primers used in qRT-PCR are supplied in the Key Resources table.

Relative expression values of *CrLFY* from qRT-PCR were compared by one or two-way analysis of variance (ANOVA) for developmental stages followed by Tukey’s or Sidak’s multiple comparisons, respectively. To test whether genes were downregulated in transgenic RNAi lines, two-way ANOVA was perfomed with gene (*CrLFY1* or *CrLFY2*) and transgenic line as factors, with ‘gene’ as a repeated factor when all transgenic lines had the same number of replicates. Where appropriate, expression of each gene in each line was compared to the expression of the respective control by Dunnet comparisons. Control plants had been transformed and were hygromycin-resistant, but did not contain the RNAi hairpin that triggers gene silencing (non-hairpin controls, NHC). For all experiments, NHCs were grown alongside transgenic lines. qRT-PCR of transgenic lines was necessarily conducted across several plates, each including a representative NHC, and statistical comparisons were performed within each plate relative to its respective control. The significance threshold (p) was set at 0.05. All statistical analyses were performed in Prism v. 6.0 (GraphPad Software, Inc., La Jolla, CA).

### Generation of GUS reporter constructs

The *CrLFY1_pro_::GUS* reporter construct (Figure 4—figure supplement 1) was created by cloning the *CrLFY1* promoter into pART7 as a *Not*I-*Xba*I restriction fragment, replacing the existing *35S* promoter. A β-Glucuronidase (GUS) coding sequence (Ulmasov, 1997) was cloned downstream of *pCrLFY1* as an *Xba*I-*Xba*I fragment. The same GUS *Xba*I-*Xba*I fragment was also cloned into pART7 to create a *35S_pro_::GUS* positive control (Figure 4—figure supplement 4). The resulting *CrLFY1_pro_::GUS::ocs* and *35S_pro_::GUS::ocs* cassettes were each cloned as *Not*I-*Not*I fragments into the pART27-based binary transformation vector pBOMBER carrying a hygromycin resistance marker previously optimized for Ceratopteris transformation (Plackett et al., 2015). All primers used in GUS reporter component amplification are supplied in the Key Resources table.

### Generation of RNAi constructs

RNAi constructs were designed and constructed using the pANDA RNAi expression system (Miki and Shimamoto, 2004). Four RNAi fragments were designed, two targeting a conserved region of the *CrLFY1* and *CrLFY2* coding sequence (77% nucleotide identity) using sequences from either *CrLFY1* (*CrLFY1/2-i1*) or *CrLFY2* (*CrLFY1/2-i2*), and two targeting gene-specific sequence within the 3’UTR of *CrLFY1* (*CrLFY1-i3*) or *CrLFY2* (*CrLFY2-i4*) (Figure 6—figure supplement 1). Target fragments were amplified from cDNA and cloned into Gateway-compatible entry vector pDONR207 (Invitrogen, Carlsbad, CA). Each sequence was then recombined into the pANDA expression vector via Gateway LR cloning (Invitrogen, Carlsbad, CA). All primers used in RNAi target fragment amplification are supplied in the Key Resources table.

### Generation of transgenic lines

Transformation of all transgenes into wild-type Hn-n Ceratopteris callus was performed as previously described (Plackett et al., 2015). T_0_ sporophyte shoots were regenerated from transformed callus tissue, with each round of transformation using multiple separate pieces of callus as starting material. Transgenic T_1_ spores were harvested from these T_0_ shoots, germinated to form T_1_ gametophytes and then self-fertilized to produce T_1_ sporophytes. T_1_ sporophytes were assessed for T-DNA copy number by DNA gel blot analysis (Figure 4—figure supplement 2; Figure 6—figure supplement 3) and the presence of full-length T-DNA insertions was confirmed through genotyping PCR (Figure 4—figure supplements 3 and 4). All primers used in genotyping reactions are supplied in the Key Resources table. For characterization of RNAi lines, T_2_ spores were collected from individuals that either contained the full transgene construct or from segregants in which the RNAi hairpin was absent.

### GUS staining

GUS activity analysis in *CrLFY1_pro_::GUS* transgenic lines was conducted in the T_1_ generation. GUS staining was conducted as described previously (Plackett et al., 2014). Optimum staining conditions (1 mg/ml X-GlcA, 5 μM potassium ferricyanide) were determined empirically. Tissue was cleared with sequential incubations in 70% ethanol until no further decolorization occurred. GUS-stained gametophytes were imaged with a Zeiss Axioplan microscope and GUS-stained sporophytes imaged with a dissecting microscope, both mounted with Q-imaging Micro-published 3.3 RTV cameras. Images were minimally processed for brightness and contrast in Photoshop (CS4).

### Phenotypic characterization

Phenotypic characterization of RNAi transgenic lines was conducted in the T_2_ or T_3_ generation. Isogenic lines were obtained by isolating hermaphrodite gametophytes in individual wells at approximately 7 DPS (or when the notch became visible, whichever came first) and flooding them once they had developed mature gametangia (at approximately 9 DPS). All transgenic lines were grown alongside both wild-type and no hairpin controls, and phenotypes observed and recorded daily. Gametophytes exhibiting altered phenotypes were imaged at approximately 10 DPS with a Nikon Microphot-FX microscope. Sporophytes with abnormal phenotypes were imaged with a dissecting microscope.

### *In situ* hybridization

Antisense and sense RNA probes for *CrLFY1* and *CrLFY2* were amplified and cloned into pCR 4-TOPO (Invitrogen) and DIG-labelled according to the manufacturer’s instructions (Roche, Indianapolis, IN). Probes were designed to include the 5’UTR and ORF (*CrLFY1* 521 bp 5’UTR and 1113 bp ORF; *CrLFY2* 301 bp 5’UTR and 1185 bp ORF) (Supplementary file 6). All primers used in *in situ* probe amplification are supplied in the Key Resources table. We were unable to identify fragments that distinguished the two genes in whole mount in situ hybridizations. Tissue was fixed in FAA (3.7% formaldehyde, 5% acetic acid; 50% ethanol) for 1–4 hr and then stored in 70% ethanol. Whole mount *in situ* hybridization was carried out based on Hejátko et al. (2006), with the following modifications: hybridization and wash steps were carried out in 24-well plates with custom-made transfer baskets (0.5 mL microcentrifuge tubes and 30 µm nylon mesh, Small Parts Inc., Logansport, IN). Permeabilization and post-fixation steps were omitted depending on tissue type to avoid damaging fragile gametophytes, Acetic Anhydride (Sigma-Aldrich) and 0.5% Blocking Reagent (Roche) washing steps were added to decrease background staining, and tissue was hybridized at 45°C. Photos were taken under bright-field with a Q-imaging Micro-publisher 3.3 RTV camera mounted on a Nikon Microphot-FX microscope. Images were minimally processed for brightness and contrast in Photoshop (CS4).

## Additional files

Supplementary file 1. LFY sequences included in phylogenetic analysis (in addition to Sayou et al., 2014) dataset).

Supplementary file 2. Alignment of all 120 LFY amino acid sequences used in phylogenetic analysis.

Supplementary file 3. Alignment of LFY amino acid sequences used in phylogenetic analysis (ferns only).

Supplementary file 4. Statistical comparison of *CrLFY* transcript levels between different ontogenetic stages.

Supplementary file 5. Specificity of *CrLFY* RNAi target sequences. Alignment (prepared using Clustal Omega) of the full length transcript sequences for *CrLFY1* and *CrLFY2*, with nucleotide identity between the two gene copies denoted by a subtending asterisk. The CDS for each gene is highlighted in bold. The target sequences for each RNAi construct are highlighted in light blue (*CrLFY1/2-i1*), light green (*CrLFY1/2-i2*), dark blue (*CrLFY1-i3*) or dark green (*CrLFY2-i4*). The *CrLFY1/2-i1* and *CrLFY1/2-i2* target sequences each have 77% similarity to the opposing gene transcript (BLAST2n, discontiguous megablast for highly similar sequences). The full-length *CrLFY1-i3* and *CrLFY2-i4* target sequences do not demonstrate significant sequence similarity to the opposing gene transcript or 3’UTR alone (BLAST2n, blastn for somewhat similar sequences) but short regions of similarity within the target sequence might explain the cross-reactivity observed.

Supplementary file 6. Predicted hybridization and specificity of *CrLFY in situ* hybridization probes. Alignment (prepared using Clustal Omega) of full length *CrLFY1* and *CrLFY2* transcript sequences, with nucleotide identity between the two paralogs denoted by a subtending asterisk. The coding sequence (CDS) for each gene copy is highlighted in bold. Predicted sites of hybridization for the two probes are highlighted in blue (*CrLFY1*) and yellow (*CrLFY2*) respectively, with PCR primer sites underlined. The in situ probes span the complete CDS and 5’UTR of each gene copy. The *CrLFY1* probe sequence shows 79% nucleotide identity to the *CrLFY2* transcript (BLAST2n, discontiguous megablast for highly similar sequences). The *CrLFY2* probe shows 79% nucleotide identity to the *CrLFY1* transcript (BLAST2n, discontiguous megablast for highly similar sequences).

Supplementary file 7. Amplified *CrLFY2* genomic fragment (3619 bp), not connected directly to *CrLFY2* open reading frame. Sequence highlighted in green corresponds to published *CrLFY2* 5’UTR (Himi et al., 2001)

Supplementary file 8. Summary of published reports of LFY function in a range of angiosperm species. All citations included in reference list of main article (Bradley et al., 1996; Blázquez et al., 1997; Bradley et al., 1997; Kyozuka et al., 1998; Pnueli et al., 1998; Ratcliffe et al., 1999; Gourlay et al., 2000; Bomblies et al., 2003; Becker et al., 2005; Meng et al., 2007; Souer et al., 2008; Wreath et al., 2013).

Transparent reporting form

### Data availability

All data generated or analysed during this study are included in the manuscript and supporting files. Source data files have been provided for Figures 3 and 6. Sequences and alignments for phylogenetic analyses are included in Supplementary files 1-3.
