# Supplementary material for: LEAFY maintains apical stem cell activity during shoot development in the fern Ceratopteris richardii
Source: eLife. 2018 Oct 24;7:e39625. doi: 10.7554/eLife.39625 (PMC6200394; doi:10.7554/eLife.39625)
Supplement: Supplementary file 1. [file elife-39625-supp1.docx]

**Supplementary File 1. LFY sequences included in phylogenetic analysis (in addition to Sayou et al. 2014 dataset).**

**NCBI (https://www.ncbi.nlm.nih.gov/):**

> Klebsormidium subtile [AHJ90707.1](https://www.ncbi.nlm.nih.gov/protein/586946493)

MQGHGLAGYRDQDSLQQSLDISSQSFHRESLRDQAYFQNVLQSSLQSHALQSQSLQSLQNHGLQSHSLQNNSLQSHSLQSHGGLQSQTLQAMGLQAPEAQNLGIANQGAHNPTAYLSQENPILPHSFNHQFQVQQAGGLSSQQHLPQQTQQVASVAEQFDDSRVMNDLLKDYGVREHTISRMEEEGFNLNTLLSMNDEEVAEAIKMMISECGINFLVGERFGVKMACRSGRKWLEEKRALLENPPAPAPPKKRKRLDGKASQGVSEHVLASLVNGGLENESEEADEETVQKQSELSGRDKSKTTPKKKKGEGDEATSRGPAFQVTAFGEEGHGKKQGLDYLFELYGEAGRILEDIRAQERATGKKPSAKVNNLVYRYAKQRGMGFINKPKMRQYLHCYALHCLDVGTSNAIRMTCRDRNKTLQAWAECCYEPLLQMARVRGYNLESLFQL

SPALAIWNVPKQLEKLCEEEKDRLGELGSGQLDQQPLPPPHRQGA

> Nothoceros aenigmaticus [AHJ90704.1](https://www.ncbi.nlm.nih.gov/protein/586946487) MGQKDVAVRHRDALMVHKDNSLGPRESRVLDQGFSSMGRREAKTLDELFRDYGVRPSTLETVAQMGFTVGTMINMTDDELDSLIKTMIENYRVDLLVGEKFGIKSAVRAERRLLDEVDRVRIEMKARTESMMELKGQYKVSDCGTGLGGVAMVSGGNGAAPGGLANVCAPSPKLAATKARKKKDIKDELSHPLLSGNVLAAMSPGALPGLLSELSSDSGKVDLGKRRAKRKRKVKEPGEEGDDRPREHPFVVTEPGELARGKKNGLDYLFNLYEQAGKFLEEVQHIAREKGEKCPTKVTNQVFRHAKVQGAGYINKPKMRQYVHCYALHCLASDKSDELRRCCKERGENVGAWCQACYLPLIKMAKEKSWDIEGLFNSHDKLKIWYVPKKLIQLCHQEKSK

> Coleochaete scutata [AHJ90705.1](https://www.ncbi.nlm.nih.gov/protein/586946489)

MANSQYKNLDTLFHNYGLRPDTLAVMNQMGFTVSTLCNMTEQEIDIMVKMMVENLNMDLLQGEKFGIKAAIRSERRQLEEAMERSRVEATGFGARKFHLDEEPVLMALPESTQMHPPVENGVARVVAKGKKRMDEGEHQQGGSVDSNGWRQVNEAMVGDPLQFDAPVVDDGDGLKKKPKKQKRQKEPDGEEERPREHPFVVTEPGEAARGKKNGLDYLFQLYEESAKLLIEIQQLSLEKGEKVPTKVTNQVFRHAKQRGMGFINKPKMRQYVHCYALHCIAPAESKQAATRLQGAWRKCWPVVSGMLRATRVHCEGPRMGFGSHIQQP

>Pisum sativum AAC49782.1 UNIFOLIATA

MDPDAFTASLFKWDPRTVLSTAPSPRPQLLDYAVTPTTAPMTYHPARLPRELGGLEELFQAYGIRYYTAAKIAELGFTVSTLVDMKDDELDDMMNSLSQIFRWDLLVGERYGIKAAIRAERRRLDEEEIKRRGLLSGDTTNALDALSQEGLSEEPVVQREKEAMGSGGGSTWEVAVVEERRKRQQIRRRRMKMKGNDHGENEEGEEEEEDNISGGGVGGGERQREHPFIVTEPGEVARGKKNGLDYLFHLYEQCREFLIQVQAIAKERGEKCPTKVTNQVFRYAKKAGASYINKPKMRHYVHCYALHCLDEEVSNELRRGFKERGENVGAWRQACYKPLVAIAARQGWDIDAIFNAHPRLSIWYVPTKLRQLCHAERNGAAASSSVSVGTTHLPF

>Cucumis sativus XP_004138016.1

MDPETLSATLFKWEPREMVGGGSGAPLPAPLPPYSLRPRELGLGGLEDLFQAYGIRYYTAAKIAELGFTVSTLVDMKDEELEDMMNSLSHIFRWDLLVGERYGIKAAVRAERRRLDDEIESSRRRHLLSNDTTTNVVLDALSQEGLSEEPVQQEKEAVGSGGGGGAWEAVVAAEMRKKQRRRNGSKKKQVIAMEEDNDENEGAGDEEENMEVSERQREHPFIVTEPGEVARGKKNGLDYLFHLYEQCREFLIQVQNIAKERGEKCPTKVTNQVFRYAKKAGASYINKPKMRHYVHCYALHCLDEEASNALRSVFKERGENVGAWRQACYKPLVTIAGTQGWDIDAIFNAHPRLAIWYVPTKLRQLCHAERNGSTASTSTASAHLPF

>Medicago truncatula AAX22220.1 SGL1

MDPDAFTASLFKWDPRTVLPTAPPLRPQLLDYAVTPSTAPSPYYPARLPRELGGLEELFQAYGIRYYTAAKIAELGFTVSTLVDMKDDELDDMMNSLSQIFRWDLLVGERYGIKAAIRAERRRLDEEEIKRRGLLSGDTTNALDALSQEGLSEEPVVQREKEAVGSGGGSTWEVAVVEERRKRQQIRRRRMKMKGNGDHGENEEGDEEEEDNISGGGERQREHPFIVTEPGEVARGKKNGLDYLFHLYEQCREFLIQVQAIAKERGEKCPTKVTNQVFRYAKKAGASYINKPKMRHYVHCYALHCLDEEVSNELRRGFKERGENVGAWRQACYKPLVAIAARQGWDIDAIFNAHPRLSIWYVPTKLRQLCHAERNSAAASSSVSVGTAHLPF

>Antirrhinum majus AAA62574.1 FLO

MDPDAFLFKWDHRTALPQPNRLLDAVAPPPPPPPQAPSYSMRPRELGGLEELFQAYGIRYYTAAKIAELGFTVNTLLDMRDEELDEMMNSLCQIFRWDLLVGERYGIKAAVRAERRRIDEEEVRRRHLLLGDTTHALDALSQEGLSEEPVQQEKEAMGSGGGGVGGVWEMMGAGGRKAPQRRRKNYKGRSRMASMEEDDDDDDDETEGAEDDENIVSERQREHPFIVTEPGEVARGKKNGLDYLFHLYEQCRDFLIQVQTIAKERGEKCPTKVTNQVFRYAKKAGANYINKPKMRHYVHCYALHCLDEAASNALRRAFKERGENVGAWRQACYKPLVAIAARQGWDIDTIFNAHPRLSIWYVPTKLRQLCHAERSSAAVAATSSITGGGPADHLPF

>Petunia x hybrida AAC49912.1 ALF

MDPEAFSASLFKWDPRGAMPPPNRLLEAVAPPQPPPPPLPPPQPLPPAYSIRTRELGGLEEMFQAYGIRYYTAAKITELGFTVNTLLDMKDDELDDMMNSLSQIFRWELLVGERYGIKAAIRAERRRLEEEEGRRRHILSDGGTNVLDALSQEGLSEEPVQQQEREAAGSGGGGTAWEVVAPGGGRMRQRRRKKVVVGRERRGSSMEEDEDTEEGQEDNEDYNINNEGGGGISERQREHPFIVTEPGEVARGKKNGLDYLFHLYEQCRDFLIQVQNIAKERGEKCPTKVTNQVFRFAKKAGASYINKPKMRHYVHCYALHCLDEDASNALRRAFKERGENVGAWRQACYKPLVAIAARQGWDIDAIFNGHPRLSIWYVPTKLRQLCHSERSNAAAAASTSVSGGGVDHLPHF

>Nicotiana tabacum AAC48985.1 NFL1

MDPEAFSASLFKWDPRGAMPPPTRLLEAAVAPPPPPPVLPPPQPLSAAYSIRTRELGGLEELFQAYGIRYYTAAKIAELGFTVNTLLDMKDEELDDMMNSLSQIFRWELLVGERYGIKAAIRAERRRLEEEELRRRSHLLSDGGTNALDALSQEGLSEEPVQQQEREAVGSGGGGTTWEVVAAVGGGRMKQRRRKKVVSTGRERRGRASAEEDEETEEGQEDEWNINDAGGGISERQREHPFIVTEPGEVARGKKNGLDYLFHLYEQCRDFLIQVQNIAKERGEKCPTKVTNQVFRYAKKAGASYINKPKMRHYVHCYALHCLDEEASNALRRAFKERGENVGAWRQACYKPLVAIAARQGWDIDTIFNAHPRLAIWYVPTRLRQLCHSERSNAAAAASSSVSGGVGDHLPHF

>Nicotiana tabacum AAC48986.1 NFL2

MDPEAFSASLFKWDPRGAMPPPTRLLEAAVAPPPPPPALPPPQPLSAAYSIKTRELGGLEELFQAYGIRYYTAAKIAELGFTVNTLLDMKDEELDDMMNSLSQIFRWELLVGERYGIKAAIRAERRRLEEEELRRRGHLLSDGGTNALDALSQEGLSEEPVQQQEREAVGSGGGGTTWEVVAAAGGGRMKQRRRKKVVAAGREKRGGASAEEDEETEEGQEDDWNINDASGGISERQREHPFIVTEPGEVARGKKNGLDYLFHLYEQCRDFLIQVQNIAKERGEKCPTKVTNQVFRYAKKAGASYINKPKMRHYVHCYALHCLDEEASNALRRAFKERGENVGAWRQACYKPLVAIAARQGWDIDTIFNAHPRLAIWYVPTKLRQLCHSERSNAAAAAASSSVSGGGGGGDHLPHF

>Eschscholzia californica AAO49794.1 floricaula-like protein

MDPDAFTVGLYKWDPRGVVAPPPQPPPSNRLFEAIMTPTPMSYTIRPREYQQQQQIITGGIEDFFQAYGIRYYTAAKIAELGFTVSTLLDMKDEELDDMMNSIYHIFRWDLLVGERYGIKAAVRAERRRLEDDESRRRHLLTSETTTNPIDALSQEGLSEEPVQQEKEAAGSGGGGAWEVVLKEKKKIMKKKKQQRRRKQIEQGVADPEEDDDDDDEEEEDNNGGGGGGIERQREHPFIVTEPGEVARGKKNGLDYLFHLYEQCRDFLIQVQNIAKERGEKCPTKVTNQVFRFAKKAGASYINKPKMRHYVHCYAFHCLDEEASNALRKGFKERGENVGAWRQACYEPLVAMAARQGWDIDGIFNAHPRLSIWYVPTKLRQLCHAQRRTTTTNTTTVTTSSSVAALGAVTGPGHVF

>Gerbera hybrid cultivar ANS10152.1

MDPDAISASLFKWDPRAALAPPSSRLYEPITLQQPPPPPPPPPTMAVGSTATGGYLVRQSRDLGGLEEVFHAYGIRYLTATKIAELGFTANTLLDMKDEELDEMMNSLSHIFRWDLLVGERYGIKAAVRAERRRLDEEESRRRYILSSDATNTLDALSQEGLSEEPVQQENEAAGSGGGGAWDMAAMGGGGAGVKTTKQGHRRGKKTGLVRGRMGSSSQVGGDDNYENESDDDPENGGAGAGVVERQREHPFIVTEPGEVARGKKNGLDYLFHLYEQCREFLIQVQSIAKERGEKCPTKVTNQVFRFAKKAGASYINKPKMRHYVHCYALHCLDEDASNALRRAFKERGENVGAWRQACYKPLVTIAARQGWDIDAIFNTHPRLSIWYVPTKLRQLCHAERSSAAIVAAAASSSVAGGGGGGGHLHF

>Populus trichocarpa AAB51533.1 FLORICAULA/LEAFY homolog

MDPEAFTASLFKWDTRAMVPHPNRLLEMVPPPQQPPAAAFAVRPRELCGLEELFQAYGIRYYTAAKIAELGFTVNTLLDMKDEELDEMMNSLSQIFRWDLLVGERYGIKAAVRAERRRLDEEDPRRRQLLSGDNNTNTLDALSQEGFSEEPVQQDKEAAGSGGRGTWEAVAAGERKKQSGRKKGQRKVVDLDGDDEHGGAICERQREHPFIVTEPGEVARGKKNGLDYLFHLYEQCRDFLIQVQSIAKERGEKCPTKVTNQVFRYAKKAGASYINKPKMRHYVHCYALHCLDEDASNALRRAFKERGENVGAWRQACYKPLVAIASRQGWDIDSIFNAHPRLAIWYVPTKLRQLCYAERNSATSSSSVSGTGGHLPF

>Lotus japonicus AAX13294.1 transcription factor LFY

MDPDAFTASLFKWDPRTVLPTTGPLRPPLLDYAVAPPPAPAAVYHPARAPREIGGLEELFQAYGIRYYTAAKIAELGFTVSTLVDMKDDELDDMMNSLSQIFRWDLLVGERYGIKAAVRAERRRLDEEDMKRRNLLSTDTTTNALDALSQEGVNKFVGLSEEPVVQREKEVVGSGGGSTWEVAVVAEERRKKQRRRSRMKQHGDDNEEGEDEEGEDDDEGNNNSGGGGGCERQREHPFIVTEPGEVARGKKNGLDYLFHLYEQCREFLIQVQAIAKDRGEKCPTKVTNQVFRYAKKAGASYINKPKMRHYVHCYALHCLDEEVSNELRRGFKERGENVGAWRQACYKPLVAIAARQGWDIDAIFNAHPRLSIWYVPTKLRQLCHAERNSAAASSSVSVGTAHLPF

>Pinus radiata AAB51587.1 FLORICAULA/LEAFY-like protein

MDPESFSAAFFKWDQRPPALAPPQMQRSAGLEAQRIFHDFGVPNAAAMAASNNSSSCRKELNCLEELFRNYGVRYITLTKMVDMGFTVNTLVNMTEQELDDLVRTLVEIYRVELLVGEKYGIKSAIRAEKRRLEEAERKRMEQLFVDVDGKRKIDENALDTLSQEGLSVEEPQGDNAIILSQNNTSANFPLNLNAGMDPVLILQNSGHLGTTVSGLIGMPDTNYGSEQTKACKKQKRRRSKDSGEDGEERQREHPFIVTEPGELARGKKNGLDYLFDLYEQCGKFLLDVQHIAKERGEKCPTKVTNQVFRHAKHSGAGYINKPKMRHYVHCYALHCLDIEQSNRLRRAYKERGENVGAWRQACYYPLVAMAKDNGWDIEGVFNKHEKLRIWYVPTKLRQLCHLEKSKQSHL

>Pinus radiata AAB68601.1 FLORICAULA/LEAFY-like protein NDL

MDAEHFPVGFFRWDQRPAPVVAAAAAPTTTVFNKDHGRPLEVILPMNGRKDLKSLEDLFKEYGVRYVTLAKMTEMGFTANTLVNMTEEEIEDLMKTLVELYHMDLLIGERYGIKSAIRAEKKRLQDSLEMQRLEILSEAERKRILHDDQNTFAAAMASEGTSKELRANDPLIFPESTSADHAPMNIASCKDSTLILQNSNQAQFCGSGLIGVPEHSSESDERKADTNKQKRRRSKEPGEDGEDRPREHPFIVTEPGELARGKKNGLDYLFDLYEQCGKFLLEVQRIAKEKGEKCPTKVTNQVFRHAKHNGAVYINKPKMRHYVHCYALHCLDSEQSNHLRRLYKERGENVGAWRQACYYPLVAIARENNWDIEGIFNRNEKLKIWYVPTKLRQLCHMERSKECQ

>Picea abies AAV49504.1 LEAFY-like protein

MQRGGGLEAQRVFHDFGVPNAAAMTSNNSGSCRKELNCLEELFRHYGVRYITLTKMVDMGFTVNTLVNMTEQELDDLIRTLVDIYRVELLVGEKYGIKSAIRAEKRRLDEAERKRMEQLFVDVDGKRKIDENALDTLSQEGLSVEEPQGDNAIILSQNNTSASFPLNLNAGMDPVLILQNSCHLGTTVSGLIGMPDSNYGSEQTKGCKKQKRRRSKDSGEDGEERQREHPFIVTEPGELARGKKNGLDYLFDLYEQCGKFLLDVQHIAKERGEKCPTKVTNQVFRHAKHSGAGYINKPKMRHYVHCYALHCLDVEQSNRLRRAYKERGENVGAWRQACYYPLVAMAKDNGWDIEGVFNKHEKLRIWYVPTKLRQLCHFEKSKQSHL

>Picea abies AAV49503.1 NEEDLY-like protein, partial

MTEMGFTANTLVNMTEEEIEDLMKTLVEIYHMDLLIGERYGIKSAIRAEKKRLQDCLEMQRLEILSEAERKRILHDDQNTFAAAMTSEGTSKELRANDPLIFPESTSADHAPMNIASCKDSTLILQNSNQAQFCGSGLIGVPEHSSESDERKADTNKQKRRRSKEPGEDGEDRPREHPFIVTEPGELARGKKNGLDYLFDLYEQCGKFLLEVQRIAKEKGEKCPTKVTNQVFRHAKHNGAVYINKPKMRHYVHCYALHCLDSEQSNHLRRLYKERGENVGAWRQACYYPLVAIARENNWDIEGIFNRNEKLKIWYVPTKLRQLCHMERSKECP

>Matteuccia struthiopteris AAF77608.1 MatstFLO

MDPEQFPSSLFRWDQRVIPRKEVPSMEVPLLPAATNTKQLKLLEDLFKEYGVRSTTIIKIMEMGFTVSTLVNMMEQEIDDVIKTMMEGYHMELLVGEKYGLKSAIRAERKRQEEDMERQRLQLLAKNGKKHKSDDSGMVATSVEGTREQRGDNGMMFPDAVALNGPLNLNSKEHAQQEHSHAQFGPPGLLALPEPSSDNEGRKLARKKQKRRLSREPGEDGDDRTREHPFIVTEPGEVARGKKNGLDYLFDLYEQCARFLDEVQQMSRERGEKCPTKVTNQVFRHAKLKGASYINKPKMRHYVHCYALHCLDKEKSNLLRKLFKERGENVGAWRQACYYPLVDMARDNGWDIEGVFVRNEKLRIWYVPTKLRQLCHLEKSKDSDSCSYE

**Phytozome v12.1 (https://phytozome.jgi.doe.gov/pz/portal.html):**

>Marchantia polymorpha Mapoly0113s0034.1.p

MDPRLDHSRMDPRMDPRLDPRMEPRMDPRMDARSMDPRLEQRMDGRMMQPSADNRSFEFGFTTAGGRREVRTLEDLFHGYGVRIATLAKMTEMGFTVSTLVNMTEAELDDVIQTISDILQMELLVGERYGIKSAVRAEKKHLEEELERQRLELLTKSERKRKLDEVTSALVTKEGVFPILAHTLKKQKIFSRVSPTSSMTFVGEGSGDASPGTVSEQRRESAMMMPESIAPVNALNLNSKEPPLLMGAGHSSLTPGLLALAENSSDSEDKRLGKKKQKRRRPKEHGEDGEDRPREHPFIVTEPGEAARGKKNGLDYLFDLYEQCGKFLEQVQQLAREKGEKCPTKVTNQVFRHAKHTGAGYINKPKMRHYVHCYALHCLDIDQSNALRKLYKERGENVGAWRGACYYPLVAMARDNNWDIEGLFNRNEKLRIWYVPTKLRQLCHVEKVKYGE

>Spirodela polyrhiza32G0007500
MDPADEFSAAANPFRCWDLRMGHPNRLIEAAASLLAAQVEAAPPGRGELEEMFQAYGVRYQTVEKIGELGFTVSTLAAMREEELEDMMAALCHVFRWELLVGERYGIKAAVRTERRRRQLDGFFQLDEEEQRRRRHVVSPDSGALDALSQEGLSEERVQQEREAVASGGEAWEGHKKKKTNTSTRRNKKTTTKKKKNEEEEEDGEEEDDDDDEEEDGEGECEGKVPGCGAAAGGERQREHPFIVTEPGEVARGKKNGLDYLFHLYDQCRDFLLQVQSISRERGHKCPTKA

>Zostera marina27g00160.1
MDPSDAFSASMFRWDPRTAVGFYGGPPLTSPQQQQQQQHQQMQQVQQQQQQQQAAPISGGGGVVGRLVVVRELDEIFQSYGVRYATVERIRELGFTASTLVGMKDDEIDDMMGTLCHLFRWELLVGERYGIKAAVREERRRVDFTVDGRRNTGAAPASFFRGDSHYILDALSQEGLSEEPVQQDNEAAGSGGEVGGGGHKIKQKQRRGSSSKKSPTQQKPRKQKKVVVLEDDDEDIGDDEDDDGSKSGGCGGSSTGGGERQREHPFIVTEPGEVARGKKNGLDYLFHLFDQCREFLIQVQNQAKENGEKCPSKVTNQVFRYAKKSGASYINKPKMRHYVHCYALHCLDEEVSNELRRTFKERGENVGAWRLACYRPLVLIASHHAWDIDAVFAAHPRLAVWYVPTTLRQLCHHARSNAAVNVNATVSASGAAPPSSSVHHGGCAGNNGLPALF

>Aquilegia coerulea 5G327800.1
MDPEAFSAASLYKWERAAAAAAAPQHHHRLQFEPMALPPSPPPPQPSLPSYCVTRPPRELLLTLEDFFQPYGIRYTMIAKIAELGFTVSTLLDMRDEELDEMMITLSQIVRLDLLVGEKYGIKAAIRAERRRYHVLLGGGAAGGHVDNTTNPLDALSQGGLSEEPVMKEAAGSGGGTWDMVSAGEKRKKKNKAQRSRKARNSGDENYDDEDDEEDAAGERGCGRHRQREHPFVVTEPGEVARGKKNGLDYLFHLYEECRQYLIQVQNIAKDRGEKCPTKVTNQVYRHAIQAGATHINKPKIRHYVHCYALHCLDEEASNALRKAFKDRGENVGAWRQACYEPLVKFASDHHYDIDCIFNDHPRLSIWYVPTKLRKLCHAQRNNVASTSNSASF

**One Thousand Plants (1KP) project (https://db.cngb.org/blast/):**

>Isoetes_tegetiformans:PKOX_scaffold_2013584

RREAKCLEELFRDYGIRVSTLAKMTELGFTVSTLLNMTEQELEDIINAMVETYRIDLLVGERYGIKAAIRAERRLINEEAETQRLRSLCKSEKKRKQVEDALNEGAKENGNLFLGGSIYLDTDDPILTGQQSQSLTTGVLALREHSSESDGESHGKKKQKRRRPRELGDDGEDRPREHPFIVTEPGELARGKKNGLDYLFDLYEQCGRFLEEVQHICRERGEKCPTKVTNQVFRHAKHTGASYINKPKMRHYVHCYALHCLDTNHSNHLRKLFKDRGENVGAWRQACYYPLVELARANNWDIEGVFNRNDKLRIWYVPTRLRQLCHIEKSKE

>Selaginella_kraussiana:ZFGK_scaffold_2008343 tips of shoots

MDPEGSFPGALFRTWEPRMAPPPAVAAAAIHPSSGASSSSVAAAAAAAAPRIDPPPRAFEFGFASVRRESKTLDELFKDYGVRMSTLSKMTELGFTVQTLVNMTDQELEDVIKTMLESYHVELLVGEKYGIKSAIRAERKLIDDELERQRSKTQQQQQPKRRKPDEVVPSTTGTATTTAAVVSTIRDGSLKEPRRDSGSLAEIAPKEPNLVGQSSQVVVASSMPVESNGNGSDGERKRKKKQKRRRSKDDGEDGEDRPREHPFIVTEPGELARGKKNGLDYLFDLYEQCGKYLEEVQQQCKERGEKCPTKVTNQVFRHAKHRGASYINKPKMRHYVHCYALHCLDLEQSNHLRKLFKDRGENVGAWRQACYFPLVEMARSFNWDIEGVFSRNEKLRIWYVPTRLRQLCHLEKSKQ

>Sceptridium_dissectum:EEAQ_scaffold_2003960 sterile leaf

PTNRKQMKSLEELFKDYGVRITTIAKVVEMGFTVSTLVNMTEQEIDDVIKTILDGYHIELLVGERYGFKSAIRAEKRRHEEEVERQRLQLLSRSGKKRKTDETAVATTSLEGLQREQRADNDLMFPDAVAMVGPLNLNSKEPVLLEQNHTHFGAPALLAIPEPSSDNEKRKLGRKKQKRRLSREPGEDGDDRTREHPFIVTEPGELARGKKNGLDYLFDLYEQCARFLDEVQQISKERGEKCPTKVTNQVFRHAKLKGASYINKPKMRHYVHCYALHCLDLEQSNHLRKLYKDRGENVGAWRQACYYPLVEMARQNGWDIEGVFNRNEKLRIWYVPTKLRQLCHIEKSKD

>Equisetum_hymale:JVSZ_scaffold_2012838 sterile leaves/branches

PPPPTRSLDDLFQDYGVRAATVAKVAEMGFTVSTLVNMTELEIDDLVQALLDGFHPDLLVGEKYGLKSAIRAEKRRLEEDMNRQRLQILLKAGKKRAADESNKGAALEGQRVSREHQGDNDMGFADVVVSAGPLNLNSKEHVLLEQNRLNFGTPGLLSFPEPSSDNEGRKGGRKKKNRLLSQENGEDVDDRQREHPFIVTEPGELARGKKNGLDYLFSLYEQCAKFLEEVQQMSKERGEKCPTKTHGLQVTNQVFRHAKLKGASYINKPKMRHYVHCYALHCLDIEQSNHFRKLYKERGENVGAWRQACYYPLVEMARRNGWDIEGVFNRNEKLRIWYVPTKLRQLCHVEKSKD

>Equisetum_diffusum:CAPN_scaffold_2005353 tips of shoots

LEDLFQGYGVRAATIAKVAEMGFTVSTLVNMTEPEIDDLVQALLDDGFRHPPDNLLVGEKYGLKSAIRAQKRCLEEDMNRQRRSSSRLDDHKTSNGAGAGVSLAGGERVSREHQGDDDMGFADVVASVGPLNLNSKEHVLLEQNRLNFGTPGLLSFPEPSSDNEGRKGGRKKKSRLLSQENGEDVDDRQREHPFIVTEPGELARGKKNGLDYLFSLYEQCAKFLEEVQQMSKERGEKCPTKVTNQVFRHAKLKGASYINKPKMRHYVHCYALHCLDVEQSNHFRKLYKERGENVGAWRQACYYPLVEMARRNGWDIEGVFNRNEKLRIWYVPTKLRQLCHVEKSKD

>onekp:YOWV_scaffold_2068436 Cystopteris_protrusa sterile leaves

VPSMEAPLLPAATNTKQLKLLEDLFKEYGVRSTTIIKIMEMGFTVSTLVNMMEQEIDDVIKTMMEGYHMELLVGEKYGLKSAIRAERKRQEEDMERQRLQLLAKNGKKHKSDDSGMVATSVEGTREQRGDNGMMFPDPVALNGPLNLNSKDHTQQEHSHAQFGPPGLLALPEPSSDNEGRKLARKKQKRRLSREPGEDGDDRTREHPFIVTEPGEVARGKKNGLDYLFDLYEQCARFLDEVQQMSRERGEKCPTKVTNQVFRHAKLKGASYINKPKMRHYVHCYALHCLDKEKSNLLRKLFKERGENVGAWRQACYYPLVDMARDNGWDIEGVFVRNEKLRIWYVPTKLRQLCHLEKSKD

>onekp:AFPO_scaffold_2074266 Athyrium sp. gametophyte

PAATNTKQLKLLEDLFKEYGVRSTTIIKIMEMGFTVSTLVNMMEQEIDDVIKTMMEGYHMELLVGEKYGLKSAIRAERKRQEEDMERQRLQLLAKNGKKHKSDDSGMVATSVEGTREQRGDNGMMFPDAVALNGPLNLNSKEHAQQEHSHAQFGPPGLLALPEPSSDNEGRKLARKKQKRRLSREPGEDGDDRTREHPFIVTEPGEVARGKKNGLDYLFDLYEQCARFLDEVQQMSRERGEKCPTKVTNQVFRHAKLKGASYINKPKMRHYVHCYALHCLDKEKSNLLRKLFKERGENVGAWRQACYYPLVDMARDNGWDIEGVFVRNEKLRIWYVPTKLRQLCHLEKSKD

>onekp:RFRB_scaffold_2035413 Didymochlaena_truncatula young fronds

PAATNTKQLKLLEDLFKEYGVRSTTIIKIMEMGFTVSTLVNMMEQEIDDVIKTMMEGYHMELLVGEKYGLKSAIRAERKRQEEDMERQRLQLLAKNGKKHKSDDSGMVATSVEGTREQRGDNGMMFPDAVALNGPLNLNSKEHAQQEHSHAQFGPPGLLALPEPSSDNEGRKLARKKQKRRLSREPGEDGDDRTREHPFIVTEPGEVARGKKNGLDYLFDLYEQCARFLDEVQQMSRERGEKCPTKVTNQVFRHAKLKGASYINKPKMRHYVHCYALHCLDKEKSNLLRKIFKERGENVGAWRQACYYPLVDMARDNGWDIEGVFVRNEKLRIWYVPTKLRQLCHLEKSKD

>onekp:UFJN_scaffold_2002777 Diplazium_wichurae fertile leaf

PSATNTKQLKLLEDLFKEYGVRSTTIIKIMEMGFTVSTLVNMMEQEIDDVIKTMMEGYHMELLVGEKYGLKSAIRAERKRQEEDMERQRLQLLAKNGKKHKSDDSGMVATSVEGTREQRGDNGMMFPDAVALNGPLNLNSKEHAQQEHSHAQFGPPGLLALPEPSSDNEGRKLARKKQKRRLSREPGEDGDDRTREHPFIVTEPGEVARGKKNGLDYLFDLYEQCARFLDEVQQMSRERGEKCPTKVTNQVFRHAKLKGASYINKPKMRHYVHCYALHCLDKEKSNLLRKLFKERGENVGAWRQACYYPLVDMARDNGWDIEGVFVRNEKLRIWYVPTKLRQLCHLEKSKD

>onekp:VITX_scaffold_2098665 Blechnum_spicant young leaf

PAATNTKQLKLLEDLFKEYGVRSTTIIKIMEMGFTVSTLVNMMEQEIDDVIKTMMEGYHMELLVGEKYGLKSAIRAERKRQEEDMERQRLQLLAKNGKKHKSDDSGMVATSVEGTREQRGDNGMMFPDAVALNGPLNLNSKEHAQQENSHAQFGPPGLLALPEPSSDNEGRKLARKKQKRRLSREPGEDGDDRTREHPFIVTEPGEVARGKKNGLDYLFDLYEQCARFLDEVQQMSRERGEKCPTKVTNQVFRHAKLKGASYINKPKMRHYVHCYALHCLDKEKSNLLRKLFKERGENVGAWRQACYYPLVDMARDNGWDIEGVFVRNEKLRIWYVPTKLRQLCHLEKSKD

>onekp:FQGQ_scaffold_2001052 Polystichum_acrostichoides ster & fert leaves

PAATNTKQLKLLEDLFKEYGVRSTTIIKIMEMGFTVSTLVNMMEQEIDDVIKTMMEGYHMELLVGEKYGLKSAIRAERKRQEEEMERQRLQLLAKNGKKHKSDDSGMVATSVEGTREQRGDNGMMFPDAVALNGPLNLNSKEHAQQEHSHAQFGPPGLLALPEPSSDNEGRKIARKKQKRRLSREPGEDGDDRTREHPFIVTEPGEVARGKKNGLDYLFDLYEQCARFLDEVQQMSRERGEKCPTKVTNQVFRHAKLKGASYINKPKMRHYVHCYALHCLDKEKSNLLRKIFKERGENVGAWRQACYYPLVDMARDNGWDIEGVFVRNEKLRIWYVPTKLRQLCHLEKSKD

>onekp:YJJY_scaffold_2006838 Woodsia_scopulina young sterile leaves

PAATNTKQLKLLEDLFKEYGVRSTTIIKIMEMGFTVSTLVNMMEQEIDDVIKTMMEGYHMELLVGEKYGLKSAIRAERKRQEEDMERQRLQLLAKNGKKHKSDDSGMVATSVEGTREQRGDNVMMFPDAVALNGPLNLNSKEHAQQEHSHAQFGPPGLLALPEPSSDNEGRKLARKKQKRRLSREPGEDGDDRTREHPFIVTEPGEVARGKKNGLDYLFDLYEQCARFLDEVQQMSRERGEKCPTKVTNQVFRHAKLKGASYINKPKMRHYVHCYALHCLDKEKSNLLRKLFKERGENVGAWRQACYYPLVDMARDNGWDIEGVFVRNEKLRIWYVPTKLRQLCHLEKSKD

>onekp:MROH_scaffold_2048633 Thelypteris_acuminata young leaf

PASSNTKQLKLLEDLFKEYGVRSTTIIKIMEMGFTVSTLVNMMEQEIDDVIKTMMEGYHMELLVGEKYGLKSAIRAERKRQEEDMERQRLQLLAKNGKKHKSDDSGMVATSVEGTREQRGDNGMMFPDAVALNGPLNLNSKEHAQQEHSHAQFGPPGLLALPEPSSDNEGRKLARKKQKRRLSREPGEDGDDRTREHPFIVTEPGEVARGKKNGLDYLFDLYEQCARFLDEVQQMSRERGEKCPTKVTNQVFRHAKLKGASYINKPKMRHYVHCYALHCLDKEKSNLLRKLFKERGENVGAWRQACYYPLVDMARDNSWDIEGVFVRNEKLRIWYVPTKLRQLCHLEKSKD

>onekp:JBLI_scaffold_2100752 Bolbitis repanda leaf

PTATSTKQLKLLEDLFKEYGVRSTTIIKIMEMGFTVSTLVNMMEQEIDDVIKTMMEGYHMELLVGEKYGLKSAIRAERKRQEEDMERQRLQLLAKNGKKHKSDDSGMVATSVEGTREQRGDNGMMFPDAVALNGPLNLNSKEHAQQEHSHAQFGPPGLLALPEPSSDNEGRKIARKKQKRRLSREPGEDGDDRTREHPFIVTEPGEVARGKKNGLDYLFDLYEQCARFLDEVQQMSRERGEKCPTKVTNQVFRHAKLKGASYINKPKMRHYVHCYALHCLDKEKSNLLRKIFKERGENVGAWRQACYYPLVDMARDNGWDIEGVFVRNEKLRIWYVPTKLRQLCHLEKSKD

>onekp:HNDZ_scaffold_2012192 Cystopteris_utahensis mostly sterile leaf

PAATNTKQLKLLEDLFKEYGVRSTTIIKIMEMGFTVSTLVNMMEQEIDDVIKTMMEGYHMELLVGEKYGLKSAIRAERKRQEEDMERQRLQLLAKNGKKHKSDDSGMVATSVEGTREQRGDNGMMFPDPVALNGPLNLNSKDHTQQEHSHVQFGPPGLLALPEPSSDNEGRKLARKKQKRRLSREPGEDGDDRTREHPFIVTEPGEVARGKKNGLDYLFDLYEQCARFLDEVQQMSRERGEKCPTKVTNQVFRHAKLKGASYINKPKMRHYVHCYALHCLDKEKSNLLRKLFKERGENVGAWRQACYYPLVDMARDNGWDIEGVFVRNEKLRIWYVPTKLRQLCHLEKSKD

>onekp:HEGQ_scaffold_2080968 Gymnocarpium_dryopteris young sterile leaves

PAATNTKQLKLLEDLFKEYGVRSTTIIKIMEMGFTVSTLVNMMEQEIDDVIKTMMEGYHMELLVGEKYGLKSAIRAERKRLEEDMERQRLQLLAKNGKKHKSDDSGMVATSVEGTREQRGDNGMMFPDPVALNGPLNLNSKDHTQQEHSHAQFGPPGLLALPEPSSDNEGRKLARKKQKRRLSREPGEDGDDRTREHPFIVTEPGEVARGKKNGLDYLFDLYEQCARFLDEVQQMSRERGEKCPTKVTNQVFRHAKLKGASYINKPKMRHYVHCYALHCLDKEKSNLLRKLFKERGENVGAWRQACYYPLVDMARDNGWDIEGVFVRNEKLRIWYVPTKLRQLCHLEKSKD

>onekp:WGTU_scaffold_2004634 Leucostegia_immersa young leaves

PAATNTKQLKLLEDLFKEYGVRSTTIIKIMEMGFTVSTLVNMMEQEIDDVIKTMMEGYHMELLVGEKYGLKSAIRAERKRQEEEMERQRLQLLVAKNGKKHKSDDSGMVATSVEGTREQRGDNGMMFPDAAALNGPLNLNSKEHAQQEHSHAQFGPPGLLALPEPSSDNEGRKLARKKQKRRLSREPGEDGDDRTREHPFIVTEPGEVARGKKNGLDYLFDLYEQCARFLDEVQQMSRERGEKCPTKVTNQVFRHAKLKGASYINKPKMRHYVHCYALHCLDKEKSNLLRKIFKERGENVGAWRQACYYPLVDMARDNGWDIEGVFVRNDKLRIWYVPTKLRQLCHLEKSKD

>onekp:ZQYU_scaffold_2008636 Polypodium_plectolens leaf

PATTNTKQLKLLEELFKEYGVRSTTIIKIMEMGFTVSTLVNMMEQEIDDVIKTMMEGYHMDLLVGEKYGLKSAIRAEKKRQEEDMERQRLQLLAKNGKKHKSDDSGMVATSVEGTREQRGDNGMMFPDAVAHNGPLNLNSKEHAQQEHSHAQFGPPGLLALPEPSSDNEGRKSARKKQKRRLSREPGEDGDDRTREHPFIVTEPGEVARGKKNGLDYLFDLYEQCARFLDEVQQMSRERGEKCPTKVTNQVFRHAKLKGASYINKPKMRHYVHCYALHCLDKEKSNLLRKIFKERGENVGAWRQACYYPLVDMARDNGWDIEGVFVRNEKLRIWYVPTKLRQLCHLEKSKD

>onekp:MTGC_scaffold_2008394 Dennstaedtia ?

PATTNTKQLKLLEDLFKDYGVRSTTIIKVMEMGFTVSTLVNMMEQEIDDVIKTMMEGYHMDLLVGEKYGLKSAIRAERKRQEEDMERQRLQLLAKNGKKHKSDDSGMVATSVEGTREQRGDNGMMFPDAVALNGPFNLNSKEHAQQENSHAHTQFGPPGLLALPEPSSDNEGRKLGRKKQKRRLSREPGEDGDDRTREHPFIVTEPGEVARGKKNGLDYLFDLYEQCARFLDEVQQMSRERGEKCPTKVTNQVFRHAKLKGASYINKPKMRHYVHCYALHCLDKEKSNLLRKLFKERGENVGAWRQACYYPLVDMARDNGWDIEGVFVRNDKLRIWYVPTKLRQLCHLEKSKD

>onekp:GANB_scaffold_2069908 Cyathea_spinulosa leaves

PPAATNSKQLKLLEDLFKDYGVRTTTIIKVMEMGFTVNTLVNMMEQEIDDVIKTMMEGYHMELLVGEKYGLKSAIRAEKKRQEEEMERQRLQLLAKNGKKHKSDDSGMVATSAEGTREQRGDNGMMFPNAVAANGPLNLNSKEHAQQEHSHAQFGPPGLLAIPEPSSDNEGRKLARKKQKRRLSREPGEDGDDRTREHPFIVTEPGEVARGKKNGLDYLFDLYEQCARFLDEVQQISRERGEKCPTKVTNQVFRHAKLKGASYINKPKMRHYVHCYALHCLDKEKSNFLRKMFKERGENVGAWRQACYYPLVEMARDNGWDIEGVFVRNEKLRIWYVPTKLRQLCHLEKSKD

>onekp:ORJE_scaffold_2018095 Phymatosorus glossus leaf

PATTNNKQLKLLEELFKEYGVRTTTVIKIMEMGFTVSTLVNMMEQEIDDVIKTMMEGYHMDLLVGEKYGLKSAIRAEKKRQEEDMERQRLQLLAKNGKKHKSDDSGMVATSVEGTREQRGDNGMMFPDAVAHNGPLNLNSKEHAQQEHSHAQFGPPGLLALPEPSSDNEGRKSARKKQKRRLSREPGEDGDDRTREHPFIVTEPGEVARGKKNGLDYLFDLYEQCARFLDEVQQMSRERGEKCPTKVTNQVFRHAKLKGASYINKPKMRHYVHCYALHCLDKEKSNLLRKIFKDRGENVGAWRQACYYPLVDMARGNGWDIEGVFVRNEKLRIWYVPTKLRQLCHLEKSKD

>onekp:UWOD_scaffold_2025982 Plagiogyria_japonica young leaves

PAATNSKQLKLLEDLFKDYGVRTATIIKVMEMGFTVSTLVNMMEQEIDDVIKTMTEGYHMELLVGEKYGLKSGIRAEKKRQEEEMERQRLQLLAKSGKKHKSDDSGMVATSVEGTREQRGDNGMMFPDAVAANGPLNLNSKEHGQQEHSHAQFGPPGLLALPEPSSDNEGRKLARKKQKRRLSREPGEDGDDRTREHPFIVTEPGEVARGKKNGLDYLFDLYEQCARFLDEVQQMSRERGEKCPTKVTNQVFRHAKLKGASYINKPKMRHYVHCYALHCLDKEKSNFLRKLFKERGENVGAWRQACYYPLVDMARDNGWDIEGVFVRNEKLRIWYVPTKLRQLCHVEKSKD

>onekp:WQML_scaffold_2068699 Cryptogramma_acrostichoides ster & fert leaves

PPTITNTKQLKLLEDLFKDYGVRSTTIIKVMEMGFTVSTLVNMMEQEIDDVIKTMLEGYHMELLVGEKYGLKSAIRAERKRQEEEMERQRLQLLAKNSKKNKSDDSGMVATSAEGTREQRGDNGMMFPDAVAPNGPLNLNSKEHAQQENSHAQFGPPGLLALPEPSSDNEGRKLARKKQKRRLSREPGEDGDDRTREHPFIVTEPGEVARGKKNGLDYLFDLYEQCARFLDEVQQMSRERGEKCPTKVTNQVFRHAKLKGASYINKPKMRHYVHCYALHCLDREKSNFLRKQFKERGENVGAWRQACYYPLVDMARDNGWDIEGVFVRNEKLRIWYVPTKLRQLCHLEKSKD

>onekp:WCLG_scaffold_2004512 Adiantum_aleuticum young sterile leaves

PPATNNTKQLKLLEDLFKDYGVRSTTIIKVMEMGFTVSTLVNMMEQEIDDVIKTMMEGYQMELLVGEKYGLKSAIRAERKRQEEEMERQRLQLLAKNTKKHKSDDSGMVATSAEGTREQRGDNGMMFPDAVAPNGPLNLNSKEHPQQEHSHAHFGPPGLLALPEPSSDNEGRKLARKKQKRRLSREPGEDGDDRTREHPFIVTEPGEVARGKKNGLDYLFDLYEQCARFLDEVQQMSRERGEKCPTKVTNQVFRHAKLKGASYINKPKMRHYVHCYALHCLDKEKSNFLRKQFKERGENVGAWRQACYYPLVDMARDNGWDIEGVFVRNEKLRIWYVPTKLRQLCHLEKSKD

>onekp:PSKY_scaffold_2058265 Asplenium_nidus leaf

PPAGTSTKQLKLLEDLFKDYGLRNTTIIKIMEMGFTVNTLVNMMEQEIDDVIKTMMDGYHMELLVGEKYGLKSAIRAERKRQEEDMEWQRLQLLAKNGKKHKLDDSGMVATSVEGTREQRGDNGMMFPDAAALNGPLNLNMKEHAQQEHSHAQFGPPGLLALPEPSSDNEGRKLARKKQKRRLSREPGEDGDDRTREHPFIVTEPGEVARGKKNGLDYLFDLYEQCARFLEEVQQMSRERGEKCPTKVTNQVFRHAKLKGASYINKPKMRHYVHCYALHCLDKEKSNLLRKLFKERGENVGAWRQACYYPLVDMARDNCWDIEGVFVRNEKLRIWYVPTKLRQLCHLEKSKD

>onekp:ZXJO_scaffold_2016630 Hemionitis_arifolia leaf

PPAANNTKQLKLLEDLFKDYGVRSTTIIKVMELGFTVSTLVNMMEQEIDDVIKTMMEGYQMELLVGEKYGLKSAIRAERKRQEEEMERQRLQLLAKNSKKHKSDDSGMVATSAEGTREQRGDNGMMFPDAGAPNGPLNLNSKEHPQQEHSHAHFGPPGLLALPEPSSDNEGRKLARKKQKRRLSREPGEDGDDRTREHPFIVTEPGEVARGKKNGLDYLFDLYEQCARFLDEVQQMSRERGEKCPTKVTNQVFRHAKLKGASYINKPKMRHYVHCYALHCLDKEKSNFLRKQFKERGENVGAWRQACYYPLVDMARDNGWDIEGVFVRNEKLRIWYVPTKLRQLCHLEKSKD

>onekp:GSXD_scaffold_2012608 Myriopteris_eatonii young leaves

PPAANNTKQLKLLEDLFKDYGVRSTTIIKVMEMGFTVSTLVNMMEQEIDDVIKTMMEGYQMELLVGEKYGLKSAIRAERKRQEEEMERQRLQLLAKSSKKHKSDDSGMVATSAEGTREQRGDNGMMLPDAVAPNGPLNLNSKEHPQQEHSHAHFGPPGLLALPEPSSDNEGRKLARKKQKRRLSREPGEDGDDRTREHPFIVTEPGEVARGKKNGLDYLFDLYEQCARFLDEVQQMSRERGEKCPTKVTNQVFRHAKLKGASYINKPKMRHYVHCYALHCLDKEKSNFLRKQFKERGENVGAWRQACYYPLVDMARDNGWDIEGVFVRNEKLRIWYVPTKLRQLCHLEKSKD

>onekp:DCDT_scaffold_2016115 Cheilanthes_arizonica sterile leaves

PPAANNSKQLKLLEDFFKDYGVRSTTIIKVMEMGFTVSTLVNMMEQEIDDVIKTMMEGYQMELLVGEKYGLKSAIRAERKRQEEEMERQRLQLLAKNSKKHKSDDSGMVATSAEGTREQRGDNGMMFPDAGAPNGPLNLNSKEHPQQEHSHAHFGPPGLLALPEPSSDNEGRKLARKKQKRRLSREPGEDGDDRTREHPFIVTEPGEVARGKKNGLDYLFDLYEQCARFLDEVQQMSRERGEKCPTKVTNQVFRHAKLKGASYINKPKMRHYVHCYALHCLDKEKSNFLRKQFKERGENVGAWRQACYYPLVEMARDNGWDIEGVFVRNEKLRIWYVPTKLRQLCHLEKSKD

>onekp:YCKE_scaffold_2064612 Notholaena_montieliae young leaves

PPAANNSKQLKLLEDLFKDYGVRSTTIIKVMEMGFTVSTLVNMMEQEIDDVIKTMIEGYQMELLVGERYGLKSAIRAERKRQEEEMERQRLQLLAKNSKKHKSDDSGMVATSAEGTREQRGDNGMMFPDAGAPNGPLNLNSKEHPQQEHSHAHFGPPGLLALPEPSSDNEGRKLARKKQKRRLSREPGEDGDDRTREHPFIVTEPGEVARGKKNGLDYLFDLYEQCARFLDEVQQMSRERGEKCPTKVTNQVFRHAKLKGASYINKPKMRHYVHCYALHCLDKEKSNFLRKQFKERGENVGAWRQACYYPLVDMARDNGWDIEGVFVRNEKLRIWYVPTKLRQLCHLEKS

>onekp:KJZG_scaffold_2004358 Asplenium_platyneuron leaves

KQLKLLEDLFKDYGVRNTTIVKIMEMGFTVNTLVNMMEQEIDDVIKTMMEVYHMELLVGEKYGLKSAIRAERKRQEDDVERQRLQLLAKNGKKHKSDDSGMVATSVEGTREQRGDNVMMFPDAVALNGPLNLNTKEHTQQEHSHAQFGPPGLLALPEPSSDNEGRKLARKKQKRRLSREPGEDGDDRTREHPFIVTEPGEVARGKKNGLDYLFDLYEQCARFLEEVQQMSRERGEKCPTKVTNQVFRHAKLKGASYINKPKMRHYVHCYALHCLDKEKSNLLRKLFKERGENVGAWRQACYYPLVDMARDNCWDIEGVFVRNEKLRIWYVPTKLRQLCHLEKSKD

>onekp:POPJ_scaffold_2101632 Pteris_vittata fronds

PPTTTNAKQLKLLEDLFKDYGVRSTTILKVMEMGFTVNTLVNMMEQEIDDVIKTMMDGYHMELLVGEKYGLKSAIRAERKRQEEEMERQRLQLLAKNSKKHKSDDSGMVATSVEGTREQRGDNGMMFPDAVAPNGPLNLNSKEQQENSHAQFGPPGLLALPEPSSDNEGRKLARKKQKRRLSREPGEDGDDRTREHPFIVTEPGEVARGKKNGLDYLFDLYEQCARFLDEVQQMARERGEKCPTKVTNQVFRHAKLKGASYINKPKMRHYVHCYALHCLDKEKSNFLRKQFKERGENVGAWRQACYYPLVEMARDNGWDIEGVFVRNEKLRIWYVPTKLRQLCHLEKSKD

>onekp:YIXP_scaffold_2001880 Lindsaea_microphylla young fronds & sori

PLPAVPNPNSVPNSNSKQLKLLEDLFKDYGVRSTTIVKVIEMGFTVNTLVNMMEQEIDDVITTMTEGYHMELLVGEKYGLKSAIRAEKKRQEEDLDRQRLQLMAKNGKKHRSDDSGMVVTSVEGTREQRGDNGMMFPDAVALGDPLNLNSREHVQQEHSHAQFGPPGNLALPEPSSDNEGRKLARKKQKRRLSREPGEDGDDRTREHPFIVTEPGEVARGKKNGLDYLFDLYEQCARFLDEVQQISRERGEKCPTKVTNQVFRHAKVKGASYINKPKMRHYVHCYALHCLDKEKSNLLRKLFKERGENVGAWRQACYYPLVDMARDNGWDIEGVFVRNEKLRIWYVPTKLRQLCHLEKSKD

>onekp:NOKI_scaffold_2011879 Lindsaea_linearis young fronds & sori

PLPAVPNPNSIPNPNSKQLKLLEDLFKDYGVRSTTIVKVMEMGFTVNTLVNMMEQEIDDVITTMTEGYHMELLVGEKYGLKSAIRAEKKRQEEDLDRQRSQLMAKNGKKHRSDDSGMVVTSVEGTREQRGDNGMMFPDAVALGDPLNLNSREHVQQEHSHAQFGPPGNLALPEPSSDNEGRKLARKKQKRRLSREPGEDGDDRTREHPFIVTEPGEVARGKKNGLDYLFDLYEQCARFLDEVQQISRERGEKCPTKVTNQVFRHAKLKGASYINKPKMRHYVHCYALHCLDKEKSNLLRKLFKERGENVGAWRQACYYPLVDMARDNGWDIEGVFVRNEKLRIWYVPTKLRQLCHLEKSKD

>onekp:FLTD_scaffold_2017634 Pteris_ensigormis young leaves

KQLKLLEDLFKDYGVRSTTILKVMEMGFTVNTLVNMMEQEIDEVIKTMMEGYHMELLVGEKYGLKSAIRAERKRQEEEMERQRLQLLAKNSKKHKSDDSGMVATSAEGTREQRGDNGMMFTDAAAPNGPLNLNSKEQQEHSHAQFGPPGLLALPEPSSDNEGRKLARKKQKRRLSREPGEDGDDRTREHPFIVTEPGEVARGKKNGLDYLFDLYEQCARFLDEVQQMSRERGEKCPTKVTNQVFRHAKLKGASYINKPKMRHYVHCYALHCLDKEKSNFLRKQFKERGENVGAWRQACYYPLVEMARDNGWDIEGVFVRNEKLRIWYVPTKLRQLCHLEKSKD

>onekp:XDDT_scaffold_2004989 Argyrochosma_nivea young leaves

KQLKLLEDLFKDYGVRSTTIIKVMEMGFTVSTLVNMMEQEIDDVIKTMMEGYQMELLVGEKYGLKSAIRAERKRQEEEMERQRLQLLSSKKHKSDDSGMVATSAEGTREQRGDNGMMLPDAVAPNGPLNLNSKEHPQQEHSHAHFGPPGLLALPEPSSDNEGRKLARKKQKRRLSREPGEDGDDRTREHPFIVTEPGEVARGKKNGLDYLFDLYEQCARFLDEVQQMSRERGEKCPTKVTNQVFRHAKLKGASYINKPKMRHYVHCYALHCLDKEKSNFLRKQFKERGENVGAWRQACYYPLVDMARDNGWDIEGVFVRNEKLRIWYVPTKLRQLCHLEKSKD

>onekp:BMJR_scaffold_2008901 Adiantum_tenerum leaf

PPTTNNSKQLKLLEDLFKDYGVRSTTIIKVMEMGFTVSTLVNMTEQEIDDVIKTMIEGHQMGLLVGEKYGLKSAIRAERKRQEEEMERQRMQLLGAKNSKNHKSDDSGMVATSAEGTREQRGESGMILPDAAAPKGPLNLNSKEHPQQEHSHAHFGPPGLLALPEPSSDNEGRKLARKKQKRRLSREPGEDGDDRTREHPFIVTEPGEVARGKKNGLDYLFDLYEQCARFLDEVQQMSRERGEKCPTKVTNQVFRHAKLKGASYINKPKMRHYVHCYALHCLDKEKSNFLRKQFKERGENVGAWRQACYYPLVEMARDNGWDIEGVFVRNEKLRIWYVPTKLRQLCHLEKSKD

>onekp:UJTT_scaffold_2010715 Pityrogramma_trifoliata leaf (maybe sporangia)

PPSTANAKQLKLLEDLFEDYGVRSTTILKVMEMGFTVNTLVNMMEPEIDDVIKTMVDGYHIELLVGEKYGLKSAIRAERKRQEEETERQRLQVLAKSSKKHKSDDSGLVATSFEGAREQRGDNGMMFPDAVAPNGPLNLNSKEQQEHSQALFGPPGLLALPEPSSDNEGRKLARKKQKRRLSREPGEDGDDRTREHPFIVTEPGEVARGKKNGLDYLFDLYEQCARFLDEVQQMARERGEKCPTKVLVTNQVFRHAKLKGASYINKPKMRHYVHCYALHCLDKEKSNCLRKQFKERGENVGAWRQACYYPLVEMARDNGWDIEGVFVRNEKLRIWYVPTKLRQLCHLEKSKD

>onekp:SKYV_scaffold_2001904 Vittaria_lineata leaf, mostly sterile

SKLLEDLFKDYGVRSTTIMKVMEMGFTVSTLINMMEQEIDDVIKTMIEVYQMELLVGEKYGLKSAIRAERKRQEDETERQRLLLLAKNSKRHKSDDSGMVATSGEGTREQRGDNGMMLPDAVAPSGPLNLNSKDHPQQEHSHAHFGPPGLLVLPEPSSDNEGCKVTRKKQKRRLSREPGEDGDDRTREHPFIVTEPGEVARGKKNGLDYLFDLYEQCGRFLDEVQQMSRERGEKCPTKVTNQVFRHAKLKGASYINKPKMRHYVHCYALHCLDKEKSNFLRKQFKERGENVGAWRQACYYPLVDMARDNGGGIESIFIRNEKLRIWYVPTKLRQLCHLERSKD

>onekp:PBUU_scaffold_2012780 Lygodium_japonicum sterile & fertile leaves

PPLFPSNNKQLKSLEDLFKDYGVRITTIVKILEMGFTVNTLVNMTELEIDDVIKTMIEGFQMELLVGERYGMKSAIRAERKRQEEELERQRLQILSKNSKKQKPDDSVMAATSVEGSREQRGDNGIMFPDTLAVTGPLNLNSKEHLLPEQSPAKFGPPGLMALPEPSSDNEGRKLVRKKQKRRLSREPGEDGDDRTREHPFIVTEPGEVARGKKNGLDYLFDLYEQCARFLDEVQQISRERGEKCPTKVTNQVFRHAKLKGASYINKPKMRHYVHCYALHCLDREKSNYLRKLFKERGENVGAWRQACYYPLVEMARENGWDIEGVFIRNEKLRIWYVPTKLRQLCHLEKSKD

>onekp:CQPW_scaffold_2003265 Anemia_tomentosa sterile leaf

PPTPASSNKQLKSLEDLFKDYGVRVATIVKVLEMGFTVNTLVNMTEQEIDDVIKTMIEAFHTELLVGERYGMKSAIRAERKRQEEELERQRLVILSKTGKNQKPDDSVLAATSMEGSREQRGDNAMMFSDAIVVNGPLNLNSKEHLLQSHAKFGPPGLMALPEPSSDNEGRKIARKKQKRRLSREPGEDGDDRTREHPFIVTEPGEVARGKKNGLDYLFDLYEQCARFLDEVQQISRERGEKCPTKVTNQVFRHAKLKGASYINKPKMRHYVHCYALHCLDREKSNYLRKLFKERGENVGAWRQACYYPLVEMARENGWDIEGVFIRNEKLRIWYVPTKLRQLCHLEKSKD

>onekp:UOMY_scaffold_2080585 Osmunda_sp. gametophyte

PSNRKQMKSLEDLFKDYGVRLTTIAKVMELGFTVNTLVNMTEQEIDDVIKTMLEGYRIELLVGEKYGLKSAIRAEKKRQEEEMERQRMQLLSKSGKKRKPDDSAMVATSMDGTREQRGDNGMMFPDAVAAVGPLNLNSKEPLLLEQSHAQFGTPGLLAIPEPSSDNEGRKLGRKKQKRRLSREPGEDGDDRPREHPFIVTEPGEVARGKKNGLDYLFDLYEQCARFLDEVQQISKERGEKCPTKVTNQVFRHAKLKGASYINKPKMRHYVHCYALHCLDLEKSNHLRKLFKERGENVGAWRQACYYPLVDMARENGWDIEGVFNRNEKLRIWYVPTKLRQLCHQEKSKD

>onekp:CVEG_scaffold_2031619 Azolla_cf._caroliniana sterile leaves

PVTPTHSKQIKLLEDLFKDYGVRAATIVKFMEMGFTFNTLVNMIEPEIDDVIKTMVEGYQMELLVGERYGLKSAIRAEKKRQDEEVERQRLKTLGNSNGKKQKLDDNGTAVTSAEGNREQRGDNGIMFLDAVATNGPLNLNSREHAQQEHSHAQLGPPGLLAIPEPSSDNEGRTLVRKKQKRRLSSREPGEDGDDRTREHPFIVTEPGEVARGKKNGLDYLFDLYEQCGRFLDEVQQMSRERGEKCPTKVTNQVFRHAKLKGASYINKPKMRHYVHCYALHCLDKEKSNFLRKLYKERGENVGAWRQACYYPLVEMARANGWDTEGVFVRNEKLRIWYVPTKLRQLCHLEKSKD

>onekp:VIBO_scaffold_2005768 Osmunda_javanica mature leaves

PSNRKQMKSLEDLFKDYGVRLTTIAKVMELGFTVNTLVNMTEQEIDDVIKTMLEGYRIELLVGEKYGLKSAIRAEKKRQEEDMERQRMQLLSKSCKKRKPDDSAMVATSMDGTREQRGDNGMMFPDAVAAVGPLNLNSKEPLLLEQSHAQFGTPGLLAIPEPSSDNEGRKLGRKKQKRRLSREPGEDGDDRPREHPFIVTEPGEVARGKKNGLDYLFDLYEQCARFLDEVQQISKERGEKCPTKVTNQVFRHAKLKGASYINKPKMRHYVHCYALHCLDLEKSNHLRKLFKERGENVGAWRQACYYPLVDMARENGWDIEGVFNRNEKLRIWYVPTKLRQLCHQEKSKD

>onekp:MEKP_scaffold_2108290 Dipteris_conjugata young leaves

KQLKSLEDLFKDYGVRLTTIAKVMEMGFTVSTLVNMTEQEIEDVIKTMLEGYHIDLLVGEKYGFKSAIRAEKKRQDEEMEQQRLQLLSKSGKKRQPDEIATSMEGTREQRGDNEMMFPDAVAAGGPLNSKEAVLLEQSHAQFGPPGLLAIPEPSSDNDGRKLGKKKQKRRLSREPGEDGDDKPREHPFIVTEPGEVARGKKNGLDYLFDLYEQCARFLDEVQQISKERGEKCPTKVTNQVFRHAKLKGASYINKPKMRHYVHCYALHCLDLEKSNHLRKMFKERGENVGAWRQACYYPLVEMARENGWDIEGVFNRNEKLRIWYVPTKLRQLCHQEKSKD

>onekp:CVEG_scaffold_2125709 Azolla_cf._caroliniana sterile leaves

LEDLFKDYGVRTSTTVKFLEMGFTVNTLVNMTEPEIDDVITTMMEGYQMELLVGEKYGLKSAIRAEKKRQEEEIEQQRLQCLGKNGKKHKLDEYSGIASTSAEGTREQRGDNGMMMLDAIATDGPLNLNSNEHLQQEHIHSGFSLPGVLPMPEPSSDDNEEQKFARKKQKRRLLREPGGEDGDDRTREHPFIVTEPGEVARGKKNGLDYLFDLYEQCASFLDEVQQISRERGEKCPTKVTNQVFRHAKLKGASYINKPKMRHYVHCYALHCLDKEKSNFLRKLYKERGENVGAWRQACYYPLVEMARTNGWDIEGVFVRNEKLRIWYVPTKLRQLCHLEKSKD

>onekp:KIIX_scaffold_2012644 Pilularia_globulifera young leaves

PIPNPNPNSNSKQLKLLEDLFKDYGVRVTTIVKFLEMGFTVSTLVNMTEQEIDDVIKTMTEGFQIELLVGEKYGLKSAIRAEKRRQEEEVERQRLQLLSKSKKHKSDDSGMVVTSAEGTREQRGDNAMMFLDAGVTNGPLNLNSKEHTQQDHSHAQFGPPGLLAIPEPSSDNEGRKLARKKQKRRLSREPGEDGDDRTREHPFIVTEPGEVARGKKNGLDYLFDLYEQCARFLDEVQQISRERGEKCPTKVTNQVFRHAKLKGASYINKPKMRHYVHCYALHCLDKEKSNFLRKLYKERGENVGAWRQACYYPLVEMA

>onekp:EWXK_scaffold_2021182 Thyrsopteris_elegans young leaves

RRRQQKSLEDLFRDYGVRLSTVAKVMEMGFTVSTLVNMTEDEIEDVINKSMVEVELLVGERYGLKSAIRAEKRRQEDEERQRFGRDRKGRKFDDIATSMEESIPIPIHLDLLIFESVNALHSHSVYWNYSAGFSAGTREQGGDNGGMLADAVATVGPFLNVNLKERVQLEQRHAQFGPLGQLAIPEPCSDNDGRQLGRKNKKQRLSQQRGEDGDDRTREHPFIVTEPGEVARGKKNGLDYLFDLYEQCARFLDEVQQMAKERGEKCPTKVTNQVFRHAKMKGASYINKPKMRHYVHCYALHCLDLEKSNHLRKLFKERGENVGAWRQACYSPLVKMARETGWDIEGVFMRNEKLRIWYVPTKLRQLCHQEKSKD

>onekp:URCP_scaffold_2006393 Athyrium_filix_femina young leaf

PAATNTKQLKLLEDLFKEYGVRSTTIIKIMEMGFTVSTLVNMMEQEIDDVIKTMMEGYHMELLVGEKYGLKSAIRAERKRQEEDMERQRLQLLAKNGKKHKSDDSGMVATSVEGTREQRGDNGMMFPDAVALNGPLNLNSKEHAQQEHSHAQFGPPGLLALPEPSSDNEGRKLARKKQKRRLSREPGEDGDDRTREHPFIVTEPGEVARGKKNGLDYLFDLYEQCARFLDEVQQMSRERGEKCPTKV

>onekp:NWWI_scaffold_2031159 Nephrolepis_exaltata young leaf

EYGVRSTTIIKIMEMGFTVSTLVNMMEQEIDDVIKTMTEGYHMELLVGEKYGLKSAIRAERKRQEEEMERQRLQLLAKNSKKQIDSGMVATSVEGTREQRGDNGMMFPDAVALNGPLNLNSKEHAQQEHSHAQFGPPGLLALPEPSSDNEGRKLARKKQKRRLSREPGEDGDDRTREHPFIVTEPGEVARGKKNGLDYLFDLYEQCARFLDEVQQMSRERGEKCPTKVTNQVFRHAKLKGASYINKP

>onekp:XXHP_scaffold_2011093 Cystopteris_fragilis fronds

VALNGPLNLNSKDHTQQEHSHAQFGPPGLLALPEPSSDNEGRKLARKKQKRRLSREPGEDGDDRTREHPFIVTEPGEVARGKKNGLDYLFDLYEQCARFLDEVQQMSRERGEKCPTKVTNQVFRHAKLKGASYINKPKMRHYVHCYALHCLDKEKSNLLRKLFKERGENVGAWRQACYYPLVDMARDNGWDIEGVFVRNEKLRIWYVPTKLRQLCHLEKSKD

>onekp:FCHS_scaffold_2007396 Deparia_lobato fertile leaf

AVALNGPLNLNSKEHAQQEHSHAQFGPPGLLALPEPSSDNEGRKLARKKQKRRLSREPGEDGDDRTREHPFIVTEPGEVARGKKNGLDYLFDLYEQCARFLDEVQQMSRERGEKCPTKVTNQVFRHAKLKGASYINKPKMRHYVHCYALHCLDKEKSNLLRKLFKERGENVGAWRQACYYPLVDMIEGVFVRNEKLRIWYVPTKLRQLCHLEKSKD

>onekp:NDUV_scaffold_2012951 Vittaria_appalachiana gametophyte

AVAPSDPLNLNSKDNIQQEHSLARFGPSGLMVLPEPSSDNEGRKLARKKQKRRLSREPGEDGDDRTREHPFIVTEPGEVARGKKNGLDYLFDLYEQCARFLDEVQQMSRERGEKCPTKVTNQVFRHAKLKGASYINKPKMRHYVHCYALHCLDKEKSNFLRKQFKERGENVGAWRQACYYPLVDMARDNGWDIEGVFVRNEKLRIWYVPTKLRQLCHLERSKD

>onekp:PNZO_mergedscaffolds_2024395_2026047_Culcita_macrocarpa young leaves

PAATNSKQLKLLEDLFKDYGVRMATIIKVMEMGFTVSTLVNMMEQEIDDVIKTMTEGYHMELLVGEKYGLKSGIRAEKKRQEEEMERQRLQLLAKSGKKHKSDDSGMVATSVEGTREQRGDNGMMFPDAVAANGPLNLNSKEHAQQEHSHAQFGPPGLLALPEPSSDNEGRKLTRKKQKRRLSREPGEDGDDRTREHPFIVTEPGEVARGKKNGLDGEKCPTKVTNQVFRHAKLKGASYINKPKMRHYVHCYALHCLDKEKSNFLRKLFKERGENVGAWRQACYYPLVDMARDNGWDIEGVFVRNEKLRIWYVPTKLRQLCHVEKSKD

>onekp:QIAD_scaffold_2007026 Hymenophyllum_bivalve young fronds

RKKQKRMLSREPGEDGDDRPREHPFIVTEPGEVARGKKNGLDYLFDLYEQCARFLDEVQQISKERGEKCPTKVTNQVFRHAKLKGASYINKPKMRHYVHCYALHCLDLEKSNTLRKLFKERGENVGAWRQACYYPLVDMARENGWDIEGVFIRNEKLRIWYVPTKLRQLCHQEKSKD

>onekp:TRPJ_scaffold_2004493 Hymenophyllum_cupressiforme young fronds &sori

RKKQKRRPSREPGEDGDDRPREHPFIVTEPGEVARGKKNGLDYLFDLYEQCARFLDEVQQISKERGEKCPTKVTNQVFRHAKLKGASYINKPKMRHYVHCYALHCLDLEKSNTLRKLFKERGENVGAWRQACYYPLVDMARENGWDIEGVFVRNEKLRIWYVPTKLRQLCHQEKSKD

>onekp:OQWW_scaffold_2029676 Davallia_fejeensis leaf

LDPEQFPSSLYRWDQRVIPRKEVPSMEVPLLPATTNTKQLKLLEDLFKEYGVRGTTIIKIMEMGFTVSTLVNMMEQEIDDVIKTMMEGYHMDLLVGEKYGLKSAIRAEKKRQEEDMERQRLQLL

>onekp:HTFH_mergedscaffolds_2000526_2035984_Onoclea_sensibilis leaves

DRTREHPFIVTEPGEVARGKKNGLDYLFDLYEQCARFLDEVQQRVTNQVFRHAKLKGASYINKPKMRHYVHCYALHCLDKEKSNLLRKLFKERGENVGAWRQACYYPLVDMARDNGWDIEGVFVRNEKLRIWYVPTKLRQLCHLEKSKD

>onekp:UJWU_scaffold_2067330 Pleopeltis_polypodioides dehydrating fronds

KQLKLLEDLFKEYGVRSTTIIKIMEMGFTVSTLVNMMEQEIDDVIKTMMEGYHMDLLVGEKYGLKSAIRAEKKRQEEDMERQR

**Genome sequence**

> *Thalictrum thalictroides* Tt_Genome_WT478-4-170_contig_108877_consensus

TTCAGAAAACAAATTGGTTTATTAATCTTATCCAACAATGGAAATTGATTATATTATCATTCATCTTGTTACCATATTATCCAAATGCCACAGAGAAGTAATTTTCCTGTTCAGTAATTCTCCGAAATTGGTTTACTAATCAAAATCTTATTTTTGTCCTGTTCAGTAATTTTCCGAAATTAATTACAATGGCCACAGAGAAGTAGAGAACCCATCTCTGTGACCGGCCACAGAATACCCGGACTCCTAAAAAGAACTGCTGAGAAACCAATTAATAATCTCTTGCCAGTTGCCACTGACCATTGTGGTGAGAGTAAATCTGCATGCAGGAGAGAAAGTACAGATAAGGTATCATGCAGAGATGCTCCAGGCGTGAATCAAACCCTAGCTGTCCAGCTAACTCAAAGCAGAGCTATCAAACCATCTGAAAAAGGCTGTTAGTTTTTGTACTTTCAAACCAATCCTATCTCTGATCTCTCTGCTGCTACCTATACAATACAACATGGATCTATTTTGTACATATCTCGAGGTGTGGTCTATCAATGTTTTGGAAGTCAACACGTATTTCTGATGGTTCAGCTATGCTAGCTTTTAGTACATAATACTAGCTGAGTTGTGTAGGCACTTCACTTATCCACAATACTGTATATATATATAAATATCACAGTTTTGAATGCGCCTTTGGTACAATGCTATGGTTGTTGGTACTTGGTAGTGTATTGATCGAGCATTTCAGCTATTGGCATTGCACCAGTGGTTCAACCATGCTGATGAGTCCATAAGACCATGATGACCCTAGCTCCAACATGATGTCATCAAGAATACAATACATAGTACTACAGTAACTAGTAGTAATAAACTGCAACCAAGTTCATTTTTACAACATTGAATTGGAAAATGGAAATGCATTCCCAAATCCCAACCATTACTTATCATCTAACCATGCAAAAATCACATATCCTCTAGCTTGTGGCATGTTGTAATTTTCACTAAAACCAAGGTCAGCTTAATGTTCAATTCATTATTCACCCTAATTACTTATTAGCAAGCAGGTTCGTTGAATGAGCTAGAAATAAAGCTAGATCTGAAGAGAAACAAGCAAGAAAGACCACACACACATACACAAACCCTGCAGACAGATCGACTCTAATTTGTTGTAGCTAGCCAATGGATTCAGAACCATTCTCAACAAGCTTATACAAGTGGGACACAAGAGCAACAGCAGCAGCAGCTGCACCACACCGCTTGCAGTTTGAACATATGGCTATTGCACCACCACCACCACCACAACAACCATCATCATTTCCACCTTATTGTGTGACAACCAGACCACCAGCAAGAGAACTACTTTTAACTCTTGAAGACTTCTTTCAGCCATATGGCATTCGTTACACTATGATTGCAAAAATAGCTGAGTTAGGCTTCACTGTAAGCACACTTTTAGACATGAAAGATGAAGAGCTTGATGATATGATGATTACTCTTTCTCAAATAGTTCGGATTGATTTACTTGTTGGTGAAAAATATGGTATCAAAGCTGCTATTCGTGCTGAACGTCGTCG

>*Thalictrum thalictroides* Tt_Genome_WT478-4-170_contig_116935_consensus

AAGAAGATGATGAGAATGCTGGAGCTGGACGTGGTCGTTGTCATAGACAAAGAGAGCATCCATTTGTAGTTACTGAACCAGGTGAAGTTGCAAGAGGAAAAAAAAATGGTCTTGATTACTTGTTCCATCTCTATCAAGAATGTAGTCAATACTTGATTCAAGTTCAAAACATTGCTAAGGATAGAGGTGAAAAATGCCCAACCAAGGTATGAATGTATATATGATCATCACTTAGTTTTACAATTTTTTTTTTTTTNNNNNNNNNNNNNNNNNNNNNNNNNNNNNNNNNNNNNNNNNNNNNNNNNNNNNNNNNNNNNNNNNNNNNNNNTAAAAAAAAAACCAGATAAAAATTGTACTATTAAACAGGTAGGAAAAGTTGTTACAAAATATCTATGAAAAATGTCAGATGGAAGTATTTAAGACACAAGTCATTCTTATATCCAACAGTCATTTTTTATCACTATAGTTCAGACTTCAGAGTTCAGACCAATCTAGGGCGAATTTGTCAAAACCACACTCGAACATTGTGTAATTTGCATGAACCCTATCAAAAGAAATAAATTTTTGGTGTTCTTAATATCTGCAACATTTTTTTTTGTCAAACCATTATCTGACACTACAAATTGCAGTTTAATCTGCTTGTAAAATGTAGAGAAATGATAACAATCCGGCCATCTTATTCGATGGTATATATGCATATATATATATGCAGGTGACGAATCAGGTGTATCGACATGCAATTCAGGCTGGTGCAACTCATATCAACAAGCCTAAAATACGACATTATGTTCACTGTTATGCTCTGCACTGCCTAGATGAACAAGTATCTAATGCGCTGAGGAAAGCTTACAAAGACCGAGGTGAAAATGTTGGGACATGGAGGCAAGCATGCTATGAACCTCTTGTGAAATTGGCTAGTGATCATCACTACGATATTGATGCCATTTTTAATGATCATCCCAGCCTTTCTATTTGGTATGTACCTACAAAGCTTCGTAAGCTCTGTCATGCTCAACGCAACAATGTTGTCTCTGCTTCAAACTCAGCTTCATTCTGATCTAGCTAGTCTAGCTCCTTATGAGTGGTCAGTGAGGTTATGTTATTGTAGTACTATATTTGAATATTTTCGTGTTTTCGTANNNNNNNNNNNNNNNNNNNNNNNNNNNNNNNNNNNGACAGCACTGAATGTTAACTTTGCCATGCTATTGTAGTATATTTGTATCATGGAACTTCAATTGGTTTACACAAACACATTCCACACACCTGATTGCAAAACCCTAATATAATTAGATCTTTCAGCTTAACATATTGATGATACTTCAGGATTATGAATACATCCACCAAAAAATCCTGTTCAAATTTCAAAATAACCCAGTTAAACGCAAATAAATCACTGATCAGTAATCAATAGAACTACACAATTATCATGTCCATGCCCCCTCTTGAGCCTGGAGTGAGCCAAATCAAAGTCAAAGAGAATGTTTCAACGATTTTCCTGCAGCAAGAAATAAAATAGAATCTACTACACTGATTAGATACTTCTGATTATTGTATATAGGAATAGGTTACGATTGAACTTGATCGAAATAAAAATGTGTTTTGCAATCTGATCCACATTTCTTAGCACCTAACTAGACTATAAGCAAGCCTAACACAGGTTTCTTCCAGGCAAACTGCACTCCTGCAACTAGCACATAACATGTTCAATTGACTTCTAAATAGTTGCTCCCAGCTGAAAACTGAGTCCTTAAACTTCATGATGAAAACAGATAACAACCTTAGATCGAAGGCCTTCCAATATCTCTACATTGATTCTAGGCAATCTTTGCATCTACCTAATGGCACATTTGCATCCTCCATGAAGGTTGGTCCATGGACAGTTGTTTTTATTAGGGTATTCTATGCTTATCATGCAATAGATCTTAAAAGTTAAGTCTCATCACATTAAGACTATCACGGCAACTCTGTGAGATCCAGACAAAACCTTTCCATCGAGAGAAGTTTCAATGCAAAACCCGATTTGTGAATGAAAACAAACATTATGACAATTTTACAAAAGACTCCATATGAACTTGCTATTTTGTTCATTAATGCCCAGAATGTCTCTTTGTCCAAAAGTATAAAGGGTATGGTAAATCTTACCTCAACAAAGTAACAACATTGCCCATGAGATACGGAGACAGTGTAATTACAATTTGAGTAGACGCAGTCGTTTTAGACAATGTGGTTCAAAAGAATTCACCCTGAGGGAGAACAAACTCTTTATTATTTTCTTTACTAGTATAAATATATACTGAGTTGGAGTTCCTTATGTCTAGTAGAACATATCTTGGGCAGGCAATGCATGATACCATGGCTGGCGTTGATCTGTTCATCCCTATGTATTGCAAACAGAAATGATTAATAAGTCAAAAGAAACTATCCAACTAGAATATAACTATGTGCAAAGCAAGGCGACAAGAAAAAGGGTACTGCCGGTATTGCTAGAACATATTGGCTTATTACTAGTACTTGGCCATTTGTAGCATGAATTACTTTATCTCAGGGAAAATTAAGTTGCCTAATAAAACACAAAGGCACTTGATATCGATGGAAGTAGCCCATAATAAAGTTACAGGGTTATCTCATATATATGTGCTAGTAATGTGCTTACTGAAGTGCCATGTTTATGGTAAAAGAATATGTGATAAACAGTATCTCCTTTGTTCATCCACCTGGAGAGAAGCTAACTCTGCTGCGGATGCCCTTGCAAACAAAGGTGCAGATTTAGGAGTTGATGTTAGGGAAAATTTTGTTAGTAGGCCACCATTCCTTCAAAGGGTGGAGGACCCACATAGCTGTTACTTTAGGTTTAGGTTTAAGGACTAGTATTTTGTGTACCGTCCTCACATTGTTTTCCTTTTTCCTGTTTTCCTTTTTCTTTCTCTTTGTAAATCTTTTCTTCCACAGTTTAATAAAATTGCAACTTATCAAGGAAAAAAAAAAGTATCTCCTTTGAAAAACTCCCATTTTTATTATGAGTTAGAAGTTCTATGTTGGCCTAACCCCTATATAATTGATGTTCATTCTTCAATTTTCTTCCACGTTAAAAGAATTACGTTCAAGGCCAGTATACCCCCAAGATGAAAGGAAACAAAGCAATTCCCCAACCCCCCGCGGCAACACCAGTAAAGTAAGTATAGCAATATATTCAGAGGTAACTTCTTCGTCTTCAATAATATACGCAAATAGATGTCTCTTTTCTTTCTTAGGGAAGTCAGTTTCCAAACGCCCAACCTCATTGTAGTTGTAACATTTGTGCCCTGGATTAAAACCACTTGGGAGCAGAATTGATGGCTGTAGGAAGTAAAGGATATTGT

>*Thalictrum thalictroides* Predicted LFY Protein

MDSEPFSTSLYKWDTRATAAAAAPHRLQFEHMAIAPPPPPQQPSSFPPYCVTTRPPARELLLTLEDFFQPYGIRYTMIAKIAELGFTVSTLLDMKDEELDDMMITLSQIVRIDLLVGEKYGIKAAIRAERR-----------------------------------------------EDDENAGAGRGRCHRQREHPFVVTEPGEVARGKKNGLDYLFHLYQECSQYLIQVQNIAKDRGEKCPTKVTNQVYRHAIQAGATHINKPKIRHYVHCYALHCLDEQVSNALRKAYKDRGENVGTWRQACYEPLVKLASDHHYDIDAIFNDHPSLSIWYVPTKLRKLCHAQR
