# Supplementary material for: LEAFY maintains apical stem cell activity during shoot development in the fern Ceratopteris richardii
Source: eLife. 2018 Oct 24;7:e39625. doi: 10.7554/eLife.39625 (PMC6200394; doi:10.7554/eLife.39625)
Supplement: Supplementary file 4. [file elife-39625-supp4.docx]

**Supplementary File 4.** **Statistical comparison of *CrLFY* transcript levels between different ontogenetic stages.**

**Table S4-1. Pairwise comparisons of *CrLFY1* and *CrLFY2* transcript levels between ontogenetic samples.** Separate statistical comparisons were made between ontogenetic samples for *CrLFY1* and *CrLFY2*, each utilizing one-way ANOVA (*CrLFY1*, p < 0.0001; *CrLFY2*, p < 0.0001) followed by pairwise comparisons using Tukey’s multiple comparisons test (see Materials and Methods). Sample ontogeny is coded as **A** = imbibed spores, pre-germination; **B** = male gametophytes 5 days post spore sowing (DPS); **C** = mixed gametophytes 5 DPS; **D** = mixed gametophytes 8 DPS; **E** = fertilized gametophyte (gametophyte plus developing embryo); **F** = whole sporophytes including shoot apex and three simple fronds; **G** = sporophyte shoots including shoot apex and 5 simple fronds; **H** = frond, 3-5 lobes; **I** = complex vegetative frond (94 DPS); **J** = complex vegetative frond (114 DPS); **K** = reproductive (spore-bearing) frond. Representative images of each ontogenetic sample are shown in **Figure 3A-K**. *CrLFY1* transcript levels in sporophytes with three or five-fronds were significantly different from levels in all other samples (at a minimum significance of p < 0.01) and not significantly different between these two samples (p > 0.05). *CrLFY1* transcript levels were not significantly different (p > 0.05) between any other pair of samples. *CrLFY2* transcript levels in germinating spores were significantly different to all other samples (at a minimum significance of p < 0.01) and not significantly different between any other pair of samples. ns (not significant) = p > 0.05; * = p < 0.05; ** = p < 0.01; *** = p < 0.001; **** = p < 0.0001

**a.** *CrLFY1*

|  | **A** | **B** | **C** | **D** | **E** | **F** | **G** | **H** | **I** | **J** | **K** |
| --- | --- | --- | --- | --- | --- | --- | --- | --- | --- | --- | --- |
| **A** |  |  |  |  |  |  |  |  |  |  |  |
| **B** | ns |  |  |  |  |  |  |  |  |  |  |
| **C** | ns | ns |  |  |  |  |  |  |  |  |  |
| **D** | ns | ns | ns |  |  |  |  |  |  |  |  |
| **E** | ns | ns | ns | ns |  |  |  |  |  |  |  |
| **F** | **** | *** | *** | **** | ** |  |  |  |  |  |  |
| **G** | **** | **** | **** | **** | *** | ns |  |  |  |  |  |
| **H** | ns | ns | ns | ns | ns | *** | **** |  |  |  |  |
| **I** | ns | ns | ns | ns | ns | **** | **** | ns |  |  |  |
| **J** | ns | ns | ns | ns | ns | **** | **** | ns | ns |  |  |
| **K** | ns | ns | ns | ns | ns | **** | **** | ns | ns | ns |  |

**b.** *CrLFY2*

|  | **A** | **B** | **C** | **D** | **E** | **F** | **G** | **H** | **I** | **J** | **K** |
| --- | --- | --- | --- | --- | --- | --- | --- | --- | --- | --- | --- |
| **A** |  |  |  |  |  |  |  |  |  |  |  |
| **B** | *** |  |  |  |  |  |  |  |  |  |  |
| **C** | *** | ns |  |  |  |  |  |  |  |  |  |
| **D** | *** | ns | ns |  |  |  |  |  |  |  |  |
| **E** | **** | ns | ns | ns |  |  |  |  |  |  |  |
| **F** | **** | ns | ns | ns | ns |  |  |  |  |  |  |
| **G** | **** | ns | ns | ns | ns | ns |  |  |  |  |  |
| **H** | *** | ns | ns | ns | ns | ns | ns |  |  |  |  |
| **I** | ** | ns | ns | ns | ns | ns | ns | ns |  |  |  |
| **J** | *** | ns | ns | ns | ns | ns | ns | ns | ns |  |  |
| **K** | **** | ns | ns | ns | ns | ns | ns | ns | ns | ns |  |

**Table S4-2. Pairwise comparisons between *CrLFY1* and *CrLFY2* expression levels within each ontogenetic sample.** Statistical analysis was performed using two-way ANOVA, which identified a significant interaction between ontogenetic sample and *CrLFY* gene copy on the observed changes in transcript level (p < 0.0001). Subsequent pairwise comparisons were made between *CrLFY1* and *CrLFY2* transcript levels within each sample using Sidak’s multiple comparisons tests (see Materials and Methods). ns (not significant) = p > 0.05; * = p < 0.05; ** = p < 0.01; *** = p < 0.001; **** = p < 0.0001.

| Ontogenetic sample | *CrLFY1* vs *CrLFY2* |
| --- | --- |
| A (Imbibed spores, pre-germination) | ns |
| B (Male gametophytes 5DPS) | ns |
| C (Mixed gametophytes 5 DPS) | ns |
| D (Mixed gametophytes 8 DPS) | * |
| E (Fertilized gametophyte) | ns |
| F (Whole sporophyte, 3 simple fronds) | *** |
| G (Sporophyte shoot, 5 simple fronds) | **** |
| H (Frond, 3-5 lobes) | ns |
| I (Complex vegetative frond 94 DPS) | * |
| J (Complex vegetative frond 114 DPS) | * |
| K (Reproductive frond) | **** |
