# Supplementary material for: LEAFY maintains apical stem cell activity during shoot development in the fern Ceratopteris richardii
Source: eLife. 2018 Oct 24;7:e39625. doi: 10.7554/eLife.39625 (PMC6200394; doi:10.7554/eLife.39625)
Supplement: Supplementary file 5. — Alignment (prepared using Clustal Omega) of the full length transcript sequences for CrLFY1 and CrLFY2, with nucleotide identity between the two gene copies denoted by a subtending asterisk. The CDS for each gene is highlighted in bold. The target sequences for each RNAi construct are highlighted in light blue (CrLFY1/2-i1), light green (CrLFY1/2-i2), dark blue (CrLFY1-i3) or dark green (CrLFY2-i4). The CrLFY1/2-i1 and CrLFY1/2-i2 target sequences each have 77% similarity to the opposing gene transcript (BLAST2n, discontiguous megablast for highly similar sequences). The full-length CrLFY1-i3 and CrLFY2-i4 target sequences do not demonstrate significant sequence similarity to the opposing gene transcript or 3’UTR alone (BLAST2n, blastn for somewhat similar sequences) but short regions of similarity within the target sequence might explain the cross-reactivity observed. [file elife-39625-supp5.docx]

**Supplementary File 5. Specificity of *CrLFY* RNAi target sequences.**

Alignment (prepared using Clustal Omega) of the full-length transcript sequences for *CrLFY1* and *CrLFY2*, with nucleotide identity between the two gene copies denoted by a subtending asterisk. The CDS for each gene is highlighted in bold. The target sequences for each RNAi construct are highlighted in light blue (*CrLFY1/2-i1*), light green (*CrLFY1/2-i2*), dark blue (*CrLFY1-i3*) or dark green (*CrLFY2-i4*). The *CrLFY1/2-i1* and *CrLFY1/2-i2* target sequences each have 77% similarity to the opposing gene transcript (BLAST2n, discontiguous megablast for highly similar sequences). The full-length *CrLFY1-i3* and *CrLFY2-i4* target sequences do not demonstrate significant sequence similarity to the opposing gene transcript or 3’UTR alone (BLAST2n, blastn for somewhat similar sequences) but short regions of similarity within the target sequence might explain the cross-reactivity observed.

*CrLFY1* GTGCGAGGCATACACACACGCAGTCCTGGTGTGCCTTTCCTGTTCTAGTACTCATACAAA 60

*CrLFY2* ------------------------------------------------------------ 0

*CrLFY1* TACGACGTGCGCTTCCTTTTCTTCCTACCTTTGCTTTCAAATTGACGCATCTTCTGGTCT 120

*CrLFY2* --------------------------------------------------------AGCT 4

**

*CrLFY1* TCACTGCTTGTGAAGCAGGTTGTCACAAAAAGTTCCTGCTGCTTAT----GTTTACTCCA 176

*CrLFY2* CGCGAGAAGCAAGGGCTGGTTGTTACGGATAGCGGGCACAATTTAGGGTTGCACGCAGCA 64

* ** ****** ** * ** * *** * * **

*CrLFY1* GCGGTTCTTGTAATTGTGTCACCACAGCTGCTACCCGGAGTTACTCTGCAGTATCGACGC 236

*CrLFY2* GGTGATTCTGAATTTGTATCATCATCGTTGGAGTGCGGATTCACTGTGCAATAGTAGATT 124

* * * ** * **** *** ** * ** **** * *** **** **

*CrLFY1* AATTCTCCTT--------CTACGTAGCAGAAGCAGTGCATGACCTTCGATATTGTTCGTT 288

*CrLFY2* CTCCCTCCTTTACAGGTTTCCTGGAACGACAACGGATCATAACCATCGGGTTCTTTCGGT 184

****** * * * * * * *** *** *** * **** *

CrLFY1 TACACAGGTGAAGTTTGGCCTTCTCGGAAATTACAGACTATCTCCTCGTTCTCAAATGAA 348

CrLFY2 T---------------------------------------------------------AA 187

* **

CrLFY1 AAGCGGAGTTTTATCACT---TCACTGAAGTCGGGCATTGTCCGTGAAAGTCTCACCGAC 405

CrLFY2 CGAAGGAATTTGGTCGGAAGCTCTGTATCAGACATCCTAGTCACTGTTCTCATCCCTCTT 247

*** *** ** ** * * * *** ** ** *

CrLFY1 CGAAAGAAGTCGATCATTTCTTGTACTGCTCTCTATCACTTCAAGCTGGATCCTGAACAG 465

CrLFY2 TGGTCTTCCTCCTCCACTAAAGGTGCTGCTATCAACCAGATCAAGCTGGATCCTGAGCAC 307

* ** ** * ** ***** ** * ** **************** **

CrLFY1 TTTGCTAGTTCTTTGTTCCGGTGGGAACAAAGGGCTATCCCACGTAAAGAAGTGCCTCCA 525

CrLFY2 TTTCCTGGTCCC**ATGTTCCGATGGGAACAAAGGGTAATACATCGTAAGGAGGTCCCCTCT** 367

*** ** ** * ******* ************* ** * ***** ** ** ** *

CrLFY1 **ATGGATGTCTCTTTATTGCCACCAACCACCACCA------------------------TC** 561

CrLFY2 **ATGGAGGCTTCCATACTTCCTCCAACCACCACCACCACCACTGCTGCTACCTCTGTGACA** 427

***** * ** ** * ** *************

CrLFY1 **GCTGGTACTGCAGATCCGAAACAACTGAAACTTCTTGAAGATCTCTTTAAAGACTATGGA** 621

CrLFY2 **ACTGTTGGGACGTATCCAAAACAGCTTAAACTCCTTGAAGATCTCTTCAAGGACTATGGT** 487

*** * * **** ***** ** ***** ************** ** ********

CrLFY1 **GTACGAAGCACCACAATAATTAAGGTCATGGAGATGGGTTTCACTGTGAATACTTTGGTG** 681

CrLFY2 **GTACGGAGCACTACCATAGTGAAGGTTATGGAAATGGGTTTCACTGTTAGTACTCTAGTG** 547

***** ***** ** *** * ***** ***** ************** * **** * ***

CrLFY1 **AATATGATGGAGCAAGAGATTGATGACCTAATCAAGACCATGACAGAAAGCTACCATATG** 741

CrLFY2 **AACATGATGGAGCAAGAGATTGATGATGTTATCAAGACAATGATAGAGGGTTATCATATG** 607

** *********************** * ******** **** *** * ** ******

CrLFY1 **GAGTTGTTGGTCGGAGAGAAGTATGGCTTGAAGTCCGCTATTCGTGCAGAGAAGAAGCGG** 801

CrLFY2 **GAACTTCTGGTTGGTGAAAAATATGGTTTGAAATCTGCAGTCCGGGCTGAGAGAAAACGC** 667

** * **** ** ** ** ***** ***** ** ** * ** ** **** ** **

CrLFY1 **CAAGAAGAGGATATGGAACGCCAACGATTACAACTGCTAGCAACAAGTAGCAAAAAACAT** 861

CrLFY2 **CAAGAAGAGGAGATGGAACGTCAACGACTGCAGTTATTGGTAAAAAACAGCAAAAAGCTC** 727

*********** ******** ****** * ** * * * ** ** ******** *

CrLFY1 **AAGTCAGATGAAAGCGGCATGGTTGTAACTTCACTTGAAGGCACAAGGGAACAAAGAGGA** 921

CrLFY2 **AAGTCAGATGATAGTGGCATGATAGCAGTGTCTGTAGAAGGCACCAGGGAACAAAGAGGA** 787

*********** ** ****** * * * ** * ******** ***************

CrLFY1 **GATAATGTTATGATGTTTCCAGAGGCTGTTGCACCCAATGCCCCCTTGAATTTGAACTCG** 981

CrLFY2 **GACAATGGTATGATGTTTCCAGATACTGCTGCTCAGAATGGTCCCTTGAATCTGAACTCA** 847

** **** *************** *** *** * **** ********* *******

CrLFY1 **AAAGATCATGTCCAACAAGAGCATAGCCATGCACAAATAGGGCCACCTGGACTCCTGGCT** 1041

CrLFY2 **AAAGATCATGCTCAGCATGAGCATAGCCATGGCCTGTTTGGACCACCTGGACTCCTTGCT** 907

********** ** ** ************* * * ** ************** ***

CrLFY1 **CTACCAGAACCAAGCAGTGACAATGAGGGTCACAAATTACCAAGAAAAAAGCCGAAACGG** 1101

CrLFY2 **CTACCTGAACCTAGCAGTGACAATGAAGGACGCCAAATACCAAGGAAAAAGCAAAAACGA** 967

***** ***** ************** ** * * ** ******* ******* *****

CrLFY1 **AGGCTGTTGCGAGAACCTGGTGAGGACGGTGATGACAGAACAAGAGAGCATCCATTCATT** 1161

CrLFY2 **AGGTTATCGCGTGAGCCTGGTGAGGACGGGGATGACAGGACCAGAGAACACCCATTTATA** 1027

*** * * *** ** ************** ******** ** ***** ** ***** **

CrLFY1 **GTGACAGAGCCAGGTGAAGTGGCAAGGGGAAAGAAGAATGGTTTAGACTACTTGTTTGAT** 1221

CrLFY2 **GTGACAGAGCCTGGTGAAGTTGCAAGGGGAAAGAAAAATGGCTTAGATTATTTGTTTGAC** 1087

*********** ******** ************** ***** ***** ** ********

CrLFY1 **TTATACGAACAATGTGCACGCTTTTTAGATGAAGTGCAACAGATGGCCAGAGAAAGAGGG** 1281

CrLFY2 **CTGTATGAGCAGTGTGCACGATTCTTAGATGAAGTACAACAGATGGCAAGGGAAAGGGGG** 1147

* ** ** ** ******** ** *********** *********** ** ***** ***

CrLFY1 **GAGAAGTGTCCTACGAAGGTAACAAATCAAGTATTCCGGCATGCCAAGTTGAAAGGTGCG** 1341

CrLFY2 **GAGAAATGTCCTACAAAGGTAACAAACCAAGTGTTTCGACATGCCAAGCTCAAAGGTGCA** 1207

***** ******** *********** ***** ** ** ********* * ********

CrLFY1 **AGCTATATTAACAAGCCAAAGATGAGACACTATGTACACTGCTATGCACTGCATTGTCTG** 1401

CrLFY2 **AGTTATATCAACAAACCAAAGATGAGGCACTATGTTCACTGCTATGCCCTACATTGTCTG** 1267

** ***** ***** *********** ******** *********** ** *********

CrLFY1 **GACAAAGAGAAATCAAACTTTTTGAGAAAACAGTTCAAAGAGAGGGGAGAAAATGTGGGT** 1461

CrLFY2 **GATAAAGATAAATCAAACTTCTTAAGGAAACAGTTTAAAGAAAGAGGAGAGAATGTGGGT** 1327

** ***** *********** ** ** ******** ***** ** ***** *********

CrLFY1 **GCATGGCGACAAGCATGCTATTATCCATTGGTGGATATGGCTCGAGATAACGGCTGGGAT** 1521

CrLFY2 **GCATGGAGACAAGCTTGCTACTTCCCTCTGGTTGACATGGCTAGAGACAATGGTTGGGAT** 1387

****** ******* ***** * ** **** ** ****** **** ** ** ******

CrLFY1 **ATAGAGGGTGTCTTTGCGAGGAATGAGAAGCTCCGTATCTGGTATGTTCCTACAAGGCTT** 1581

CrLFY2 **ATAGAAGGTGTCTTTGTCAGAAATGAGAAGCTTCGGATTTGGTATGTCCCAACCAAACTT** 1447

***** ********** ** *********** ** ** ******** ** ** * ***

CrLFY1 **CGACAGCTCTGTCATTTAGAGAAGAGCAAGGACAGTGATAGCTGCATCTATGATTGA**AAT 1641

CrLFY2 **CGCCAACTTTGTCATTTTGAAAAGAGCAAGGACAGTGACAGCTGCAGCTATGAATAA**GTA 1507

** ** ** ******** ** ***************** ******* ****** * *

CrLFY1 TGGCTTCATTACT-GCACTTTGTAATATGTGGATCAAGCACACGACTGCATCATGATATC 1700

CrLFY2 CATACCTGAGGTGTTGTTTTTGTAATTTATCAATTCAATGCACG----TTTCACCACAGG 1563

******** * * ** * **** *** * *

CrLFY1 CCCGTATCATGATAATGCCTATGATGGTAGGAGCTATCTTTCT-TACTATGTAAATAACA 1759

CrLFY2 GATCTTTTGTGGTAATCCAATTGCCAGTAGTGGCTTTGTTTTAAAGCCATGTACATACTA 1623

* * ** **** * ** **** *** * *** * ***** *** *

CrLFY1 GTTGAACAGTCAAATGTTGAATGGCAATTCGTAAACGAATGTATAGGAT-GCATTTCGAG 1818

CrLFY2 GTGAAGTGGGCTAATTGGTCGGAGCAAGCAATGAGAATGTTTATGGCATTGGCAGTCAAG 1683

** * * * *** **** * * * *** * ** * ** **

CrLFY1 GTCCTTTTCTTGCTAATGATGGCATGATCA-AGGCAGAACTTCGTGTCCTTTGTAGGTTT 1877

CrLFY2 GTGCCA---TTGCTAGCAAGGTTATAGCTTAAGTTAAATCTCGGTGTCCT-TGTTGATGA 1739

** * ****** * * ** ** * * ** ******* *** * *

CrLFY1 GTTTATCTGGGAATTAGCAGAAACATTGAGAGGTTC--------ATATTTCCTAACCGAG 1929

CrLFY2 CATTTTTTGCTTAGGAGTGCACCTGTTGGAATGTTGTTGGTGTCTCATTGCTAGCCAGAT 1799

** * ** * ** * *** * *** *** * * **

CrLFY1 CAAATAGCAATCCTTAGTTTCTTACTGTGCATAAGTACATAATCTGATAGGTAGGATA-- 1987

CrLFY2 TATAGCTTAAGTTAAATCTCGGTGTCCTCCCTGATGACATTTTTTGCTTAGGAGTGCACC 1859

* * ** * * * * * * * **** * ** * * ** *

CrLFY1 ---------TGAGATAACCTTACCTATGTGATAACCTGACTTTGGGTGT----------- 2027

CrLFY2 TGTTGGAATGTTGTCTCATTTACCTTTGTATTCAGTTGCCTATAGGTTTTAGTTTCAAAG 1919

* ****** *** * * ** ** * *** *

CrLFY1 ----ATGGAGAGTATGTGTATCATTTTCAAGTTTGTTTGAAA------------ 2065

CrLFY2 CAGTGTGCAGGATGTATGAATGATCTCCGATTCTGTGATTCATAGTGGATCCAA 1973

** ** * * ** ** ** * * * * *** *
