# Supplementary material for: LEAFY maintains apical stem cell activity during shoot development in the fern Ceratopteris richardii
Source: eLife. 2018 Oct 24;7:e39625. doi: 10.7554/eLife.39625 (PMC6200394; doi:10.7554/eLife.39625)
Supplement: Supplementary file 6. — Alignment (prepared using Clustal Omega) of full length CrLFY1 and CrLFY2 transcript sequences, with nucleotide identity between the two paralogs denoted by a subtending asterisk. The coding sequence (CDS) for each gene copy is highlighted in bold. Predicted sites of hybridization for the two probes are highlighted in blue (CrLFY1) and yellow (CrLFY2) respectively, with PCR primer sites underlined. The in situ probes span the complete CDS and 5’UTR of each gene copy. The CrLFY1 probe sequence shows 79% nucleotide identity to the CrLFY2 transcript (BLAST2n, discontiguous megablast for highly similar sequences). The CrLFY2 probe shows 79% nucleotide identity to the CrLFY1 transcript (BLAST2n, discontiguous megablast for highly similar sequences). [file elife-39625-supp6.docx]

**Supplementary File 6. Predicted hybridization and specificity of *CrLFY* *in situ* hybridization probes.** Alignment (prepared using Clustal Omega) of full length *CrLFY1* and *CrLFY2* transcript sequences, with nucleotide identity between the two paralogs denoted by a subtending asterisk. The coding sequence (CDS) for each gene copy is highlighted in bold. Predicted sites of hybridization for the two probes are highlighted in blue (*CrLFY1*) and yellow (*CrLFY2*) respectively, with PCR primer sites underlined. The *in situ* probes span the complete CDS and 5’UTR of each gene copy. The *CrLFY1* probe sequence shows 79% nucleotide identity to the *CrLFY2* transcript (BLAST2n, discontiguous megablast for highly similar sequences). The *CrLFY2* probe shows 79% nucleotide identity to the *CrLFY1* transcript (BLAST2n, discontiguous megablast for highly similar sequences).

*CrLFY1* GTGCGAGGCATACACACACGCAGTCCTGGTGTGCCTTTCCTGTTCTAGTACTCATACAAA 60

*CrLFY2* ------------------------------------------------------------ 0

*CrLFY1* TACGACGTGCGCTTCCTTTTCTTCCTACCTTTGCTTTCAAATTGACGCATCTTCTGGTCT 120

*CrLFY2* --------------------------------------------------------AGCT 4

**

*CrLFY1* TCACTGCTTGTGAAGCAGGTTGTCACAAAAAGTTCCTGCTGCTTAT----GTTTACTCCA 176

*CrLFY2* CGCGAGAAGCAAGGGCTGGTTGTTACGGATAGCGGGCACAATTTAGGGTTGCACGCAGCA 64

* ** ****** ** * ** * *** * * **

*CrLFY1* GCGGTTCTTGTAATTGTGTCACCACAGCTGCTACCCGGAGTTACTCTGCAGTATCGACGC 236

*CrLFY2* GGTGATTCTGAATTTGTATCATCATCGTTGGAGTGCGGATTCACTGTGCAATAGTAGATT 124

* * * ** * **** *** ** * ** **** * *** **** **

*CrLFY1* AATTCTCCTT--------CTACGTAGCAGAAGCAGTGCATGACCTTCGATATTGTTCGTT 288

*CrLFY2* CTCCCTCCTTTACAGGTTTCCTGGAACGACAACGGATCATAACCATCGGGTTCTTTCGGT 184

****** * * * * * * *** *** *** * **** *

*CrLFY1* TACACAGGTGAAGTTTGGCCTTCTCGGAAATTACAGACTATCTCCTCGTTCTCAAATGAA 348

*CrLFY2* T---------------------------------------------------------AA 187

* **

*CrLFY1* AAGCGGAGTTTTATCACT---TCACTGAAGTCGGGCATTGTCCGTGAAAGTCTCACCGAC 405

*CrLFY2* CGAAGGAATTTGGTCGGAAGCTCTGTATCAGACATCCTAGTCACTGTTCTCATCCCTCTT 247

*** *** ** ** * * * *** ** ** *

*CrLFY1* CGAAAGAAGTCGATCATTTCTTGTACTGCTCTCTATCACTTCAAGCTGGATCCTGAACAG 465

*CrLFY2* TGGTCTTCCTCCTCCACTAAAGGTGCTGCTATCAACCAGATCAAGCTGGATCCTGAGCAC 307

* ** ** * ** ***** ** * ** **************** **

*CrLFY1* TTTGCTAGTTCTTTGTTCCGGTGGGAACAAAGGGCTATCCCACGTAAAGAAGTGCCTCCA 525

*CrLFY2* TTTCCTGGTCCC**ATGTTCCGATGGGAACAAAGGGTAATACATCGTAAGGAGGTCCCCTCT** 367

*** ** ** * ******* ************* ** * ***** ** ** ** *

*CrLFY1* **ATGGATGTCTCTTTATTGCCACCAACCACCACCA------------------------TC** 561

*CrLFY2* **ATGGAGGCTTCCATACTTCCTCCAACCACCACCACCACCACTGCTGCTACCTCTGTGACA** 427

***** * ** ** * ** *************

*CrLFY1* **GCTGGTACTGCAGATCCGAAACAACTGAAACTTCTTGAAGATCTCTTTAAAGACTATGGA** 621

*CrLFY2* **ACTGTTGGGACGTATCCAAAACAGCTTAAACTCCTTGAAGATCTCTTCAAGGACTATGGT** 487

*** * * **** ***** ** ***** ************** ** ********

*CrLFY1* **GTACGAAGCACCACAATAATTAAGGTCATGGAGATGGGTTTCACTGTGAATACTTTGGTG** 681

*CrLFY2* **GTACGGAGCACTACCATAGTGAAGGTTATGGAAATGGGTTTCACTGTTAGTACTCTAGTG** 547

***** ***** ** *** * ***** ***** ************** * **** * ***

*CrLFY1* **AATATGATGGAGCAAGAGATTGATGACCTAATCAAGACCATGACAGAAAGCTACCATATG** 741

*CrLFY2* **AACATGATGGAGCAAGAGATTGATGATGTTATCAAGACAATGATAGAGGGTTATCATATG** 607

** *********************** * ******** **** *** * ** ******

*CrLFY1* **GAGTTGTTGGTCGGAGAGAAGTATGGCTTGAAGTCCGCTATTCGTGCAGAGAAGAAGCGG** 801

*CrLFY2* **GAACTTCTGGTTGGTGAAAAATATGGTTTGAAATCTGCAGTCCGGGCTGAGAGAAAACGC** 667

** * **** ** ** ** ***** ***** ** ** * ** ** **** ** **

*CrLFY1* **CAAGAAGAGGATATGGAACGCCAACGATTACAACTGCTAGCAACAAGTAGCAAAAAACAT** 861

*CrLFY2* **CAAGAAGAGGAGATGGAACGTCAACGACTGCAGTTATTGGTAAAAAACAGCAAAAAGCTC** 727

*********** ******** ****** * ** * * * ** ** ******** *

*CrLFY1* **AAGTCAGATGAAAGCGGCATGGTTGTAACTTCACTTGAAGGCACAAGGGAACAAAGAGGA** 921

*CrLFY2* **AAGTCAGATGATAGTGGCATGATAGCAGTGTCTGTAGAAGGCACCAGGGAACAAAGAGGA** 787

*********** ** ****** * * * ** * ******** ***************

*CrLFY1* **GATAATGTTATGATGTTTCCAGAGGCTGTTGCACCCAATGCCCCCTTGAATTTGAACTCG** 981

*CrLFY2* **GACAATGGTATGATGTTTCCAGATACTGCTGCTCAGAATGGTCCCTTGAATCTGAACTCA** 847

** **** *************** *** *** * **** ********* *******

*CrLFY1* **AAAGATCATGTCCAACAAGAGCATAGCCATGCACAAATAGGGCCACCTGGACTCCTGGCT** 1041

*CrLFY2* **AAAGATCATGCTCAGCATGAGCATAGCCATGGCCTGTTTGGACCACCTGGACTCCTTGCT** 907

********** ** ** ************* * * ** ************** ***

*CrLFY1* **CTACCAGAACCAAGCAGTGACAATGAGGGTCACAAATTACCAAGAAAAAAGCCGAAACGG** 1101

*CrLFY2* **CTACCTGAACCTAGCAGTGACAATGAAGGACGCCAAATACCAAGGAAAAAGCAAAAACGA** 967

***** ***** ************** ** * * ** ******* ******* *****

*CrLFY1* **AGGCTGTTGCGAGAACCTGGTGAGGACGGTGATGACAGAACAAGAGAGCATCCATTCATT** 1161

*CrLFY2* **AGGTTATCGCGTGAGCCTGGTGAGGACGGGGATGACAGGACCAGAGAACACCCATTTATA** 1027

*** * * *** ** ************** ******** ** ***** ** ***** **

*CrLFY1*  **GTGACAGAGCCAGGTGAAGTGGCAAGGGGAAAGAAGAATGGTTTAGACTACTTGTTTGAT** 1221

*CrLFY2* **GTGACAGAGCCTGGTGAAGTTGCAAGGGGAAAGAAAAATGGCTTAGATTATTTGTTTGAC** 1087

*********** ******** ************** ***** ***** ** ********

*CrLFY1* **TTATACGAACAATGTGCACGCTTTTTAGATGAAGTGCAACAGATGGCCAGAGAAAGAGGG** 1281

*CrLFY2* **CTGTATGAGCAGTGTGCACGATTCTTAGATGAAGTACAACAGATGGCAAGGGAAAGGGGG** 1147

* ** ** ** ******** ** *********** *********** ** ***** ***

*CrLFY1* **GAGAAGTGTCCTACGAAGGTAACAAATCAAGTATTCCGGCATGCCAAGTTGAAAGGTGCG** 1341

*CrLFY2* **GAGAAATGTCCTACAAAGGTAACAAACCAAGTGTTTCGACATGCCAAGCTCAAAGGTGCA** 1207

***** ******** *********** ***** ** ** ********* * ********

*CrLFY1* **AGCTATATTAACAAGCCAAAGATGAGACACTATGTACACTGCTATGCACTGCATTGTCTG** 1401

*CrLFY2* **AGTTATATCAACAAACCAAAGATGAGGCACTATGTTCACTGCTATGCCCTACATTGTCTG** 1267

** ***** ***** *********** ******** *********** ** *********

*CrLFY1* **GACAAAGAGAAATCAAACTTTTTGAGAAAACAGTTCAAAGAGAGGGGAGAAAATGTGGGT** 1461

*CrLFY2* **GATAAAGATAAATCAAACTTCTTAAGGAAACAGTTTAAAGAAAGAGGAGAGAATGTGGGT** 1327

** ***** *********** ** ** ******** ***** ** ***** *********

*CrLFY1* **GCATGGCGACAAGCATGCTATTATCCATTGGTGGATATGGCTCGAGATAACGGCTGGGAT** 1521

*CrLFY2* **GCATGGAGACAAGCTTGCTACTTCCCTCTGGTTGACATGGCTAGAGACAATGGTTGGGAT** 1387

****** ******* ***** * ** **** ** ****** **** ** ** ******

*CrLFY1* **ATAGAGGGTGTCTTTGCGAGGAATGAGAAGCTCCGTATCTGGTATGTTCCTACAAGGCTT** 1581

*CrLFY2* **ATAGAAGGTGTCTTTGTCAGAAATGAGAAGCTTCGGATTTGGTATGTCCCAACCAAACTT** 1447

***** ********** ** *********** ** ** ******** ** ** * ***

*CrLFY1* **CGACAGCTCTGTCATTTAGAGAAGAGCAAGGACAGTGATAGCTGCATCTATGATTGA**AAT 1641

*CrLFY2* **CGCCAACTTTGTCATTTTGAAAAGAGCAAGGACAGTGACAGCTGCAGCTATGAATAA**GTA 1507

** ** ** ******** ** ***************** ******* ****** * *

*CrLFY1* TGGCTTCATTACT-GCACTTTGTAATATGTGGATCAAGCACACGACTGCATCATGATATC 1700

*CrLFY2* CATACCTGAGGTGTTGTTTTTGTAATTTATCAATTCAATGCACG----TTTCACCACAGG 1563

******** * * ** * **** *** * *

*CrLFY1* CCCGTATCATGATAATGCCTATGATGGTAGGAGCTATCTTTCT-TACTATGTAAATAACA 1759

*CrLFY2*  GATCTTTTGTGGTAATCCAATTGCCAGTAGTGGCTTTGTTTTAAAGCCATGTACATACTA 1623

* * ** **** * ** **** *** * *** * ***** *** *

*CrLFY1* GTTGAACAGTCAAATGTTGAATGGCAATTCGTAAACGAATGTATAGGAT-GCATTTCGAG 1818

*CrLFY2* GTGAAGTGGGCTAATTGGTCGGAGCAAGCAATGAGAATGTTTATGGCATTGGCAGTCAAG 1683

** * * * *** **** * * * *** * ** * ** **

*CrLFY1* GTCCTTTTCTTGCTAATGATGGCATGATCA-AGGCAGAACTTCGTGTCCTTTGTAGGTTT 1877

*CrLFY2* GTGCCA---TTGCTAGCAAGGTTATAGCTTAAGTTAAATCTCGGTGTCCT-TGTTGATGA 1739

** * ****** * * ** ** * * ** ******* *** * *

*CrLFY1* GTTTATCTGGGAATTAGCAGAAACATTGAGAGGTTC--------ATATTTCCTAACCGAG 1929

*CrLFY2* CATTTTTTGCTTAGGAGTGCACCTGTTGGAATGTTGTTGGTGTCTCATTGCTAGCCAGAT 1799

** * ** * ** * *** * *** *** * * **

*CrLFY1* CAAATAGCAATCCTTAGTTTCTTACTGTGCATAAGTACATAATCTGATAGGTAGGATA-- 1987

*CrLFY2* TATAGCTTAAGTTAAATCTCGGTGTCCTCCCTGATGACATTTTTTGCTTAGGAGTGCACC 1859

* * ** * * * * * * * **** * ** * * ** *

*CrLFY1* ---------TGAGATAACCTTACCTATGTGATAACCTGACTTTGGGTGT----------- 2027

*CrLFY2* TGTTGGAATGTTGTCTCATTTACCTTTGTATTCAGTTGCCTATAGGTTTTAGTTTCAAAG 1919

* ****** *** * * ** ** * *** *

*CrLFY1* ----ATGGAGAGTATGTGTATCATTTTCAAGTTTGTTTGAAA------------ 2065

*CrLFY2* CAGTGTGCAGGATGTATGAATGATCTCCGATTCTGTGATTCATAGTGGATCCAA 1973

** ** * * ** ** ** * * * * *** *
