# Supplementary material for: LEAFY maintains apical stem cell activity during shoot development in the fern Ceratopteris richardii
Source: eLife. 2018 Oct 24;7:e39625. doi: 10.7554/eLife.39625 (PMC6200394; doi:10.7554/eLife.39625)
Supplement: Supplementary file 7. — Sequence highlighted in green corresponds to published CrLFY2 5’UTR (Himi et al., 2001) [file elife-39625-supp7.docx]

**Supplementary File 7**. **Amplified *CrLFY2* genomic fragment (3619bp), not connected directly to *CrLFY2* open reading frame.**

AGATCTTTCTCTATATCTCATTATGCTCTTCTCACCTATTTCTTGCATATATACTATTGCACATTGAACCTATTCCTTGGTCTCGATTATCCAAAATAATATAGAGAGCTCAATTCTAGCAATTTTTCCACGTAACTACCACCAAAAGGACTTTGATTGTTGAGTCTCTATATGTTCACTTTtGTCTTGGTATTCGTGGTATGTATGCAAATTAATGAGCAACTTTGATTCCGTTCTATATCCCTTTATGGAGCTCCATCTCCATGTATTGAGCTTCTCATTACACCATGCCTTTCTTAGTGAAGCTTCGAAAGATCATGTCATAGAGATCCAAGTGATTGCTTGCTAATATTGAAGAAGTTTTAGCCTGAAGTAAGTAGCCCATCCTTTTATACAGATTTGGCACCTAAGTGGCCAAGCTAGAAATGAAAACTCTAGAAAAAGAGTAAAATAACCACAAATTCCTTCAAGTTCTAGAAAGTAAGGAAATCATGCCCATACTTAGGCTACCAACACCTTGAACCCCATGCAATCCAAGGCTTGCTAACAAATTAGCTTAACCTTTGAGCGTTCATAACACTACCAAAGCAAGGAAAGAATGTCCATTATAAGGTTAGCCATAGCTTGAAGCTATATGAGCAAAGAGTGAATCCTATCCACCAAATTAGGCTTGCTATAAACTATTTTATCTTTTGATAGCTTGATATCACCACCTTAGGAGATGCCTTCAAGAAAAGAAAAATGTGTTTAGAAAAGATCACATAGTGGAGGTCATTAAGGTGTCCTTTTCTTCCTCCACATGTTTTTGAGGTCATCAAACTTCTCTTTTCTCCCCCAATAGGAAACACAAAGACTTCATTTATCAGTCCATAGCCTGTAGGTTAGGATCTAGCCTCTCTCTCTAGGCTCTTTCGTCCTTAGTCCGCATCCCTAGGGCCAATGTAGTATGGTTTCTTCTGTCAATATTTCGTGTGACTTCACTTGGGAGAAAGAACACCTTTCAAGTACCTCAAACATTTATACAGGGATTGACATGTTGGAAAATGACGAAGTATCTTTTCCACGAGAGACAGAGAGAGAGAGGGAGAGAGATGAGTGACGAGTCTGTTAGCGAGGAGAGGATGTGTGCGTGTGAGAGCGTTGGCTTTAAGTATCGAAACTATGGCAGTGTGTTGCAGATGGTCGAGGCACGCGTTGTACTTGGTGAAAGGATAGATGAGATGGAGATGGGCTTATTCCAAAGAAGGGCCCAGGGCAAGACCATGTTCGCTTCCGTGAGGCAGCGGAAGAGTCTGCGGATACAAGGGTGTTTACCCCCCTGTCTCCACCTTGAAACTGACGGACATTCAAAGTTGCCCATTGGATGCCAGCGCCTCTTCTCTTCCTCTTTGCGTTGCATCCGACCGATGTTGCTCATGTCCTCCGGATCTTGGGACTGTCAACACTCACGGTCTTGTCCAGCGAGCAAAAGCGCGCAGGATGGCCTCTTGAAGCAGACAGCTGAAGGAAACGAGAGCTATGGATCTTGTAGTTTCTCGGTTTTATCTAGTGGGCGACGGAGTTTGCCACAGGCTTCAGCTCGCGAGAAGCAAGGGCTGGTAGGCAGCCCCCAACCCCCCCTCTCTCTCTCTCTCTCTCTCACTCTCTCTCTCCCTCGTTCGCTCGCTTCCGTGCGCTTCTCTCCTCGCATTCACGCGCCATAGGCACTTGCATATTGTTTGATTTAGCATTCAGGTCTCGGTTTGCTCCCAAATTAACGGCTCTGCTCTTTCCGCCTCGTCCTGACGCAGGTTGTTACGGATAGCAGGCACAATTTAGGGTTGCACGCAGCAGGTGATTCTGAATTTGTATCATCATCGTTGGAGTGCGGATTCACTGTGCAATAGTAGATTCTCCCTCCTTTACAGGtttcctggaaCGACAACGGATCATAACCATCGGGTTCTTTCGGTTAACGAAGGTACATTCTGGGATTTTCCGTCCTGTCACCACACGTCGTGTTCTTTCCTTCTCTCTTTCATGCTCCTAGGAAGCAAAGCAAGCCCTCTCAACACTTTTTAGCCTCTAATTTGCCTCAGACGCACACTGTTTGTTCTTAATGTATCTTAGCGTCCTAGAACGATTGGATGTCTTGTTTTCATGAATTCTCTCTATCCAGATGCTGAACGCTTCTCGGTTATCTAGTACCAGTACTTGCTTTGTCGGGGATTATGAGCTTACATTTATAATCGTGAGGCTACTACTATTATATCGAGCTCGTCTGTTACGTTTATAACATGTTGAGGTCGATGTTGCTGGGATGATTGTTGGGGATGTATAACTCGAATCACTTCTCTATCGATGAAGTGAACTTTATGGATCGGTTGCGTGCTTTTCCTCCTTTCGTACAGCAGCAGTATTAGTAAAACGTCAGCTCTTCATTTCTCTGAATTATCGAATGCTAAATAACTGTCTCCCTTGCGATATTCGAGCTGTTGTCTTGTTCTGATGTCTTCTGAATCTTCAGTAGATTGTTATCTGATTGTTCCTTCCTTAGAGTTACCAAGTAAAAAGTATTGCAGTAATCGAGCTGCCGCAGGTTCTGCCACGCATACTGGATACTGACTTATTGGTACTCTGTAATCTATATCTGTTTTGTCAATTCACTGAAAAGAAATATTTTCTGTACTGTTTGCTGTTTGCCTATCCGACTAGTTTCCGTTTGGATGAAGGTTGCTCGAGCGTGCATTCAAACTTAATTGTTAATTCGCTTATGTATGAGTGGGCAAGTGCTTAGGCTTCATAATTCTGCTGTAAGTCTGACGGCCTCATGTTGTCTAAAAACAAGGCATGGCCTGATATGACGTCCGATTGCCTTGTCATAATAATCAAGCCGGTAGCTTTCCTGCAATGACTAGATTGGTAGGCCTGAGACATTGACCCATTCATCTTGTTTCACTTGAATATTGATATGCATAGAAGACATCGTCATTCTTTATCAGTTTGGGGCTGCTTGTTTTTTGTGAATATTCTCTAGCCAAGAGTTGATGCCCAATCCTTACACTGCAATTTGCATATTGATTTTATGCTTGGCCTAGCTACATTGCTCCACTGTGAAGTTAACTGTTGGGGGAAAAGTATATCTTATCAAATGTATTAGAAGCTCTTAGGAGCTGTCTGTGGACTCAAATAGGTTATCACCGAGAGCACTGTTCTTAGCATGGTTGAATAAACAATTCAATCATTTAGTGATTACACCTTGAAGCTCTCCGTTGAGCTCAGGACTAAATCCAATTGAAGTTCTCACTGGAGAACTTACTAGTGGTTTGAGTGCGAACTTTAGAAAAGTTGAAATAAGCATACCTGTTTGGCGGCAGCTATCACCACACCTGAATTGATGCCTTAGTACAGCAAATTAGCTGCAAAATCGCTCTTTAATTTGAATCACGTGTGATATTGGCATATTTTGATTGCATAAAAGCTTTCATGGGCGATAGATCTCTCAATTGTGGTGAGCCATTTTGAGTCCTGAGTGATGTTGTGCTATCATAGATGATGCCAGTATACTCCTAAAAATTACTGATTTTGAAATCAATAAGTGAACAATGTGCATGCGACTCTGGGGATTTCAGGGACTAGT
